# Identifying Key Features of Polyoxometalate Catalysts for the Anaerobic Conversion of Glucose Into Lactic Acid

**DOI:** 10.1002/cssc.71192

**Published:** 2026-11-10

**Authors:** Elisabeth Hundt, Pegah Saedi, Lasse Prawitt, Andreas H. Pawlig, Jan‐Dominik H. Krueger, Maximilian J. Poller, David Robinson, Jakob Albert

**Affiliations:** ^1^ Institute of Technical and Macromolecular Chemistry Universität Hamburg Hamburg Germany; ^2^ Department of Physical Sciences School of Science and Technology Nottingham Trent University Nottingham UK

**Keywords:** biomass, green chemistry, homogeneous catalysis, lactic acid, polyoxometalates, retro‐aldol reaction

## Abstract

The effective valorization of biomass to value‐added platform chemicals is one major aim of the green chemistry transition. A particularly promising compound in this context is lactic acid (LA), which is already extensively used in the chemical industry. This study investigates key features required for the homogeneously catalyzed transformation of glucose into LA by studying a library of polyoxometalate (POM) catalysts, with variations in structure, elemental composition, and countercation. The highest yield of LA (25 %) was obtained with the Keggin‐type vanadium‐substituted phosphotungstic acid H_6_PV_3_W_9_O_40_. Experimental and computational studies revealed that this performance correlates with high Brønsted and low Lewis acidity/fluoride ion affinity. Additionally, the catalyst exhibits a unique redox behavior as shown by square‐wave voltammetry. Further investigations of the reaction pathway revealed these characteristics affect mainly the retro‐aldol reaction of fructose to dihydroxyacetone, which was identified as the selectivity‐determining step. Overall, this study contributes to the transition to a green chemical industry by providing new insights into an efficient production of the sustainable platform chemical LA.

## Graphical abstract

The valorization of glucose as a model for biomass into high‐valuable platform chemicals such as lactic acid (LA) is an important goal of green chemistry. Polyoxometalates (POMs) are the most promising homogeneous catalysts for this reaction. To gain a deeper understanding of their catalytic activity, the significance of various key features of POM catalysts was investigated using a variety of analytical methods.

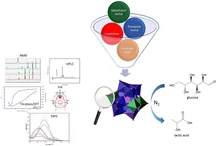

## 1 Introduction

A key goal of green chemistry is to limit dependence on fossil resources and reduce CO_2_ emissions. Consequently, both industry and academia have pursued the development of low‐energy processes that emit less CO_2_ and use renewable, nonfossil feedstocks. In this context, biomass has emerged as a promising and widely available renewable carbon source for the production of value‐added chemicals [1].

One such value‐added platform chemical is lactic acid (LA). As reported by Dusselier et al., LA is extensively used in various industries, including cosmetics, chemical manufacturing, and food production [2, 3]. Currently, approximately 90 % of LA is produced via fermentation processes. However, this approach is associated with several drawbacks, such as low space‐time yield (1.79–5 g L^−1^ h^−1^) [4, 5, 6], high costs (depending on which fermentation process, $993–1392 per ton) [7, 8], and sensitivity to pH fluctuations [9, 10, 11, 12]. To overcome these limitations, several catalytic routes for the chemical transformation of biomass to LA have been investigated such as homogeneous [13] or heterogeneous catalysts [14, 15], as well as different solvent systems [16].

The chemical conversion of glucose to LA, as shown in Figure ‍1, involves a series of chemical reactions, beginning with the isomerization of glucose to fructose. Herein, epimerization leading to mannose formation should be avoided. For the isomerization, a 1,2‐intramolecular shift is needed [17]. Therefore, several studies have shown a positive effect of Lewis acids and an alkali‐free catalytic system [17, 18]. Subsequently, a retro‐aldol reaction to dihydroxyacetone (DHA) and glyceraldehyde (GLAD) follows. Both are linked via keto‐enol tautomerization by removing the α‐proton of DHA by a base to form an enediol anion [19, 20]. To overcome the activation barrier of these reactions, it is necessary to employ a temperature of ≥120 °C in combination with a heterogeneous catalyst [15, 20]. Dehydration of both DHA and GLAD leads to the formation of pyruvaldehyde [16, 19, 20, 22]. Subsequently, formation of LA occurs by addition of water in combination with a 1,2‐hydride shift. As described by Megías‐Sayago et al., the formation of pyruvaldehyde and LA necessitates Brønsted and Lewis acidity of a catalyst [23].

**FIGURE 1:**
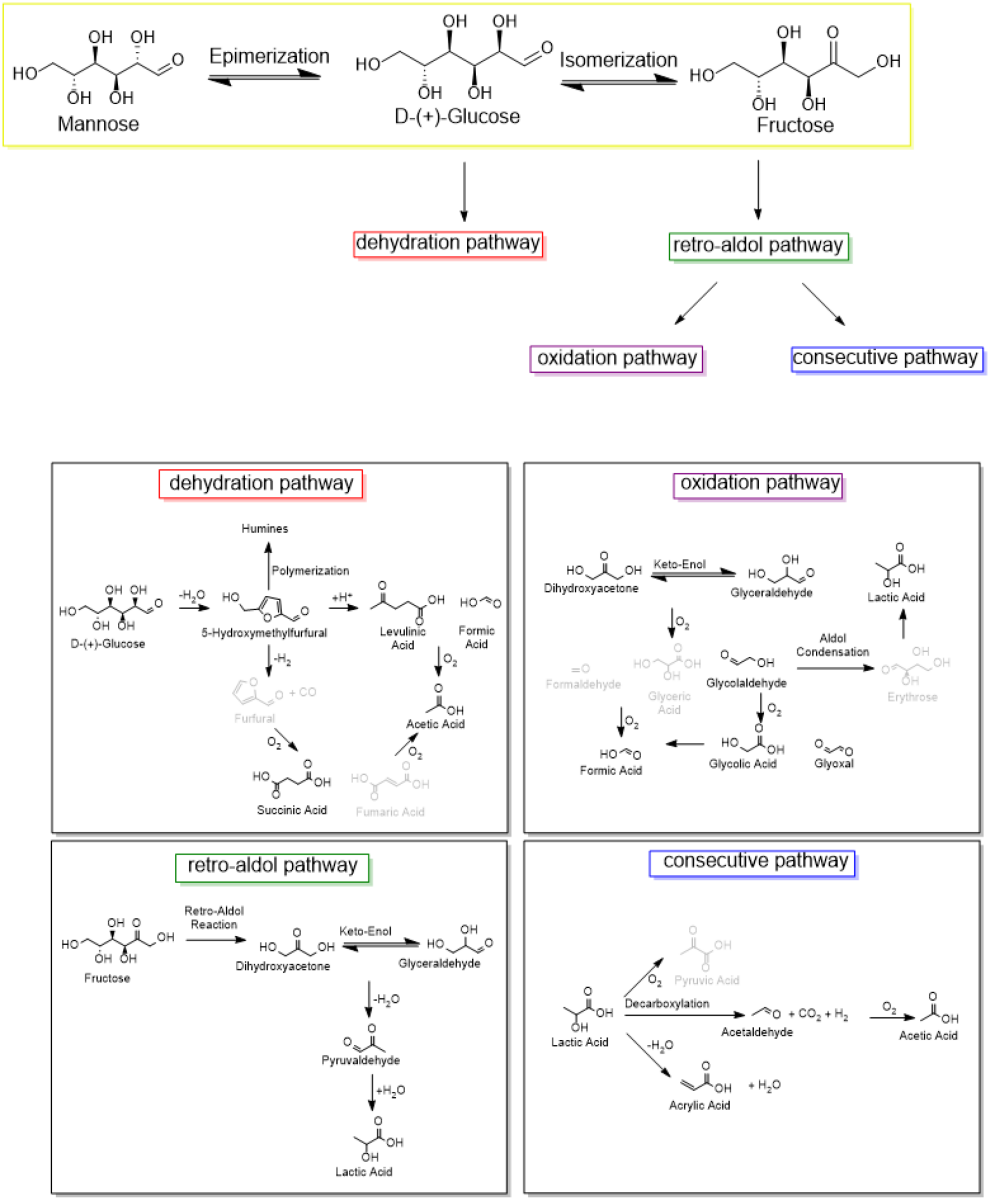
Reaction pathway from glucose to lactic acid (retro‐aldol pathway, green) and other products like 5‐HMF (dehydration pathway, red), acetaldehyde (consecutive pathway, blue), and glycolic acid (oxidative pathway, purple) [16, 17, 18, 19, 20, 21].

However, elevated temperatures have the potential to induce dehydration of glucose to 5‐hydroxymethylfurfural (5‐HMF) [24]. This initial undesired reaction may subsequently be followed by an acid‐catalyzed formation to levulinic acid (LevA) and formic acid (FA) [21, 24, 25, 26, 27, 28, 29]. The most significant engineering issue is the polymerization of 5‐HMF, forming solid humins [30]. It is imperative to acknowledge the potential adverse repercussions that humins may have on the integrity of reactor components [30].

Another undesired pathway is the oxidation of GLAD to glycolic acid (GA) and glycolaldehyde. As posited by Liu et al., glycolaldehyde undergoes a subsequent oxidation process, resulting in the formation of GA and glyoxal [30, 31]. The value of GA as a product has been demonstrated in a variety of applications [31].

As demonstrated by Corma et al., the sequence of reactions involving LA can result in the formation of pyruvic acid by oxidation, acetaldehyde (AcetAl) via decarboxylation, or acrylic acid by dehydration [32, 33].

A wide range of heterogeneous catalysts, including zeolites and metal oxides, have been explored for the formation of LA [34, 37]. Although high selectivity for LA has been reported, these systems often require long reaction times or harsh operating conditions. An example for a mild reaction with heterogeneous ZnO‐catalysts was carried out by Paulino et al. (30 bar N_2_, 140–180 °C, 30 min), where a glucose conversion of 64 %, a LA yield of 12 %, and a LA selectivity of 43 % were achieved [37]. However, the disadvantage of a heterogenous system is the two‐phase reaction in which activity of the catalyst and conversion are limited by mass transfer, i.e. diffusion of the molecules to the catalyst surface. This problems can be overcome by using a homogeneous catalyst. For example, Paulino et al. showed that the heterogeneous ZnO‐catalyst exhibited slightly lower yields than homogeneous catalysts such as VOSO_4_ [28], NH_4_VO_3_ [26], H_4_PVW_11_O_40_ and H_8_PV_5_W_7_O_40_ (both Keggin‐type polyoxometalates (POMs)) [26], and K_5_[V_3_W_3_O_19_] (Lindquist‐type POM) [38]. K_5_[V_3_W_3_O_19_] yielded 17 % LA after 1 h (20 bar N_2_, 160 °C), representing an improvement of 2 % in yield compared to the commercial catalyst VOSO_4_ [38] and 5 % yield compared to the heterogeneous ZnO‐catalyst [37].

POMs generally follow the formula [MO_*x*_]_*y*_, where M represents transition metals such as tungsten or molybdenum. In this study, different structure types were used. As mentioned above, one possible structure type is the Lindquist‐type with the general formula [M_*x*_M’_6−__*x*_O_19_]^*n*−^, where M’ denotes substituted metals such as vanadium or niobium [39], and M represents framework metals [40]. Other prominent POM structures are Keggin‐type [XM_12_O_40_]^*n*−^, Wells‐Dawson‐type [X_2_M_18_O_62_]^*n*−^ and Anderson‐Evans‐type [XM_6_O_24_]^*n*−^ [40]. These four POM types are the most commonly used structures in catalysis [41, 42, 43]. The heteroatom X may be, for example, phosphorus or tellurium. Partial substitution with vanadium leads to modified Keggin‐type structures of form [PV_*n*_M_12−*n*_O_40_]^(3 + *n*)^ [40]. Besides the choice of counterions like sodium, potassium, or protons, which strongly influences the acidity of the POM, the selection of substitution degree and framework metals significantly affects the redox potential of the catalyst [44, 45].

This study investigates the effects of structural type, transition metals, substitution metal, counterions, and oxidation states of the catalyst to gain a deeper understanding of how the POM structure influences the anaerobic reaction pathway and its regulation. We use the knowledge generated about key parameters of the POM structure to facilitate optimization of the catalyst system and reaction setup.

## 2 Experimental Details

### 2.1 Catalysts Used

In this study, a systematic selection of POM catalysts with variations in structure type, substitution metal, transition metal, and counterion was investigated. Four different structure types were used and compared: Keggin (Na_6_PV_3_W_9_O_40_, K‐NaVW), Wells‐Dawson (Na_8_HP_2_V_3_W_15_O_62_; WD‐NaVW), Lindqvist (Na_5_V_3_W_3_O_19_; L‐NaVW), and Anderson‐Evans (Na_5_H_4_TeV_3_W_3_O_24_; AE‐NaVW). Active metal comparison was performed by using two Keggin‐type catalysts, Na_6_PV_3_Mo_9_O_40_ (K‐NaVMo) and Na_4_H_2_PNb_3_Mo_9_O_40_ (K‐NaNbMo). The framework metal tungsten was changed to molybdenum for the Keggin‐type POM K‐NaVMo and the Wells‐Dawson‐type POM Na_7_H_2_P_2_V_3_Mo_15_O_62_ (WD‐NaVMo). To investigate the influence of the counterion, the Keggin‐ and Wells–Dawson‐type POM containing two different framework metals were used in their protonated forms, H_6_PV_3_W_9_O_40_ (K‐HVW), H_6_PV_3_Mo_9_O_40_(K‐HVMo), H_9_PV_3_W_15_O_62_ (WD‐‍HVW), and H_9_P_2_V_3_Mo_15_O_62_ (WD‐‍HVMo), respectively. All these materials were synthesized according to previously published procedures [39, 46]; detailed descriptions of the synthetic procedures can be found in the Supporting Information (Section Catalyst Synthesis).

### 2.2 Catalyst Characterization

The successful synthesis of the catalyst materials was confirmed by elemental analysis via inductively coupled plasma optical emission spectroscopy (ICP‐OES) and ion chromatography (IC), as well as infrared spectroscopy (IR) and nuclear magnetic resonance spectroscopy (NMR). Further details on the instrumentation and the results of these measurements can be found in the Supporting Information (Section Catalyst Characterization and Details of Analytical Procedures).

### 2.3 Procedure for Catalyst Reduction

For the reduction of vanadium within the catalyst K‐HVW, 0.5 g (0.2 mmol, 1 eq.) of the oxidized catalyst was dissolved in 100 mL of demineralized water. To the solution, 0.953 mL (1.2 eq.) of a 72 mmol/L hydrazine monohydrate water solution was added under stirring at room temperature. The content of vanadium and the required amount of hydrazine were calculated based on the elemental analysis of the catalyst. The mixture was stirred for ten minutes at room temperature. Then, it was centrifugated at 10,000 rpm for 4 min and filtered off. Afterward, the solution was transferred to a round bottom flask and the solvent was removed under reduced pressure (85 °C at 80 mbar) for 2 h. Subsequently, the yielded powder was further dried under vacuum at 0.2 mbar for 1 h at 130 °C, yielding 0.38 g (0.15 mmol, 76 %) of a black powder, which was identified as reduced H_6_PV_3_W_9_O_40_ by IR spectroscopy, NMR, and elemental analysis. The final product was stored under a nitrogen atmosphere to prevent reoxidation.

### 2.4 Experimental Set‐up for Catalyst Screening

The experiments were conducted using a 10‐fold screening plant. The flowchart of the plant is shown in Figure S14 with further information on the plant. In a typical catalyst screening experiment, beakers were filled with 10 mL of demineralized water and 0.18 g (1 mmol, 1 eq.) of glucose. Catalyst mass was determined based on the elemental analysis of the catalyst, so that 0.1 mmol (normalized to the vanadium content) of each catalyst was added to the substrate solution. The masses of each reagent are listed in Tables S2, S5, S8, and S11, respectively. The solution was then homogenized by stirring at 300 rpm for a period of 10 min. Followingly, the solution was transferred to the reactors. The reactors were sealed and positioned in the heating block, and subsequently, the stirring was set to level four. Then, the reactors were purged three times with 10 bars of nitrogen to eliminate oxygen. This pressure was maintained for several seconds before being released again. The fourth time, the reactors were set to an initial pressure of 12.5 bar. After this, all reactors were closed and heated to 200 °C. Upon attaining the desired temperature, the stirring plate was adjusted to level six, and the nitrogen pressure in the reactors was 20 bar. This point was defined as starting time of the reaction. After 1 h of reaction time, the reaction was stopped by cooling the reactors using compressed air. Once the reactors reached ambient temperature, samples were collected from all phases (gas, liquid and solid) and analyzed as described below. Further details, including the materials used, can also be found in the Supporting Information (Section Used Chemicals).

### 2.5 Gas Phase Analysis

Gas samples were collected by connecting a gas sampling bag to the reactors. The gas sample was analyzed via gas chromatography (GC) using a Varian GC 450 equipped with a 2 m × 0.75 mm ID ShinCarbon ST column. The bag was compressed for 25 s, and the measurement was started.

### 2.6 Liquid Sample Analysis

The liquid sample was filtered with a 0.20 µm PET injection filter and subsequently analyzed by high‐performance liquid chromatography (HPLC), NMR and Raman spectroscopy. For HPLC analysis, a SHIMADZU NEXERA 40 series HPLC equipped with a polymer phase organic acid column (300 × 8 mm, CHROMATOGRAPHIE‐SERVICE GMBH) was used. The chromatograms were analyzed using the software LabSolutions of Shimadzu. Further detailed information regarding the method can be found in the Supporting Information (Section HPLC Samples).

For NMR analysis, 600 µL of the liquid sample were mixed with 100 µL of deuterium oxide. A BRUKER Advance III HD 400 (AVIII400) NMR spectrometer was used for the analysis. Therefore, ^1^H, ^31^P, ^51^V, and ^1^H spectra with a water suppression method were measured for liquid reaction mixtures. Further detailed information regarding the procedure can be found in the Supporting Information (Section NMR Samples).

The whole volume of the reaction mixture and the catalyst–glucose mixtures before the reactions ( ~10 mL) were filled into a reactor. The Raman probe (RAMAN RXN SYSTEMS of the company KAISER optical systems INC) was immersed in the solution. Each sample was measured for a duration of 3 minutes and an interval of 30 s. Further information can be found in the Supporting Information (Section Raman spectroscopy).

The pH value of each reaction solution was measured in two stages: before and after the reaction. The pH meter employed in this study was a *Lab 850 model* from *SCHOTT Instruments*. Each measurement was taken over a period of 3 min.

### 2.7 Titration

To analyze catalyst acidity via titration, 20 mL of 0.005 M aqueous POM solutions was prepared. These solutions were titrated with 0.2 M Titripur sodium hydroxide solution (LOT: HC43967740; Product Number: 1.09140.1000, by Merck KGaA) using a TitroLine 7800 titration automat from Supporting Information Analytics equipped with a pH A 162 electrode and a TetraCon 925 conductivity electrode. Further details on the method are listed in the Supporting Information (Section Catalyst Screening Titration).

### 2.8 Computational Details for Density functional theory (DFT) Calculations

DFT calculations were performed using the composite r^2^SCAN‐3c functional [47] as implemented in Orca 6.0 [47, 48], with solvation effects included via the conductor‐like polarizable continuum model (C‐PCM) implicit solvent model [49, 50], using water as the solvent. To estimate the pK_a_ of the different protonated states of the Keggin (H_6_PV_3_W_9_O_40_ and H_6_PV_3_Mo_9_O_40_) and Wells‐Dawson (H_9_PV_3_W_15_O_62_ and H_9_P_2_V_3_Mo_15_O_62_) POMs, initially protonated structures were optimized, then a proton was removed (in turn), and the singly deprotonated structure(s) were optimized. This was repeated for the second deprotonation at those sites with the most favorable calculated Δ*G* values (see Supporting Information). This was then repeated up to and including the fourth deprotonation (Keggin) and fifth deprotonation (Wells‐Dawson) until a pK_a_ value substantially higher than the pH at which the POMs are experimentally stable was determined. In all cases, harmonic vibrational frequency analysis was performed to prove that the optimized geometries were minima (i.e., no imaginary frequencies) and to include translational, rotational, and vibrational entropy terms to calculate the Gibbs free energies. The Gibbs free energy of deprotonation at each step was calculated using the general expression:

$$\text{POMH }+\;H_{2}O\to {\mathrm{POM}}^{-}+H_{3}O^{+}$$

$$\text{Δ}G\;=\;[G\left({\mathrm{POM}}^{-}\right)+G(H_{3}O^{+})]-\left[G\left(\text{POMH}\right)+G(H_{2}O)\right]$$

To calculate the energy of the solvated proton, geometry optimizations of H_2_O and H_3_O^+^ were performed at the same level of theory as above (including harmonic vibrational frequency analysis, as described above). The calculated Gibbs free energy change for the solvated proton using this method is −1031.21 kJ mol^−1^.

Estimates of the Lewis acidity of the different (deprotonated) POMs were calculated using the fluoride‐ion affinity (FIA) approach [51, 52, 53]. In this approach, a fluoride ion is attached to centers of interest (in this work, the vanadium atoms, Mo or W) and geometry is optimized. The FIA is then:

$$\mathrm{POM}+F^{-}\to {\mathrm{POMF}}^{-}$$

$$\text{Δ}G\;=\;G\left(\text{POM}\;F^{-}\right)-\left[G\left(\mathrm{POM}\right)+G\left(F^{-}\right)\right]$$

FIA = −ΔG

More positive values of the FIA indicate higher Lewis acidity.

To determine barrier heights of parts of the reaction pathway, transition states were optimized using the same functional as mentioned above. Harmonic vibrational frequency analysis was used to characterize stationary points as transition states (i.e., those with only one imaginary frequency).

### 2.9 Calculations

The evaluation of the HPLC and GC data is conducted through the implementation of specific calculations, which are utilized for the determination of yield, conversion, and selectivity. The substrate conversion (*X*) is calculated using the following Equation (1).

(1) $$X=\frac{n_{\text{substrate},i}-n_{\text{substrate},e}}{n_{\text{substrate},i}}\cdot 100\text{\%}$$

Herein, the molar amount of substrate present after the reaction is deduced from the molar amount of substrate present at the beginning of the reaction. The amount of the substrate is presented by *n*. This value was then divided by the initial amount of substrate.

The following Equation (2) is used to calculate the liquid and gas product yields (*Y*). Therefore, the amount of substance is divided to the initial amount of starting material and, respectively, multiplicated with the number of carbon atoms.

(2) $$\text{Y}\;=\frac{\left(n_{P}\cdot {\;C}_{P}\right)}{\left(n_{\mathrm{Ed}}\cdot {\;C}_{\mathrm{Ed}}\right)}\;\cdot 100\%$$

In this equation, *Y* denotes the yield, and *n* represents the amount of substance. The subscript symbol *P* denotes the products formed, while the symbol *Ed* represents the reactants.

The quantity of substance in the gas phase (*n*_Gas_) is calculated using the following Equation (3). Therefore, the ideal gas law was used and multiplicated with the volume percent from the GC measurement.

(3) $$n_{\mathrm{Gas}}=\frac{P_{e}\;\cdot V_{G}}{R\;\cdot T_{e}}\;\cdot V\%$$

In this model, *P*_e_ denotes the pressure during gas‐phase extraction, while *T*_e_ represents the temperature during the gas sampling. The volume of the gas phase in the reactor is known as *V*_G_ and was calculated by the subtraction of the volume of the reactor and the volume of the liquid phase inside the reactor. *R* is the universal gas constant. The quantity of substance in the gas phase can be calculated using Equation (1). The calculation of the selectivity (*S*) is achieved through the utilization of Equation (4):

(4) $$S\;=\;\frac{Y_{\mathrm{LA}}}{X}\cdot 100\%$$

The selectivity is defined as the yield (*Y*) of LA divided by substrate conversion (*X*).

The standard deviation (*σ*) was calculated by the following Equation (5).

(5) $$\text{σ}=\sqrt{\frac{\sum {\left(x-\bar{x}\right)}^{2}}{\left(n-1\right)}}$$

The equation takes the square root of the sum of the sample *x* substrate from the average of all sample $\bar{x}$. Divided by the number of samples (*n*) minus one.

## 3 Results and Discussion

### 3.1 Identifying Key Parameters in POM Structures for Anaerobic Glucose Transformation to LA

To determine the crucial properties of the POM catalyst responsible for anaerobic glucose conversion into LA, covering its structure and composition, a catalyst screening was carried out (Figure 2) using a systematic selection of POMs. All POMs have been triply substituted with vanadium, as these catalysts have previously demonstrated optimal performance in the studies of Díaz et al. [54], Albert [55], and Raabe et al. [56]. At first, four different structure types were used to find the most promising structure for further modification. The best performing structure types were then modified by changes in the framework metal, substitution of the framework metal, and counterion to verify their influence on the catalytic performance.

**FIGURE 2:**
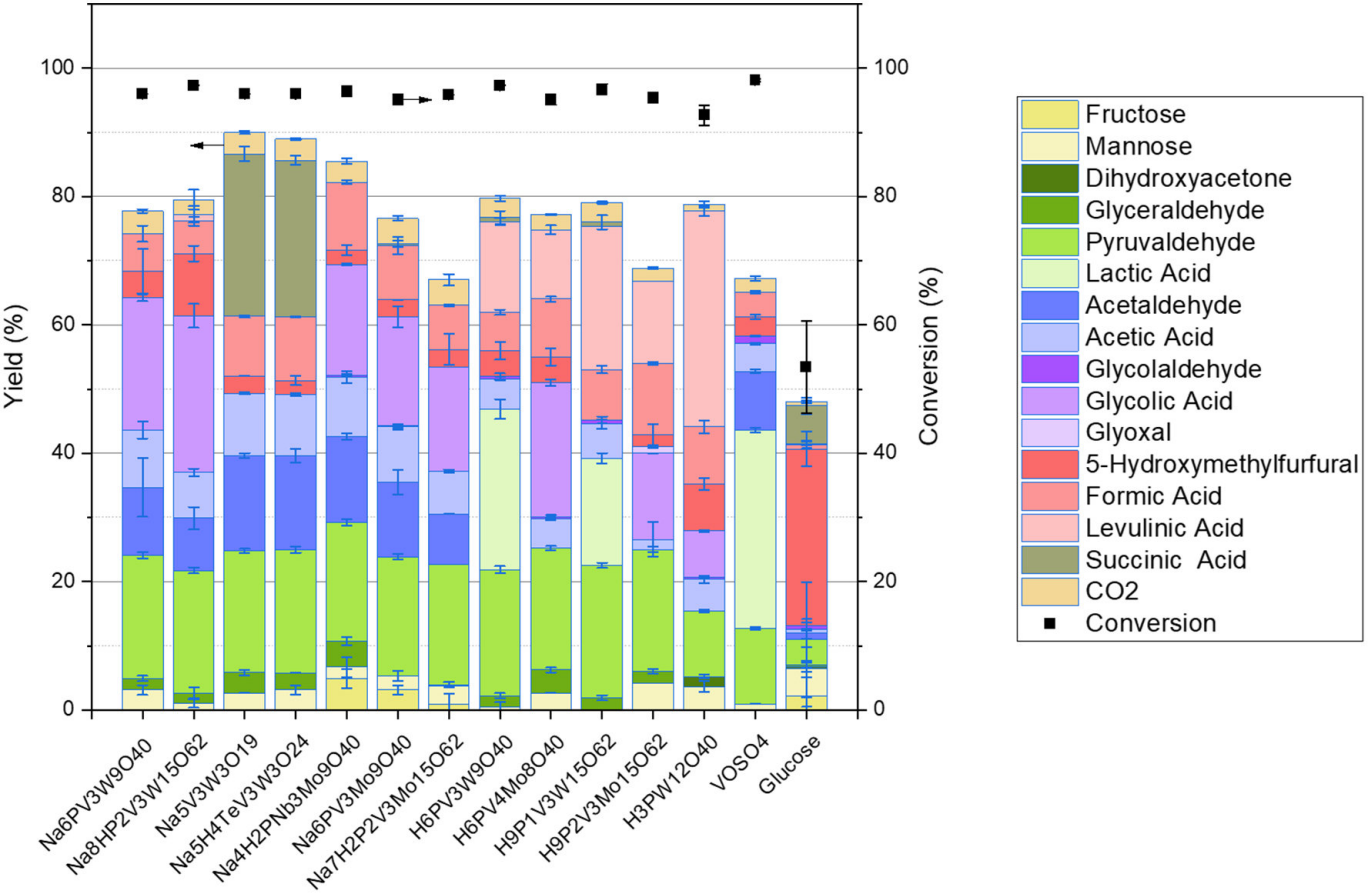
Results of the catalyst screening. Reaction conditions: *n*(Substrate) = 1 mmol glucose; *n*(Catalyst) = 0.1 mmol vanadium; *V* = 10 mL, pN_2_ = 20 bar; *T* = 200 °C; t = 1h; *V*_stirrer_ = level 6; and Solvent: H_2_O.

It was demonstrated that all catalysts exhibited reproducible outcomes (*σ*_blank_ ≤ 9 % *σ*_catalyst_ ≤ 5 %) across the three trials (Tables S2–S22). Glucose conversion increased from 47 % in the blank to approximately 98 % when any catalyst was employed.

The structural POM types Anderson‐Evans (AE‐NaVW), Lindqvist (L‐NaVW), Wells‐Dawson (WD‐NaVW), and Keggin (K‐NaVW) were evaluated with sodium as counterion and tungsten as the framework metal. It is evident from the data presented that none of the sodium salt structures examined yielded LA. The two structure types, L‐NaVW and AE‐NaVW, resulted in a higher yield of succinic acid (25 % and 24 % with a selectivity of 26 % and 25 %, respectively). Succinic acid is formed via the undesired dehydration pathway of glucose to 5‐HMF [24]. Analysis by ^1^H NMR spectroscopy showed a low amount of LA for both catalysts. However, since the signal is below the limit of quantification, the LA yield in these reactions can be considered negligible. On the other hand, K‐NaVW and WD‐NaVW led to a significant formation of GA, showing that the first step (retro‐aldol reaction) in the desired reaction pathway was successfully investigated. Subsequently, the oxidation of GLAD to glycolaldehyde was preferred over the dehydration reaction to pyruvaldehyde. In the following, the Keggin‐type and Wells‐Dawson‐type POMs were further modified, with the aim of following the desired pathway.

To this end, different framework metals, namely, tungsten and molybdenum, were investigated in the K and WD structure types. Changing the framework metal has a significant influence on Brønsted acidity [52, 53, 54, 55, 57, 58, 59] and redox activity [56]. However, the use of diverse framework metals showed no significant differences in catalytic activity for this specific reaction. The conversion of glucose using K‐NaVMo and K‐NaVW was not significantly different. WD‐NaVW showed a slightly higher yield of GA and 5‐HMF than WD‐NaVMo, which can be explained by a higher Brønsted acidity [57, 58, 59] of the tungsten POM compared to that of the molybdenum POM.

Subsequently, the impact of the two substitution metals, vanadium (K‐NaVMo) and niobium (K‐NaNbMo), was examined within the Keggin structure type as those metals showed the highest redox activity [60, 61]. However, the conversion of glucose using niobium as the substituting metal was lower than that using vanadium. The formation of dehydration and oxidation products does not differ significantly between the two substitution metals. When using the unsubstituted catalyst H_3_PW_12_O_40_, no LA was produced, but higher yields of LevA were found. From this, we conclude that the reaction pathway does not proceed in the absence of vanadium or niobium. This was also found when using commercial vanadyl sulfate (VOSO_4_) as a catalyst. VOSO_4_ formed LA with 31 % yield (*S* = 31 %) but also showed a lower yield for all liquid products, which means that more solids were produced. These solids cannot be converted to LA or other products because the carbon is bound in an undefined structure, which is insoluble in aqueous media. Therefore, production of these solids means a loss of carbon during the reaction.

To investigate the influence of countercation variation on the reaction, the sodium ions of Keggin and Wells‐Dawson POMs were partly substituted with protons by employing ion‐exchange resins. The glucose conversion values obtained with the protonated catalysts matched those obtained with the corresponding sodium salts. Regarding the dehydration pathway, the formation of LevA was enhanced in all protonated POMs, while the yield of 5‐HMF was reduced (see Figure 1). This can be related to the conversion of 5‐HMF into LevA by an acid‐catalyzed reaction [21, 25, 26, 27, 28, 29, 30, 38]. The yield of FA increased for the same reasons. Interestingly, the decarboxylation of LA to AcetAl was reduced by protonation. The combination of tungsten and protons appeared to be a prerequisite for the effective conversion of glucose into LA. The highest yield, which was approximately 25 % (*S* = 26 %), was achieved using K‐HVW (see Tables S3 and S4; S6 and S7; S9 and S10), while WD‐HVW achieved 17 % LA (*S* = 17 %). In summary, these findings demonstrate that the combination of tungsten as framework element, partially substituted by vanadium and protons as counterions, is essential for the formation of LA from glucose. Furthermore, the Keggin‐type structure seems superior compared to other structure types. With the compositional and structural key parameters identified, the next step in this study was to investigate the molecular properties such as acidity and redox activity to understand the influence of these key parameters on the catalytic performance.

**TABLE 1:** Calculated pK_a_ and pK_a_ derived from titration data. DFT calculated values are taken from the third deprotonation step (see Tables S40 and S41).

Catalyst	EQ/mL_NaOH_	pH at EQ	HEQ/ml_NaOH_	pK_a_ (titration)	pK_a_ (DFT)
K‐HVMo	3.45	6.13	1.70	2.57	2.73
K‐HVW	2.80	4.77	1.40	2.09	0.90
WD‐HVMo	0.86	4.16	0.45	2.32	2.71
WD‐HVW	4.53	6.24	2.25	1.81	−0.89

### 3.2 Investigation of Acidity and Redox Potential for the Most Promising K‐HVW Catalyst

To get a deeper understanding of how the framework metal and the counterion, especially the combination of tungsten and protons, influence the reaction, a titration and electrochemical analysis of the protonated catalysts were conducted. The results were validated by DFT calculations.

Titrations of aqueous solutions of the protonated POMs were performed to determine the influence of catalyst acidity on the respective product scope (see Table 1 and Figure S15). Since POMs in their protonated form are known to be highly acidic [62, 63], it is safe to assume that the equivalence points (EQ) observed in these titrations belong to the second, third, or even higher proton dissociation. Based on these titration graphs, pK_a_ values were estimated for each POM, assuming that the deprotonation step in question is similar to that of a weak Brønsted acid. After determining the EQ and the half‐EQ (HEQ), pH at HEQ is equal to the estimated pK_a_. It was found that WD‐HVW is the most acidic catalyst (1.81), followed by the tungsten‐containing Keggin catalyst K‐HVW (2.09). Both molybdenum‐containing POMs are slightly less acidic with the WD‐HVMo (2.32) ranked third and K‐HVMo (2.57) as least acidic POM.

In order to overcome the challenge of assigning the measured pK_a_ values to specific deprotonation steps, the experimental determination was complemented by DFT calculations. The pK_a_ values derived from the DFT calculations are given in Tables S40 and S41 for successive deprotonation steps. The data shows that all the POMs are highly acidic with pK_a_ values <0 for the first and second deprotonation step. The pK_a_ value at the third deprotonation step shows the best agreement with the experimentally derived values. At the fourth deprotonation step, both K‐HVMo and K‐HVW have very high pK_a_ values (12.4 and 10.2, respectively) lying outside of the pH range in which the POMs are expected to be stable [64]. This indicates that the triply deprotonated species is likely dominant in aqueous solution under the applied reaction conditions. This is also true for WD‐HVMo (pK_a_ = 6.1 at the fourth deprotonation step), while WD‐HVW has a pK_a_ of 3.5 at the fourth deprotonation step, suggesting that this POM may have been deprotonated either three or four times in aqueous solution. In agreement with the experimental observations above, WD‐HVW is the most acidic, followed by K‐HVW, WD‐HVMo, and K‐HVMo.

FIA values calculated by DFT are given in Table 2. Where the fluoride ion is bound at the vanadium (V) sites, the FIA values indicate weak Lewis acids, which is also indicated in the relatively weak binding motif as seen in Figure 3. The fluoride ion associates weakly to the area containing V atom(s). The FIA values when the fluoride is bound at either Mo or W (except for K‐HVMo) indicate very weak Lewis acids. These data indicate that the POMs are simultaneously strong Brønsted acids and weak Lewis acids.

**TABLE 2:** Calculated fluoride ion affinity (FIA) values, in kJ mol^−1^. The element symbols V, Mo, and W indicate where the fluoride ion is binding. More positive FIA values indicate stronger Lewis acidity.

Catalyst	FIA (V)	FIA (Mo)	FIA (W)
K‐HVMo	1.9	5.5	—
K‐HVW	−6.5	—	−97.2
WD‐HVMo	8.4	−23.4	—
WD‐HVW	−3.3	—	−78.8

**FIGURE 3:**
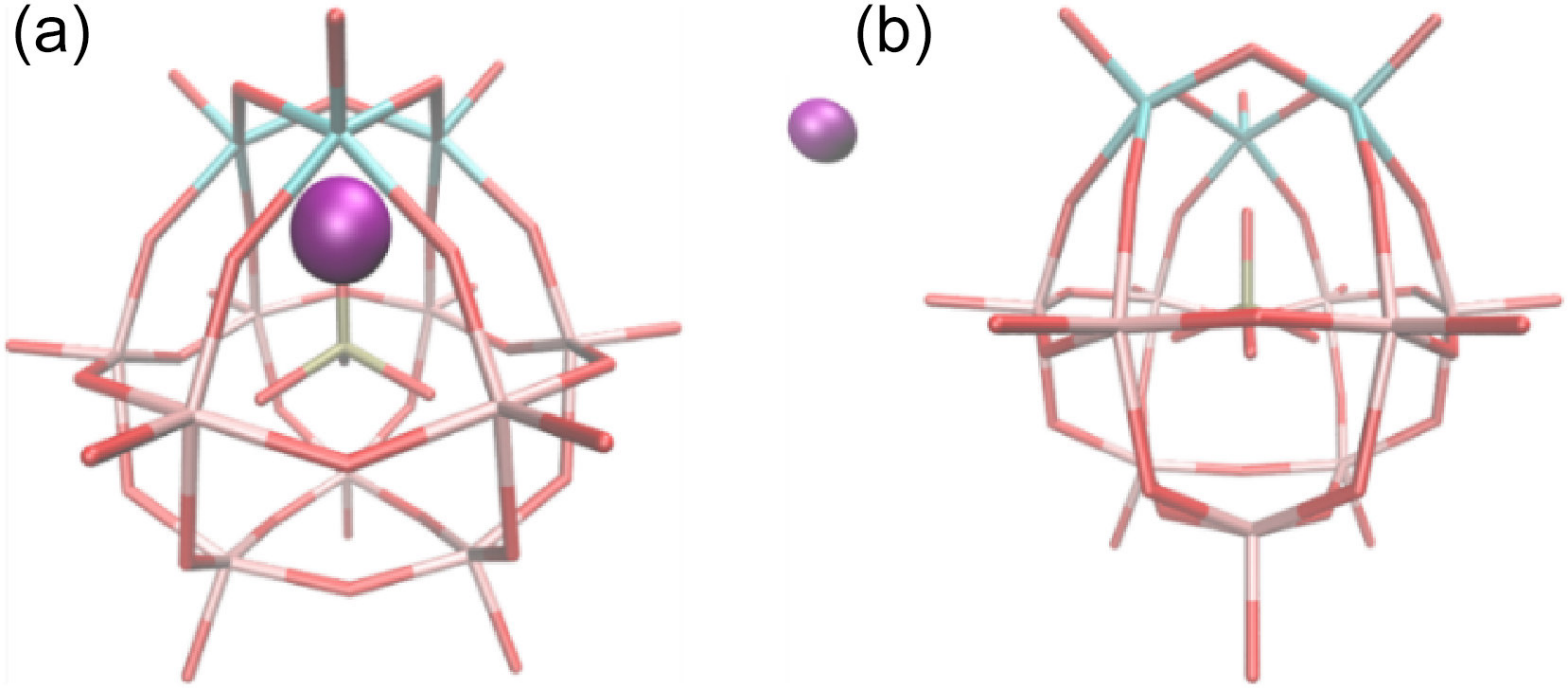
3D view of the binding motif of F^−^ to K‐HVMo, (a) front and (b) side view. Fluoride ion (purple); vanadium atoms (cyan); oxygen atoms (red); and molybdenum atoms (light pink).

Overall, these investigations support the hypothesis that catalyst acidity plays a key role in LA production, as only the two most acidic POMs investigated lead to LA formation. Most notably, the best catalyst (K‐HVW) exhibits the lowest FIA, which indicates that high Brønsted acidity is more relevant for catalytic performance in this reaction than Lewis acidity.

To further elucidate the individual parameters governing the catalytic activity of POMs toward LA formation, cyclic voltammetry (CV, Figures S16–S23) and square wave voltammetry (SWV, Figures S24–S25) were employed. CV measurements were recorded at varying scan rates to assess the redox stability of the Keggin‐ and Wells‐Dawson‐type POMs across the full accessible potential window in acidic solution (pH 1). In general, the tungsten‐based POMs exhibit more well‐defined redox waves and a significantly higher degree of reversibility, as indicated by the comparatively small peak‐to‐peak separation and the nearly equal anodic and cathodic peak currents in the cyclic voltammograms, even at low scan rates. As the first reduction of the POM corresponds to oxidation of the substrate and is therefore most relevant to catalytic turnover, we examined this process in more detail by SWV (Figure 4). A pronounced difference in the reduction potential is observed between K‐HVMo and K‐HVW: the tungsten derivative displays a significantly more positive reduction potential, with two closely spaced redox events (0.403 V vs. Ag^0/+^ and 0.608 V vs. Ag^0/+^), whereas all molybdenum analogs exhibit notable potential compression with the first redox event between 0.472 V versus Ag^0/+^ (K‐HVMo) and 0.483 V versus Ag^0/+^ (WD‐NaVMo), independent of the POM‐type. In the case of the sodium salt K‐NaVW, a considerably diminished redox response was observed, which may in part be attributed to the lower conductivity of Na^+^ compared to that of H^+^. Taken together, these findings indicate that an intricate interplay between Brønsted acidity and redox potential governs the ability of given POMs to selectively promote LA formation while suppressing competing side reactions.

**FIGURE 4:**
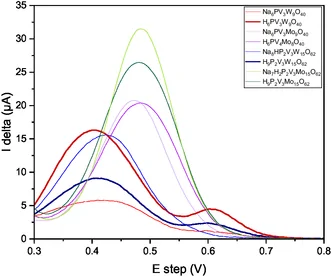
SWV investigation of the first redox event. Concentration: 1 mmol/L, Scan rate: 5 mv/s, and pH 1 (Hydrochloric acid was used as supporting electrolyte). Data were smoothed using fast Fourier transformation to eliminate high‐frequency noise.

### 3.3 Evaluating the Influence of the Oxidation State of Vanadium for Anaerobic Glucose Conversion to LA

During the reaction involving glucose conversion, the vanadium species of the catalyst K‐HVW was reduced from vanadium (V) to vanadium (IV), which is confirmed by our DFT calculations, showing nearly equal spin densities of ~0.63–0.69 (using the Löwdin population analysis, where a value of 1 is a single unpaired electron) at each of the vanadium centers in reduced K‐HVW. In a previous study, Tang et al. [28] used vanadyl sulfate (VOSO_4_), in which the redox state is V(IV), as a commercial catalyst and received 56 % yield of LA from glucose. A lower yield was found at 200 °C in the catalyst‐screening (*Y* = 31 %). As such, we investigated the influence of the redox state of the catalyst by reducing the K‐HVW catalyst using hydrazine (see Section 2 for further details) and using the resulting reduced catalyst. The result of the comparison between the oxidized and reduced catalysts is shown in Figure 5.

**FIGURE 5:**
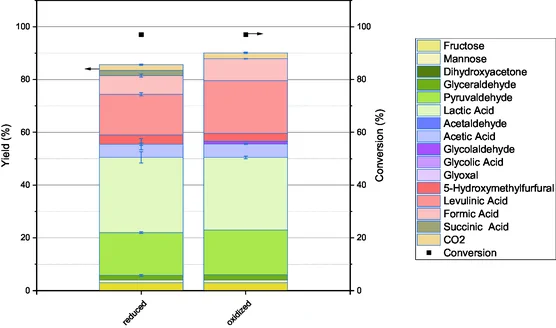
Results of the study for the oxidized vs the reduced catalyst (Cat: H_6_PV_3_W_9_O_40_). Reaction conditions: *n*(Substrate) = 1 mmol glucose; *n*(Catalyst) = 0.1 mmol vanadium; *V* = 10 mL, pN_2_ = 20 bar; *T* = 200 °C; *t* = 1 h; *V*_stirrer_ = level 6; and Solvent: H_2_O.

The difference in the conversion for both experiments was not significant, with 85% for the reduced and 90% for the oxidized form of K‐HVW (Tables S37–S39). Additionally, no difference in LA yield was found. However, there was a significant increase in the yield of the dehydration pathway product 5‐HMF from 1% to 9%. The yield of LevA decreased slightly from 16% using the oxidized catalyst compared to 13% with the reduced catalyst. In summary, the yield of the dehydration pathway products decreased when using the reduced catalyst. However, the carbon balance also decreased, so higher yields of solids were formed. This is related to a loss of carbon for the valorization to LA. Consequently, the oxidized K‐HVW species were used for further experiments.

### 3.4 Stability of the Catalyst

The stability of K‐HVW was verified by Raman spectroscopy, as shown in Figure 6.

**FIGURE 6:**
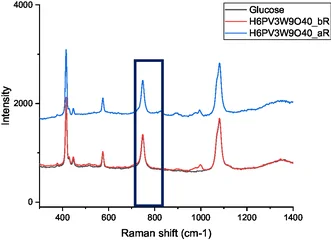
Raman spectroscopy measurement of H_6_PV_3_W_9_O_40_. bR: before reaction. aR: after reaction. All solutions were measured in water as solvent. Reaction conditions: *n*(Substrate) = 1 mmol glucose; *n*(Catalyst) = 0.1 mmol vanadium; *V* = 10 mL, pN_2_ = 20 bar; *T* =  200 °C; *t* = 1 h; *V*_stirrer_ = level 6; and Solvent: H_2_O.

As illustrated in Figure 6 the Raman spectroscopy measurement of the glucose solution without catalyst (black lines) revealed the presence of multiple signals consistent with the findings reported in the literature [65]. In comparison to the measurement of the glucose solution with catalyst before reaction (red line), a significant number of peaks are found to be overlapped. One signal at 800 cm^−1^ [56, 65] identifies the K‐HVW catalyst in the measurement. This signal is also discernible in the measurement of the reaction mixture with catalyst after thereaction (blue line). This demonstrates the stability of the catalyst during the reaction.format="sup"

### 3.5 Investigations of Reaction Pathway and Stability of Intermediates

The reaction pathway from glucose to LA (Figure 1) has several potential selectivity‐determining steps (SDS). These are critical points in the reaction pathway at which two reactions compete with each other, leading to different species. The first possible SDS is the retro‐aldol reaction of glucose/fructose to DHA/GLAD competing with the dehydration reaction to 5‐HMF. The second potential SDS is the reaction of GLAD to pyruvaldehyde via dehydration or oxidation to glycolaldehyde. In order to identify the relevant SDS for the formation of LA, the different reaction intermediates and the product (fructose, DHA, GLAD, pyruvaldehyde, and LA) were employed as starting materials (Tables S23–S36). The outcome of each reaction with and without the POM catalyst was compared for the different intermediates and the desired product (Figure 7), to identify the main pathways and critical SDS.

**FIGURE 7:**
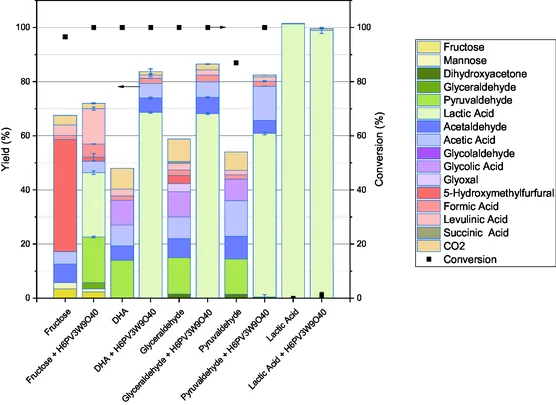
Conversion of reaction intermediates and the influence of the catalyst (Cat: H_6_PV_3_W_9_O_40_) on the reaction pathway. Reaction conditions: *n*(Substrate) = 1 mmol (C6‐substrates), 1.5 mmol (C4‐substrates), 2 mmol (C3‐substrates); *n*(Catalyst) = 0.1 mmol vanadium; *V* = 10 mL, pN_2_ = 20 bar; *T* = 200 °C; *t* = 1 h; *V*_stirrer_ = level 6; and Solvent: H_2_O.

The experiments show the influence of the catalyst on the reaction pathway by comparing the thermal decomposition with catalytic transformations. Figure 7 shows that using K‐HVW enhances the conversion of fructose and pyruvaldehyde, compared to the catalyst screening, and the formation of LA from all substrates, namely fructose, DHA, GLAD, and pyruvaldehyde up to 24 %, 69 %, 68 %, and 60 %, respectively. In the absence of the catalyst, the production of LA was zero from all substrates. The first SDS was evaluated using fructose. Without using the catalyst, dehydration to 5‐HMF was preferred. By using the catalyst, the retro‐aldol reaction was enabled, causing higher yields of pyruvaldehyde (17 %) and LA (24 %) instead of dehydration products like 5‐HMF (2 %), FA (5 %), and LevA (13 %).

In the following, DHA and GLAD were used as substrates. Here, formation of GA, the oxidation product of GLAD, was the preferred pathway without catalyst (~10 %). By using K‐HVW, the production of LA increased for both DHA and GLAD (up to 69 % and 68 %, respectively). From using fructose as the substrate to DHA/GLAD as substrates, the yield of LA was significantly increased. As such, the retro‐aldol reaction can be evaluated as the critical SDS with a high influence on the reaction pathway. For the next step, pyruvaldehyde was used as a substrate. The results for the blank experiment were similar to those of DHA and GLAD. The yield of LA decreased to 60 % in favor of a higher yield of acetic acid. However, the small differences between the results of pyruvaldehyde and DHA/GLAD showed that this step is not relevant for the overall selectivity of the reaction. However, the consecutive product AcetAl was found by using DHA, GLAD, and pyruvaldehyde. It can be postulated that AcetAl is not a product of the consecutive reaction of LA but rather a consecutive product of pyruvaldehyde, which is a new discovery in this reaction network. While the computed barrier to the reaction of this transformation (in the absence of the catalyst) is ~200 kJ mol^−1^, with K‐HVW present, the barrier reduces to 130 kJ mol^−1^. Proton transfer to and from K‐HVW is key to this reduction in barrier height. At the temperatures used in the reaction process, the Eyring equation gives a half‐life of the reaction of 4.5 h, revealing this reaction (producing AcetAl and carbon monoxide) to be competitive with the rehydration reaction of pyruvaldehyde. The modified reaction network, including this new finding, is shown in Figure 8. The decarboxylation of pyruvaldehyde to AcetAl can be followed by an oxidation reaction to acetic acid which has a higher yield by using pyruvaldehyde as a substrate. The yields for acetic acid and AcetAl were not affected by using the catalyst. Therefore, we can conclude that this step has an effect on the reaction but cannot be influenced in the current system. A decrease of the carbon dioxide yield was observed by using K‐HVW. By using LA as a substrate, there were no significant differences between the blank and the use of K‐HVW. LA seemed to be stable under the chosen reaction conditions.

**FIGURE 8:**
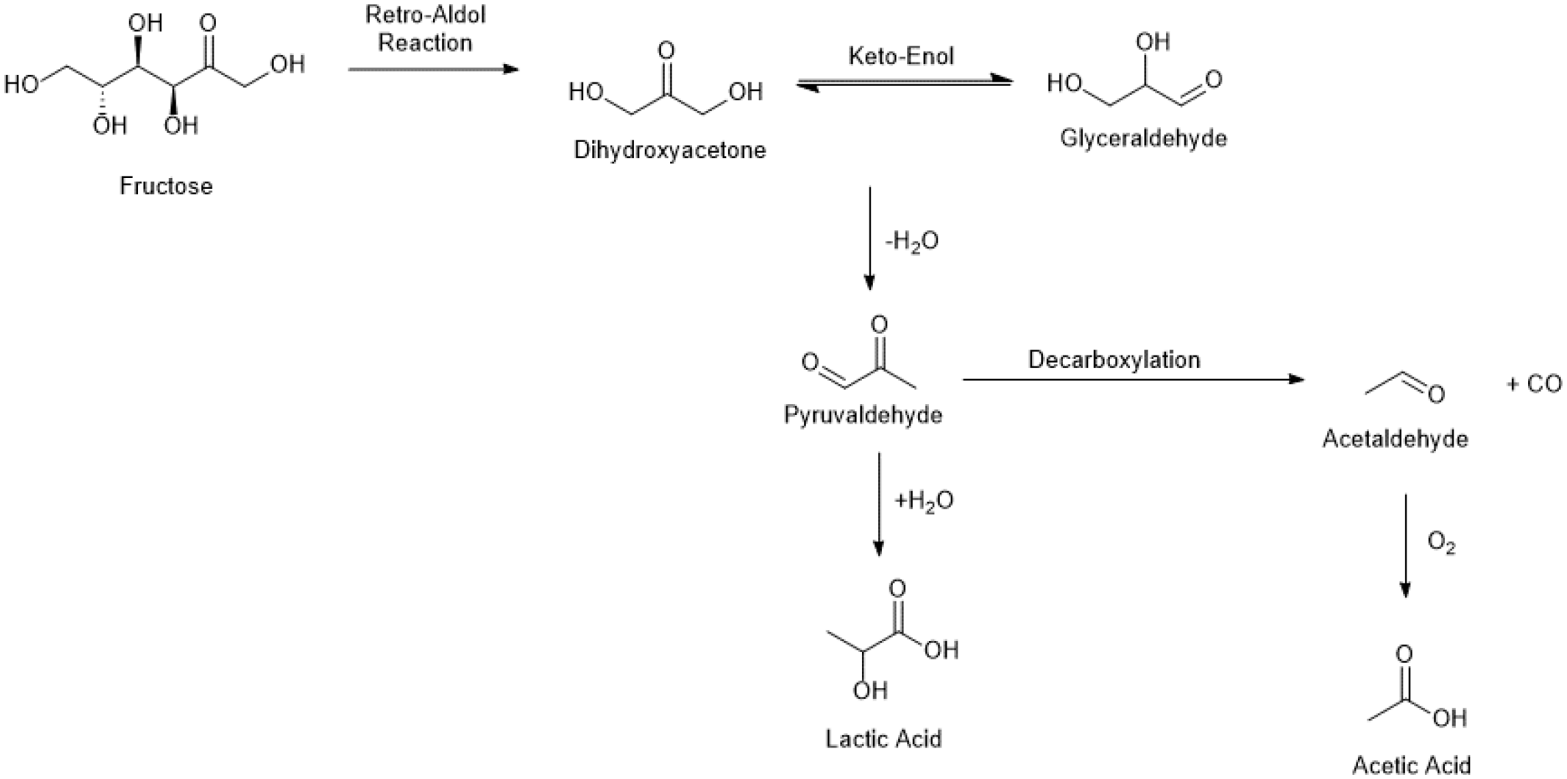
Alternative reaction network deduced from mechanistic investigations. The conversion of glucose to LA, accompanied by the formation of AcetAl, a product resulting from the transformation of pyruvaldehyde.

To sum up, the retro‐aldol reaction is the critical SDS for this reaction. The dehydration to pyruvaldehyde is not critical. The newly discovered side reaction of the decarboxylation of pyruvaldehyde cannot be affected by using K‐HVW.

## 4 Conclusion

The present study investigated the key parameters of polyoxometalate catalysts for the conversion of glucose to LA under anaerobic conditions. Therefore, a range of catalysts was investigated, incorporating diverse structural types, substitution metals, framework metals, and counterions. It was found that merely two catalysts were capable of producing LA as the desired product. The H_9_PV_3_W_15_O_62_ catalyst exhibited an LA yield of 17 %. The H_6_PV_3_W_9_O_40_ catalyst demonstrated the highest level of performance in the screening process, with an LA yield of 25 %. All other catalysts resulted in the formation of GA as the primary product, which is an oxidative pathway product of GLAD. In order to understand which molecular characteristics are responsible for this outcome, we investigated the acidity experimentally and with DFT calculations and the redox activity with electrochemical measurements. We found that the best‐performing catalyst H_6_PV_3_W_9_O_40_ exhibits the highest Brønsted acidity combined with the lowest Lewis acidity among the tested catalysts and exhibits unique redox behavior.

The use of a reduced catalyst in comparison to the oxidized catalyst showed no differences in the LA yield but resulted in a lower amount of dehydration products. There is no positive influence of using the reduced catalyst, as claimed by previous studies [28].

In the subsequent stage of the investigation, the reaction pathway was examined using the most effective catalyst (H‐KVW). The results demonstrated that the utilization of the catalyst facilitated the formation of LA. It has been demonstrated that LA is stable when K‐HVW is used as the catalyst under the applied reaction conditions. In the absence of the catalyst, lower yields of both the dehydration product and GA were observed. Further investigations demonstrated that AcetAl is not a byproduct of the decarboxylation of LA but rather a decarboxylation product of pyruvaldehyde.

In summary, a polyoxometalate catalyst that exhibited high performance in the valorization of glucose to LA (*Y* = 25 %, *S* = 26 %) under anaerobic conditions was identified. Acidity and redox activity are key parameters for the effective use of POMs for this reaction. The results are an important step toward the efficient production of sustainable platform chemicals like LA using POMs as homogeneous catalysts.

## Funding

This study was supported by European Union (ERC, BioValCat, Project 101086573). Views and opinions expressed are however, those of the author(s) only and do not necessarily reflect those of the European Union or the European Research Council. Neither the European Union nor the granting authority can be held responsible for them. For the purposes of open access, the authors have applied a Creative Commons Attribution (CC BY) license to any Author Accepted Manuscript version arising from this submission.

## Conflicts of Interest

The authors declare no conflicts of interest.

## Supporting information

Used materials; details of catalyst synthesis; in‐depth catalyst characterization, used equipment; and detailed procedures for sample evaluation can be found in the Supporting Information file.

## Data Availability

Research data is available after official publication (10.5281/zenodo.20343552).
