# Supplementary material for: Identifying Key Features of Polyoxometalate Catalysts for the Anaerobic Conversion of Glucose Into Lactic Acid
Source: ChemSusChem. 2026 Nov 10;19(22):e71192. doi: 10.1002/cssc.71192 (PMC13752457; doi:10.1002/cssc.71192)
Supplement: Supplementary file 1 [file CSSC-19-e71192-s001.pdf]

Supporting Information for

Identifying Key Features of Polyoxometalate Catalysts for the Anaerobic Conversion of Glucose into Lactic Acid

Elisabeth Hundt[a], Pegah Saedi[a], Lasse Prawitt[a], Andreas H. Pawlig[a], Jan-Dominik H. Krueger[a], Maximilian J. Poller[a], David Robinson[b], Jakob Albert[a*]

[a] Institute of Technical and Macromolecular Chemistry, Universität Hamburg, Bundesstraße 45, 20146 Hamburg, Germany

[b] Department of Physical Sciences, School of Science and Technology, Nottingham Trent University, Clifton Lane, Nottingham, NG11 8NS United Kingdom

Contact details of the corresponding author: jakob.albert@uni-hamburg.de

Table of Contents

Catalyst Synthesis ... 3

Catalyst Characterization ... 4

K-NaVW ($Na_6PV_3W_9O_{40}$) ... 4

K-HVW ($H_6PV_3W_9O_{40}$) ... 5

K-NaVMo ($Na_6PV_3Mo_9O_{40}$) ... 6

K-HVMo ($H_6PV_3Mo_9O_{40}$) ... 7

K-NaNbMo ($Na_4H_2PNb_3Mo_9O_{40}$) ... 8

WD-NaVW ($Na_8HP_2V_3W_{15}O_{62}$) ... 9

WD-HVW ($H_9PV_3W_{15}O_{62}$) ... 10

WD-NaVMo ($Na_7H_2P_2V_3Mo_{15}O_{62}$) ... 11

WD-HVMo ($H_9P_2V_3Mo_{15}O_{62}$) ... 12

L-NaVW ($Na_5V_3W_3O_{19}$) ... 13

AE-NaVW ($Na_5H_4TeV_3W_3O_{24}$) ... 13

Reduced K-HVW ($H_6PV_3W_9O_{40}$) ... 14

IR Spectra of all Catalysts ... 15

Flow chart 10-fold plant ... 16

Sample preparation for downstream processing ... 17

Used Chemicals ... 17

Details of analytical procedures ... 17

Solid sample analysis ... 17

Gas samples ... 17

HPLC Samples ... 17

NMR Samples ... 17

Raman spectroscopy ... 17

Catalyst Screening ... 18

First run 18
Second run 19
Third run 21
Replication of WD-NaVW 22
Commercial catalyst $VOSO_4$ 23
Unsubstituted Catalyst $H_3PW_{12}O_{40}$ 26
Titration 28
Electrochemistry 29
Pathway Investigations 34
Fructose 34
Dihydroxyacetone 35
Glyceraldehyde 37
Pyruvaldehyde 38
Lactic Acid 40
Oxidation state of the catalyst 41
DFT calculated pK_a values 43

Catalyst Synthesis

The synthesis of all sodium salt catalysts was performed as described in the literature by Pawlig *et al*. [1] and Wesner *et al*. [2]. To exchange the countercation, an ion-exchange was performed using a strongly acidic ion exchange resin (DuPont™AmberLite™ HPR650 H). For this, the catalyst was dissolved in water and residue was filtered off. The ion-exchange column was conditioned with 300 ml of a 7 % HCl solution for 30 min. After this, the column was neutralized using deionized water. Subsequently, the catalyst-solution was eluted with a speed of 8 ml min^{-1}. After that, the rest of the catalyst was washed out of the column with deionized water. The aqueous fractions were pooled and concentrated under reduced pressure and 80 °C via rotary evaporation for 15 – 20 h to afford the desired products.

Catalyst Characterization

K-NaVW ($Na_6PV_3W_9O_{40}$)

^{31}P-NMR (161.98 MHz, H_2O/D_2O, 26 °C): δ [ppm] = -12.8

^{51}V-NMR (105.18 MHz, H_2O/D_2O, 26 °C): δ [ppm] = -493.3, -497.6, -501.8, -525.8, -529.0, -543.1, -549.2, -551.6, -553.53, -556.1, -557.28

IR (ATR): ṽ [cm^{-1}] = 1616 (O-H, lattice H_2O), 1098, 1074, 1053 (w, P-O), 947 ((M=O)t), 866 ((M-O-M)vertex), 746 ((M-O-M)edge)

ICP-OES: Calculated for $Na_6PV_3W_9O_{40}$ x 8 H_2O: 1.18 % P, 5.84 % V, 63.23 % W. Found for $Na_6PV_3W_9O_{40}$ x 8 H_2O: 1.20 % P, 5.97 % V, 55.13 % W, 7.01% Na. Normalized elemental ratio (V + W = 12): Na/P/V/W ratio: 8.77/1.12/3.37/8.63

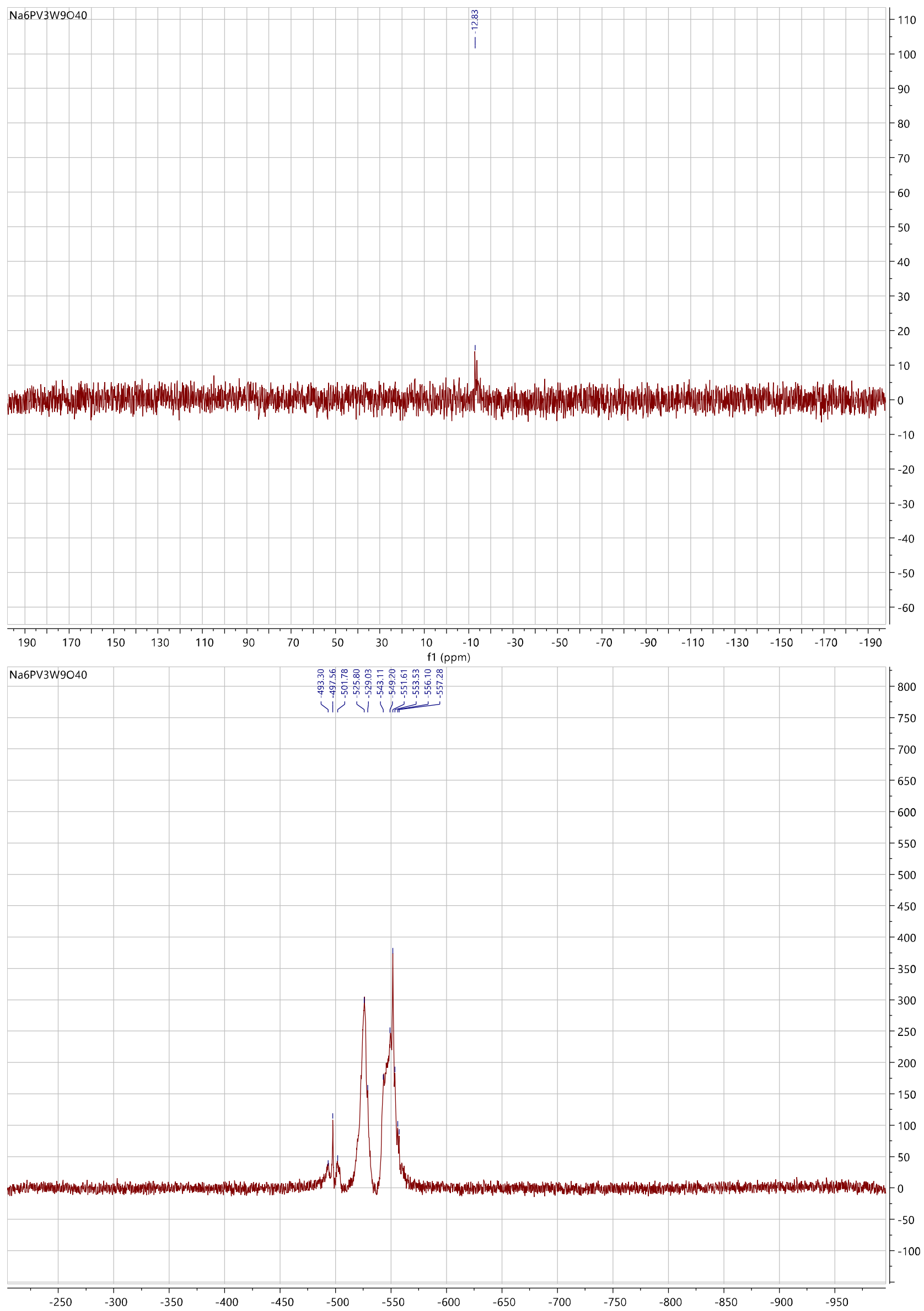

Figure S1: ^{31}P- (top figure) & ^{51}V- NMR (bottom figure) of the $Na_6PV_3W_9O_{40}$ catalyst.

K-HVW ($H_6PV_3W_9O_{40}$)

^{31}P-NMR (161.98 MHz, H_2O/D_2O, 26 °C): δ [ppm] = -14.14, -14.91

^{51}V-NMR (105.18 MHz, H_2O/D_2O, 26 °C): δ [ppm] = -526.63, -529.21, -542.56, -546.82, -551.36, -552.63, -553.27, -553.92, -555.05, -555.52, -557.41, -559.43

IR (ATR): ṽ [cm^{-1}] = 1607 (O-H, lattice H_2O), 1059 (w, P-O), 961 ((M=O)t), 881 ((M-O-M)vertex), 723 ((M-O-M)edge).

ICP-OES: Calculated for $H_6PV_3W_9O_{40}$ x 10 H_2O: 1.25 % P, 6.15 % V, 66.60% W. Found for $H_6PV_3W_9O_{40}$ x 10 H_2O: 1.19 % P, 6.42 % V, 58.1 % W, 0.08 % Na. Normalized elemental ratio (V + W = 12): Na/P/V/W ratio: 0.1/1.04/3.42/8.58

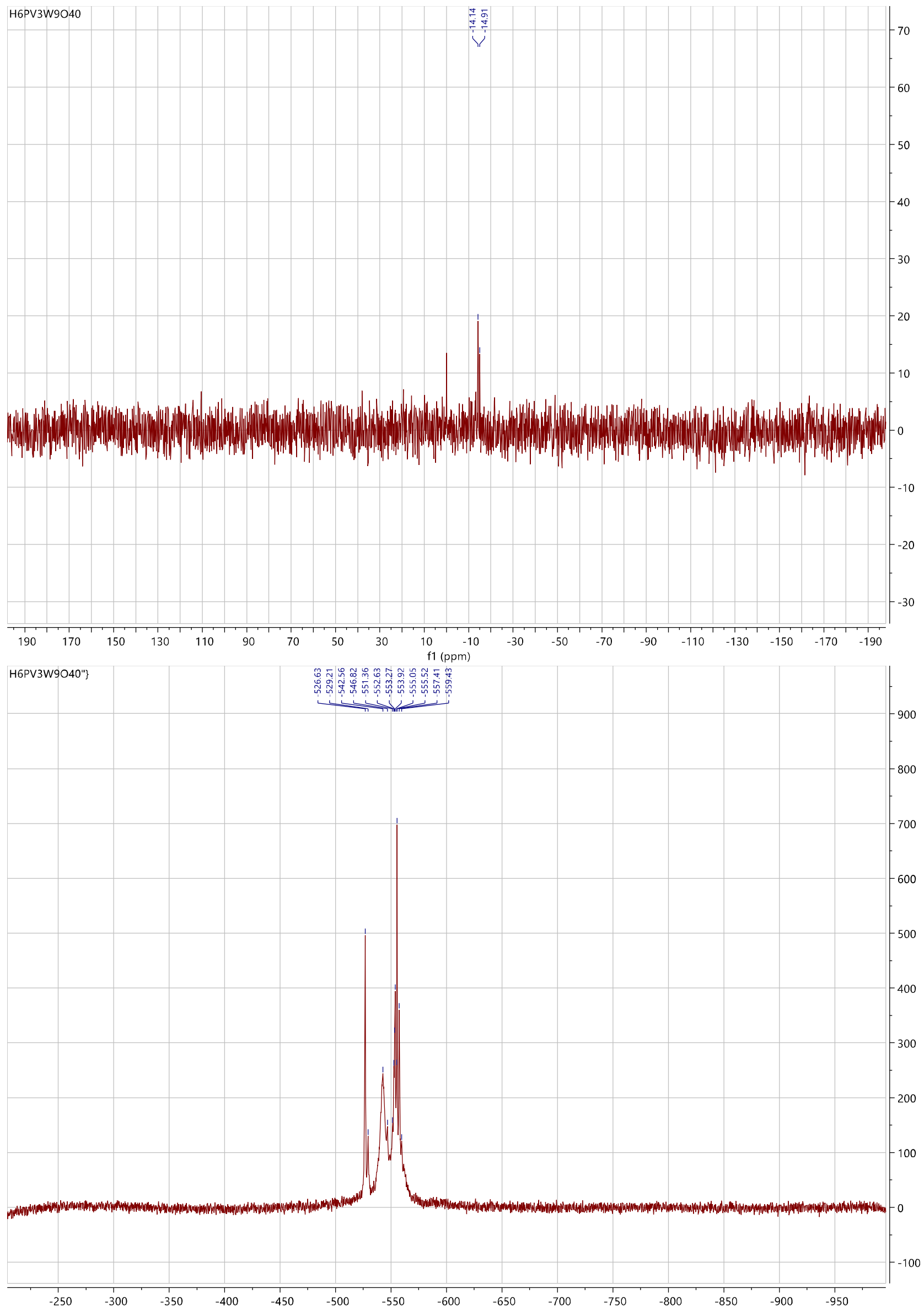

Figure S2: ^{31}P- (top figure) & ^{51}V- NMR (bottom figure) of the $H_6PV_3W_9O_{40}$ catalyst.

K-NaVMo ($Na_6PV_3Mo_9O_{40}$)

^{31}P-NMR (161.98 MHz, H_2O/D_2O, 26 °C): δ [ppm] = -4.12

^{51}V-NMR (105.18 MHz, H_2O/D_2O, 26 °C): δ [ppm] = -513.30, -515.98, -519.29, -531.38, -532.06, -533.13, -533.97, -536.19

IR (ATR): ṽ [cm^{-1}] = 1617 (O-H, lattice H_2O), 1064, 1048 (w, P-O), 950, 937 ((M=O)t), 845 ((M-O-M)vertex), 758 ((M-O-M)edge)

ICP-OES: Calculated for $Na_6PV_3Mo_9O_{40}$ x 7 H_2O: 1.70 % P, 8.37 % V, 47.31 % Mo. Found for $Na_6PV_3Mo_9O_{40}$ x 7 H_2O: 1.47 % P, 6.72 % V, 40.8 % Mo. Normalized elemental ratio (V + W = 12): Na/P/V/Mo ratio: 6.42/1.03/2.84/9.16

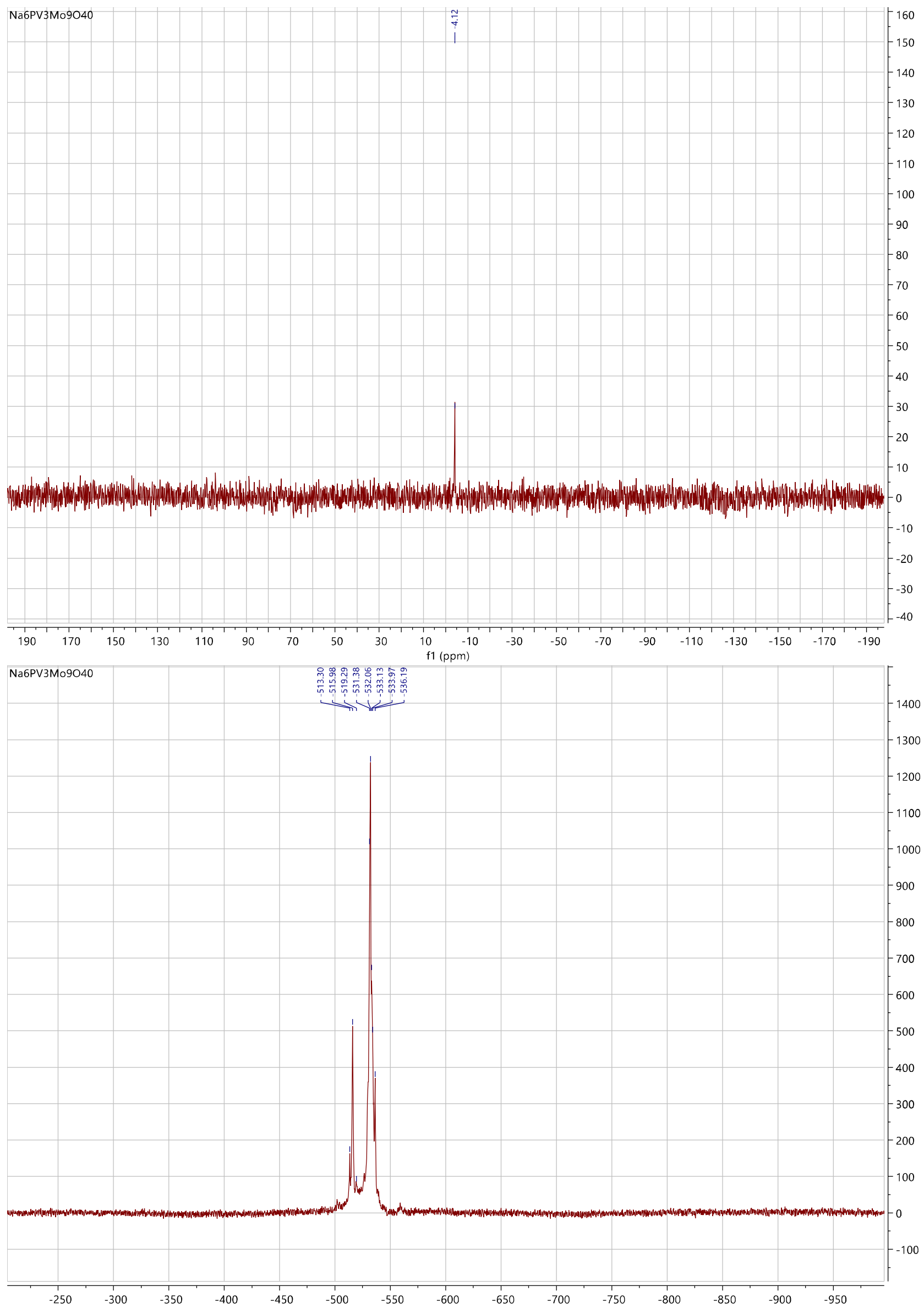

Figure S3: ^{31}P- (top figure) & ^{51}V- NMR (bottom figure) of the $Na_6PV_3Mo_9O_{40}$ catalyst.

K-HVMo ($H_6PV_3Mo_9O_{40}$)

^{31}P-NMR (242.9 MHz, H_2O/D_2O, 25 °C): δ [ppm] = -3.50, -3.64, -3.70, -4.25

^{51}V-NMR (157.8 MHz, H_2O/D_2O, 25 °C): δ [ppm] = -519.03, -531.15, -532.56, -533.02, -534.28, -535.99, -540.15, -541.59, -546.54

IR (ATR): ṽ [cm^{-1}] = 1053 (w, P-O), 952 ((M=O)t), 873 ((M-O-M)vertex), 747 ((M-O-M)edge)

ICP-OES: Calculated for $H_6PV_3Mo_9O_{40}$ x 7 H_2O: 1.83 % P, 9.04 % V, 51.00 % Mo. Found for $H_6PV_3Mo_9O_{40}$ x 7 H_2O: 2.20 % P, 9.59 % V, 43.7 % Mo. Normalized elemental ratio (V + W = 12): Na/P/V/Mo ratio: 0/1.32/3.51/8.49

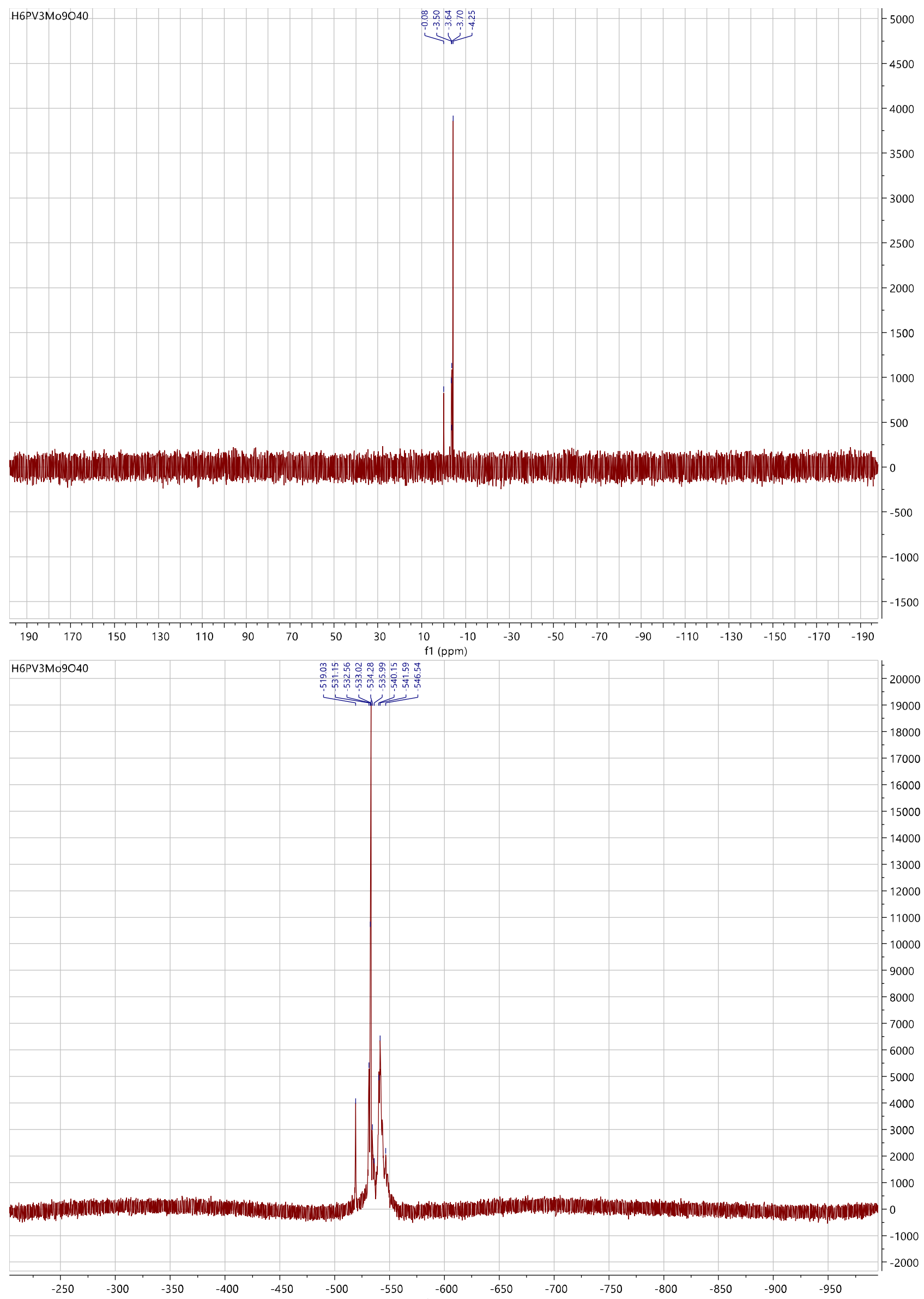

Figure S4: ^{31}P- (top figure) & ^{51}V- NMR (bottom figure) of the $H_6PV_3Mo_9O_{40}$ catalyst.

K-NaNbMo ($Na_4H_2PNb_3Mo_9O_{40}$)

^{31}P-NMR (242.9 MHz, H_2O/D_2O, 25 °C): δ [ppm] = -2.36, -2.48, -2.52, -2.61, -2.74, -2.78, -2.95, -3.09, -3.18, -3.27, -3.32, -3.37

IR (ATR): ṽ [cm^{-1}] = 1613 (O-H, lattice H_2O), 1047 (w, P-O), 944 ((M=O)t), 857 ((M-O-M)vertex), 759 ((M-O-M)edge)

ICP-OES: Calculated for $Na_6PNb_3Mo_9O_{40}$ x 7 H_2O: 1.70 % P, 8.37 % V, 47.31 % Mo. Found for $Na_6PNb_3Mo_9O_{40}$ x 7 H_2O: 1.44 % P, 14.23 % Nb, 39.4 % Mo. Normalized elemental ratio (V + W = 12): Na/P/Nb/Mo ratio: 4.01/0.99/3.26/8.74

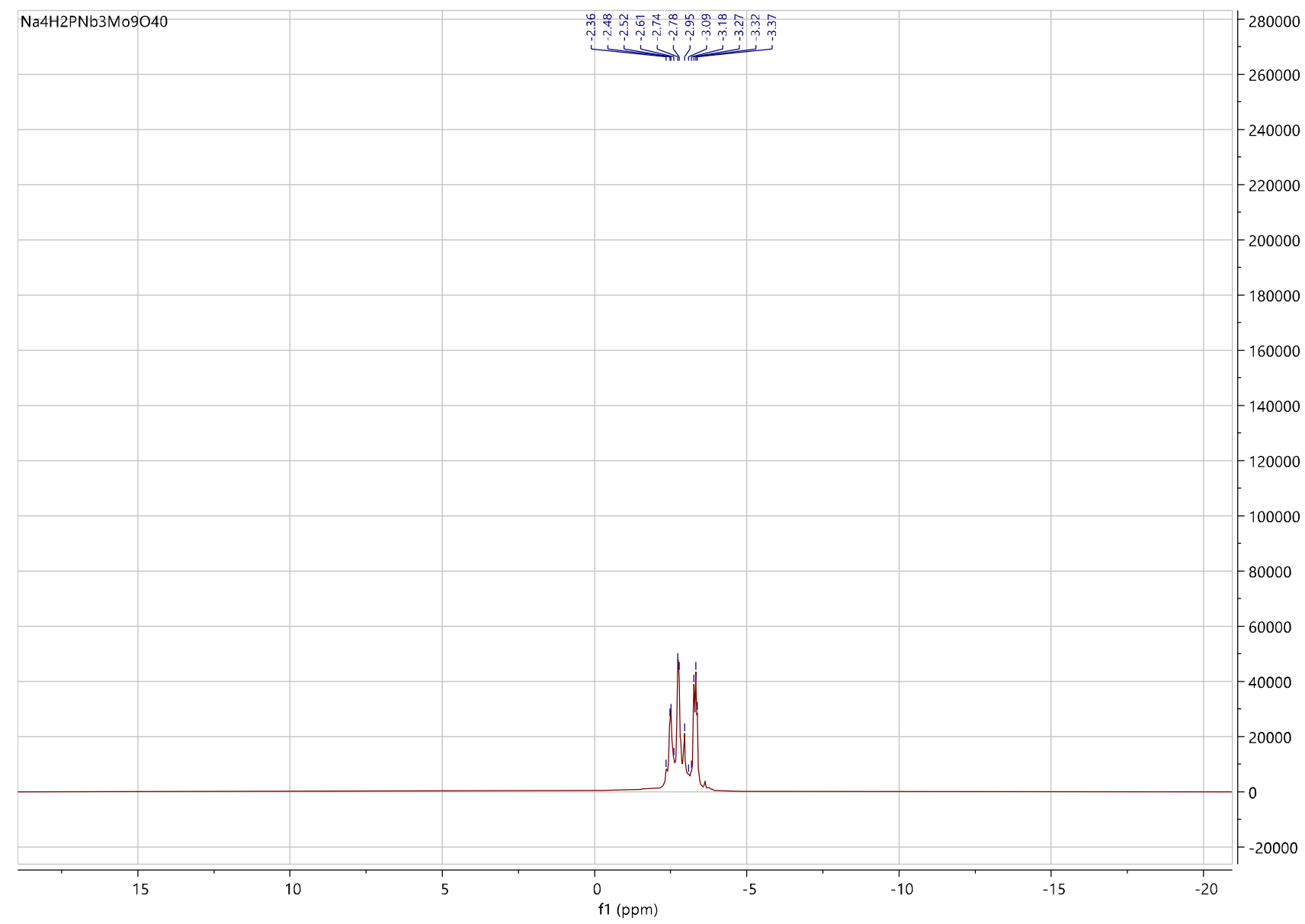

Figure S5: ^{31}P- NMR of the $Na_4H_2PNb_3Mo_9O_{40}$ catalyst.

WD-NaVW ($Na_8HP_2V_3W_{15}O_{62}$)

^{31}P-NMR (242.9 MHz, H_2O/D_2O, 25 °C): δ [ppm] = -11.48, -13.58

^{51}V-NMR (157.8 MHz, H_2O/D_2O, 25 °C): δ [ppm] = -529.06, -530.80, -534.46, -544.95, -546.78, -552.15, -553.60, -554.09, -554.68, -555.79, -558.58

IR (ATR): ṽ [cm^{-1}] = 1620 (O-H, lattice H_2O), 1083, 1022 (w, P-O), 951 ((M=O)t),, 750 ((M-O-M)edge)

ICP-OES: Calculated for $Na_9P_2V_3W_{15}O_{62}$ x 18 H_2O: 1.49 % P, 3.66 % V, 66.11 % W. Found for $Na_9P_2V_3W_{15}O_{62}$ x 18 H_2O: 1.18 % P, 3.73 % V, 60.00 % W. Normalized elemental ratio (V + W = 12): Na/P/V/W ratio: 7.51/1.71/3.30/14.70

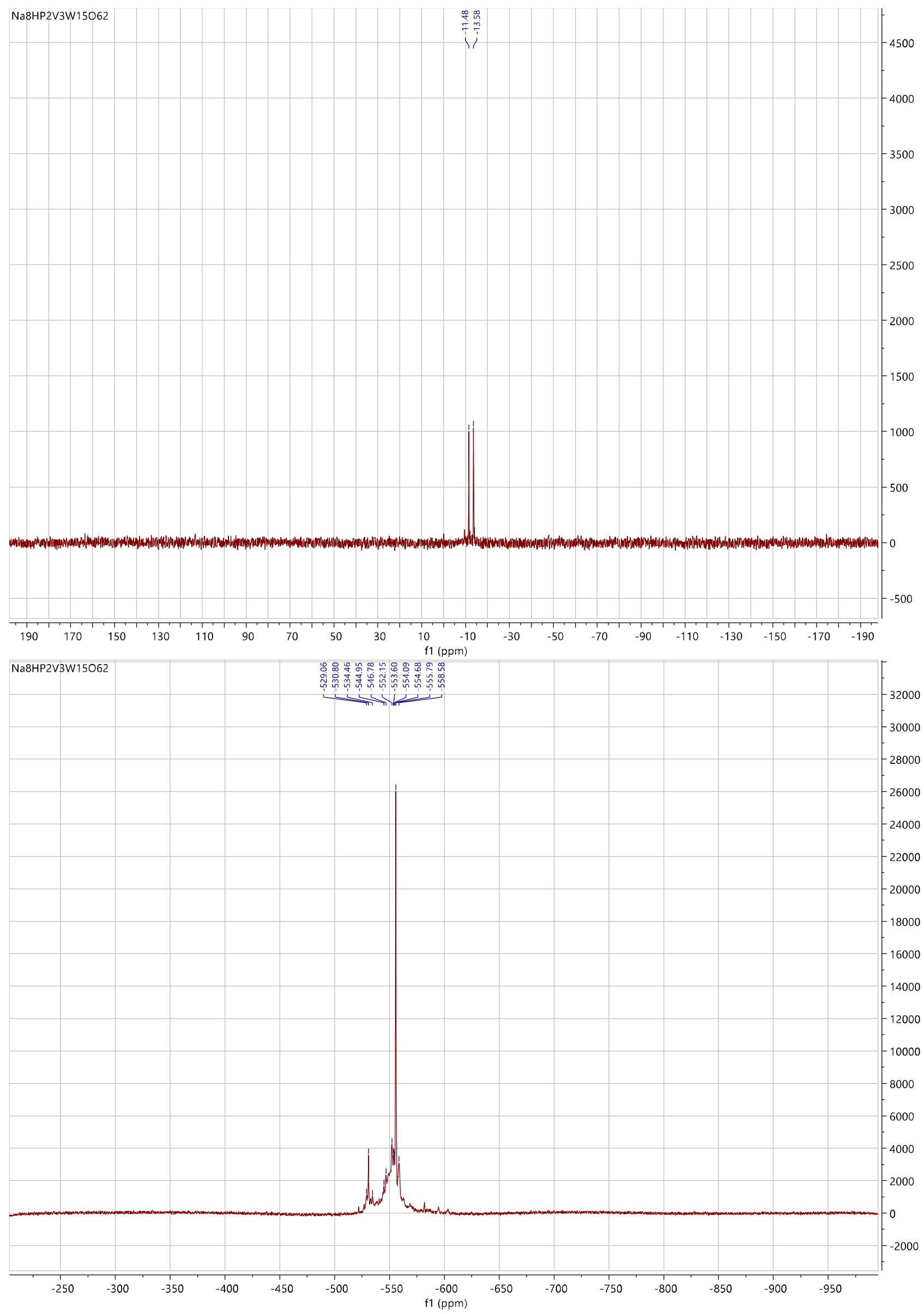

Figure S6: ^{31}P- (top figure) & ^{51}V- NMR (bottom figure) of the $Na_8HP_2V_3W_{15}O_{62}$ catalyst.

WD-HVW ($H_9PV_3W_{15}O_{62}$)

^{31}P-NMR (161.98 MHz, H_2O/D_2O, 26 °C): δ [ppm] = -9.53, -13.04, -14.32

^{51}V-NMR (105.18 MHz, H_2O/D_2O, 26 °C): δ [ppm] = -526.76, -546.78, -550.20, -553.29, -553.98, -555.62, -557.48, -558.68, -560.10, -563.74

IR (ATR): ṽ [cm^{-1}] = 1618 (O-H, lattice H_2O), 1084, 1062 (w, P-O), 958 ((M=O)t),, 882 ((M-O-M)vertex)

ICP-OES: Calculated for $H_9P_2V_3W_{15}O_{62}$ x 10 H_2O: 1.56 % P, 3.85 % V, 69.40 % W. Found for $H_9P_2V_3W_{15}O_{62}$ x 10 H_2O: 1.38 % P, 3.79 % V, 59.53 % W. Normalized elemental ratio (V + W = 12): Na/P/V/W ratio: 0.31/2.01/3.36/14.64

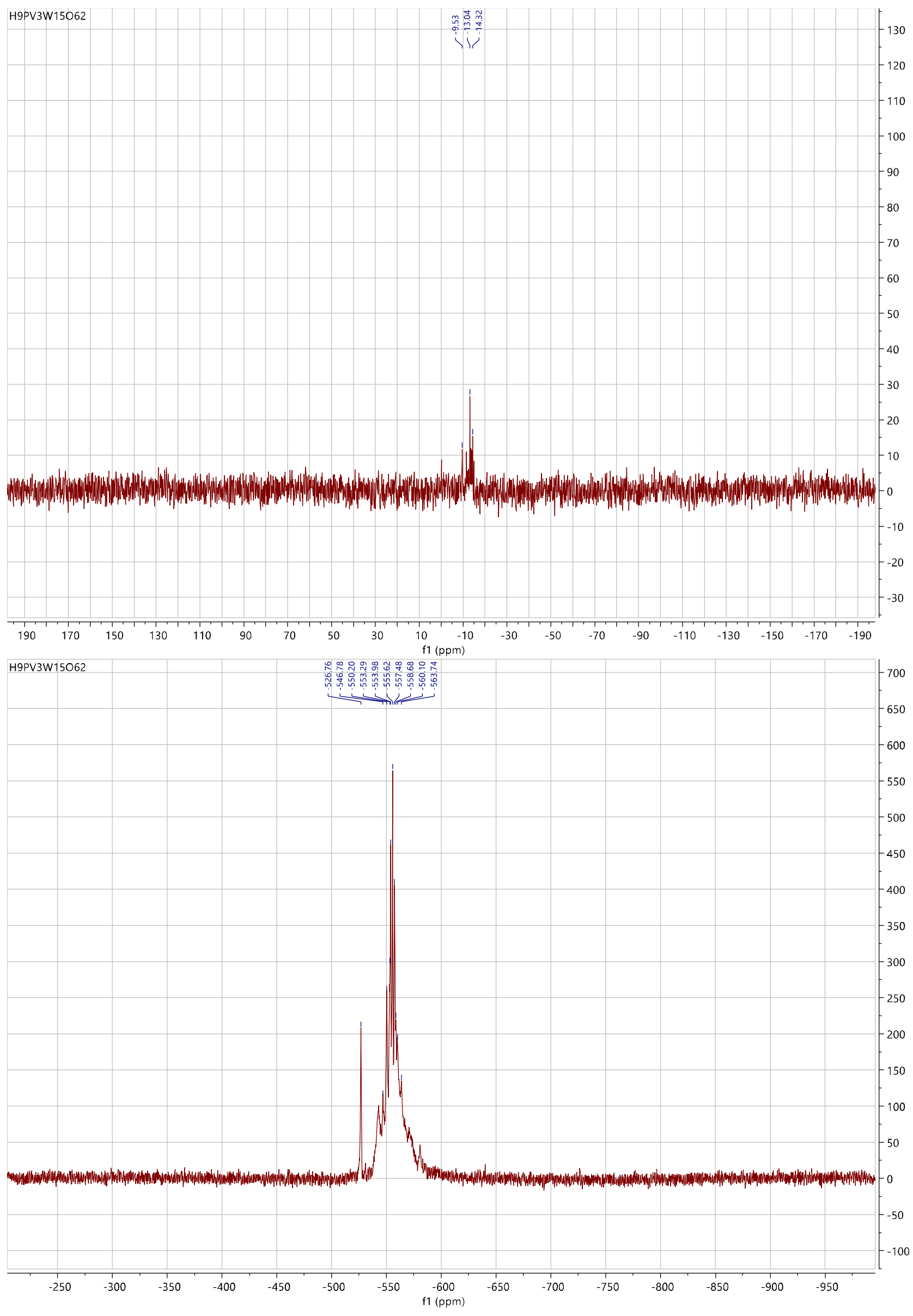

Figure S7: ^{31}P- (top figure) & ^{51}V- NMR (bottom figure) of the $H_9PV_3W_{15}O_{62}$ catalyst.

WD-NaVMo ($Na_7H_2P_2V_3Mo_{15}O_{62}$)

^{31}P-NMR (242.9 MHz, H_2O/D_2O, 25 °C): δ [ppm] = -4.30

^{51}V-NMR (157.8 MHz, H_2O/D_2O, 25 °C): δ [ppm] = -514.82, -529.94, -531.76, -532.40, -532.86

IR (ATR): ṽ [cm^{-1}] = 1624 (O-H, lattice H_2O), 1053 (w, P-O), 944 ((M=O)t),, 860 ((M-O-M)vertex), 747 ((M-O-M)edge).

ICP-OES: Calculated for $Na_9P_2V_3Mo_{15}O_{62}$ x 23 H_2O: 2.17 % P, 5.36 % V, 50.45 % Mo. Found for $Na_9P_2V_3Mo_{15}O_{62}$ x 23 H_2O: 1.54 % P, 5.05 % V, 46.80 % Mo. Normalized elemental ratio (V + W = 12): Na/P/V/Mo ratio: 6.34/1.52/3.04/14.96

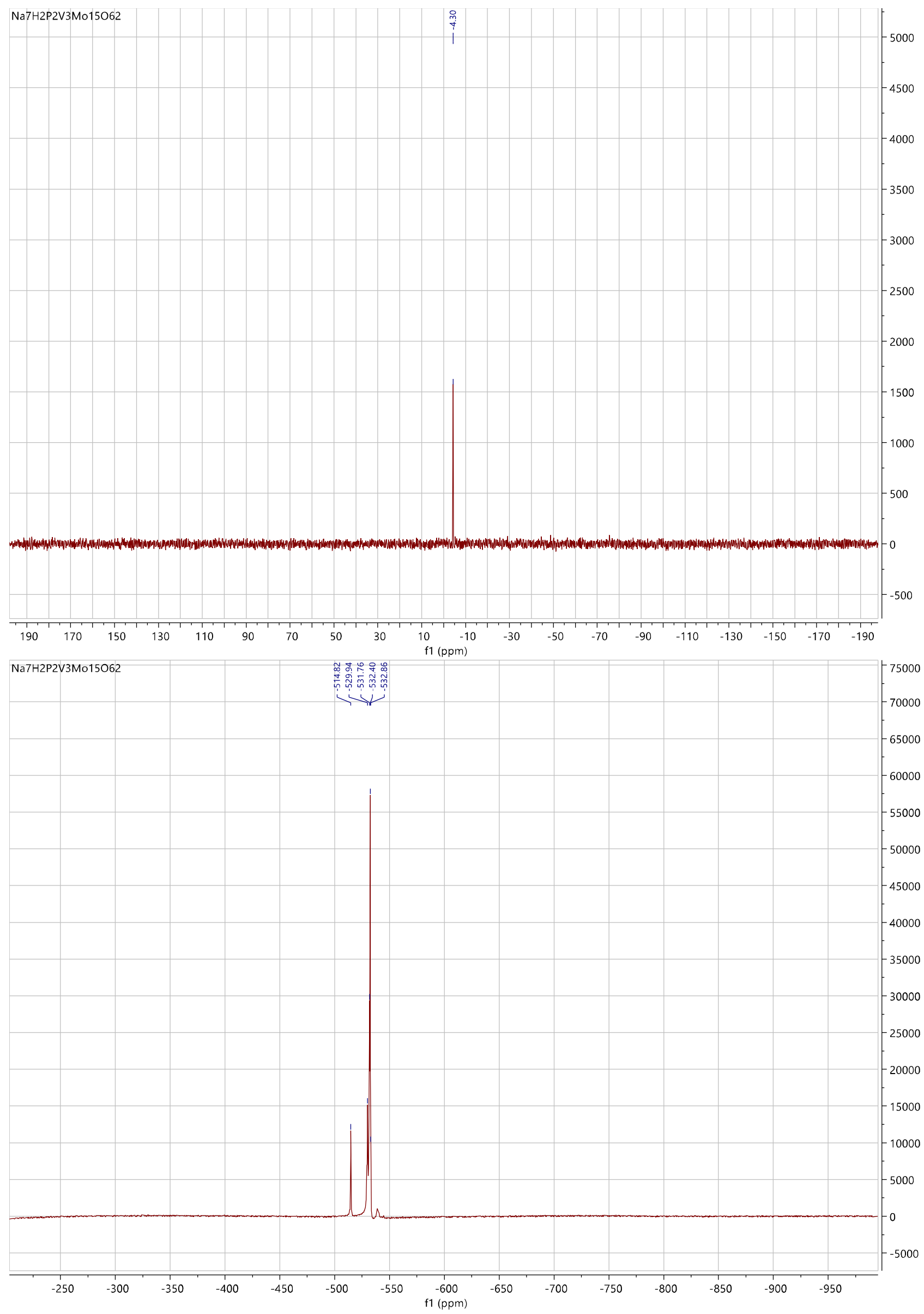

Figure S8: ^{31}P- (top figure) & ^{51}V- NMR (bottom figure) of the $Na_7H_2P_2V_3Mo_{15}O_{62}$ catalyst.

WD-HVMo ($H_9P_2V_3Mo_{15}O_{62}$)

^{31}P-NMR (161.98 MHz, H_2O/D_2O, 26 °C): δ [ppm] = -4.21

^{51}V-NMR (105.18 MHz, H_2O/D_2O, 26 °C): δ [ppm] = -522.40, -531.39, -532.25, -533.25, -533.56, -535.44, -541.39, -542.71

IR (ATR): ṽ [cm^{-1}] = 1600 (O-H, lattice H_2O), 1050(w, P-O), 952 ((M=O)t),, 872 ((M-O-M)vertex)

ICP-OES: Calculated for $H_9P_2V_3Mo_{15}O_{62}$ x 20 H_2O: 2.33 % P, 5.76 % V, 54.21 % Mo. Found for $H_9P_2V_3Mo_{15}O_{62}$ x 20 H_2O: 2.11 % P, 5.70 % V, 52.80 % Mo. Normalized elemental ratio (V + W = 12): Na/P/V/Mo ratio: 0/1.85/3.03/14.97

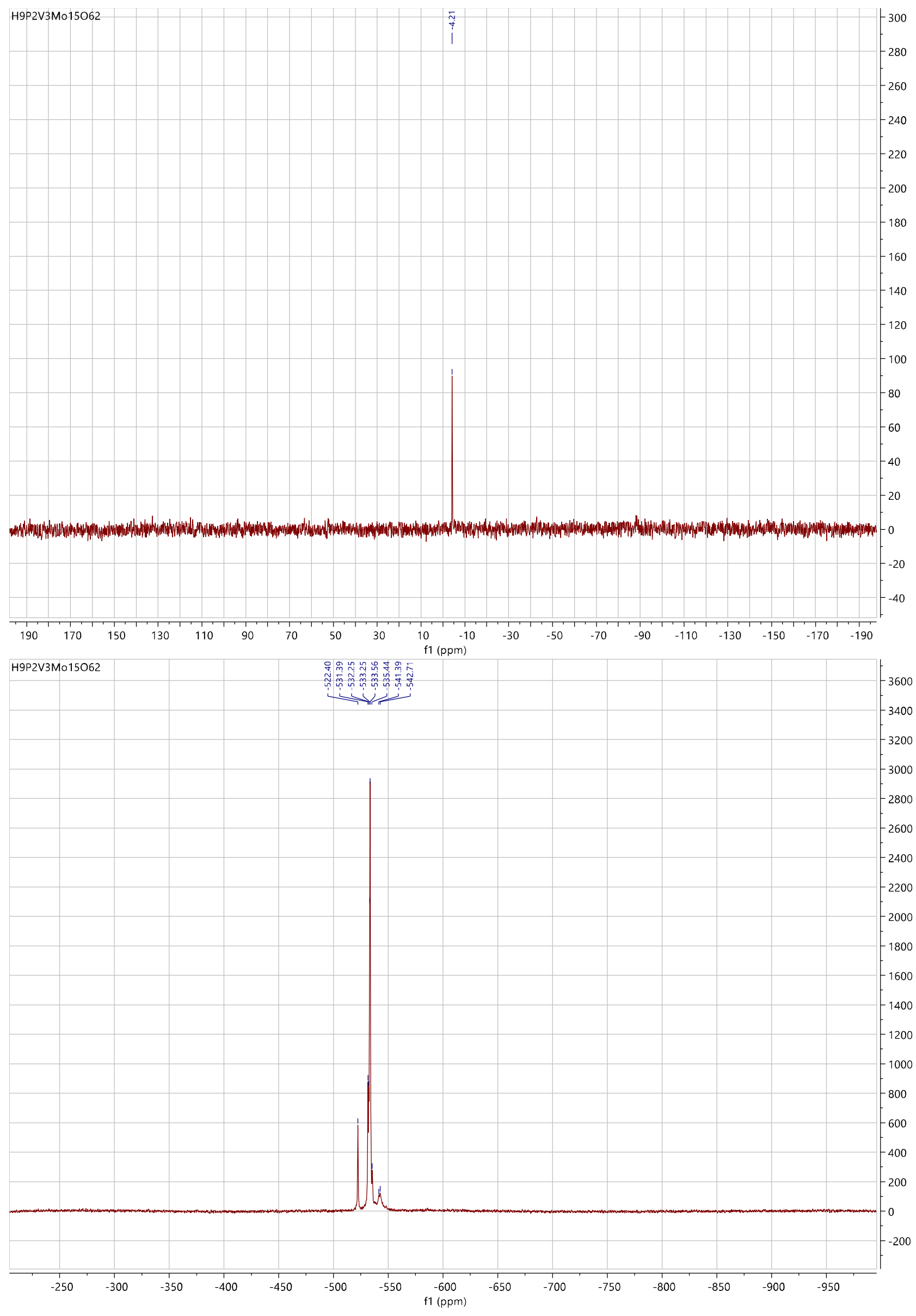

Figure S9: ^{31}P- (top figure) & ^{51}V- NMR (bottom figure) of the $H_9P_2V_3Mo_{15}O_{62}$ catalyst.

L-NaVW ($Na_5V_3W_3O_{19}$)
^{51}V-NMR (157.8 MHz, H_2O/D_2O, 25 °C): δ [ppm] = -509.47, -559.37

IR (ATR): ṽ [cm^{-1}] = 1630 (O-H, lattice H_2O), 931 ((M=O)t),, 871 ((M-O-M)vertex), 716 ((M-O-M)edge).

ICP-OES: Calculated for $Na_5V_3W_3O_{19}$ x 5 H_2O: 13.61 % V, 49.16 % W. Found for $Na_5V_3W_3O_{19}$ x 5 H_2O: 12.27 % V, 45.80 % W. Normalized elemental ratio (V + W = 12): Na/V/W ratio: 4.61/2.95/3.05

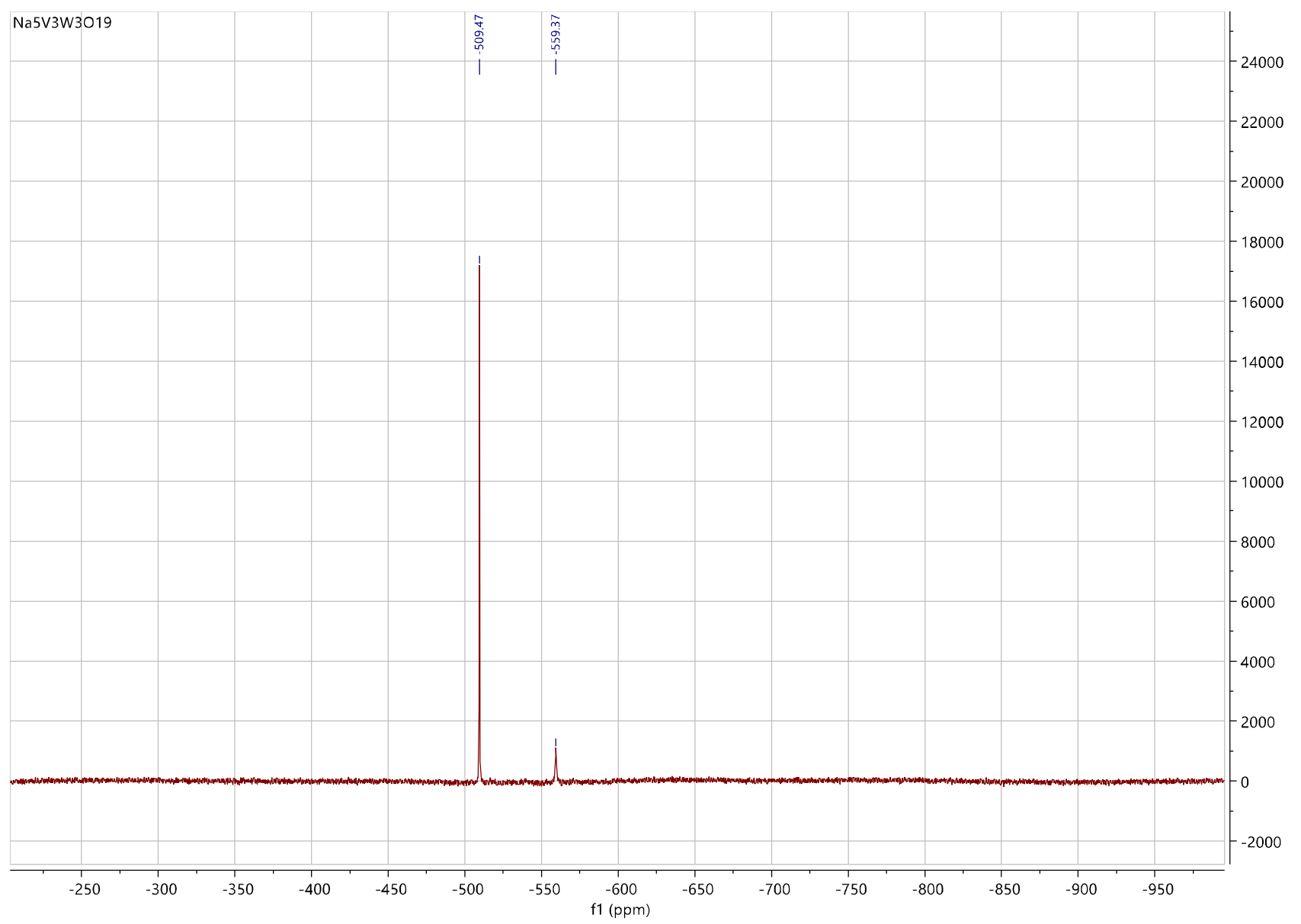

Figure S10: ^{51}V- NMR of the $Na_5V_3W_3O_{19}$ catalyst.

AE-NaVW ($Na_5H_4TeV_3W_3O_{24}$)
^{51}V-NMR (157.8 MHz, H_2O/D_2O, 25 °C): δ [ppm] = -507.36, -508.93

IR (ATR): ṽ [cm^{-1}] = 1623 (O-H, lattice H2O), 947 ((M=O)t),, 886 ((M-O-M)vertex), 677 ((M-O-M)edge).

ICP-OES: Calculated for $Na_9TeV_3W_3O_{24}$ x 5 H_2O: 10.74 % V, 38.76 % W. Found for $Na_9TeV_3W_3O_{24}$ x 5 H_2O: 10.50 % V, 39.90 % W. Normalized elemental ratio (V + W = 12): Na/V/W ratio: 4.72/2.92/3.08

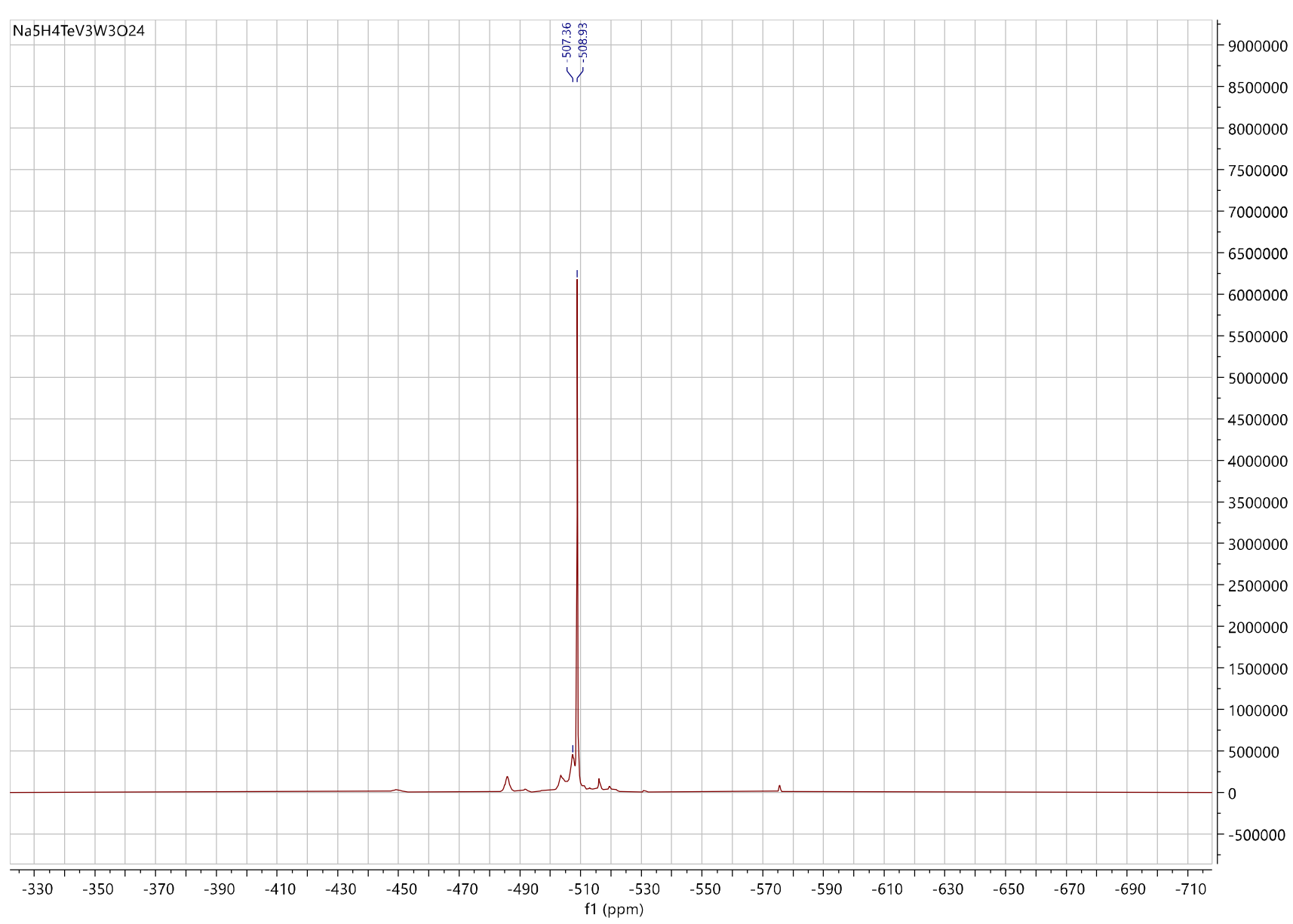

Figure S11: ^{51}V- NMR of the $Na_5H_4TeV_3W_3O_{24}$ catalyst.

Reduced K-HVW ($H_6PV_3W_9O_{40}$)

^{31}P-NMR (161.98 MHz, H_2O/D_2O, 26 °C): δ [ppm] = no signal detected

^{51}V-NMR (105.18 MHz, H_2O/D_2O, 26 °C): δ [ppm] = small signal at -553.74 and -557.11.

IR (ATR): ṽ [cm^{-1}] = 1613 (O-H, lattice H_2O), 1056 (w, P-O), 950 ((M=O)t), 866 ((M-O-M)vertex)

ICP-OES: Calculated for $H_6PV_3W_9O_{40}$ x 14 H_2O: 1.25 % P, 6.15 % V, 66.60% W. Found for $H_6PV_3W_9O_{40}$ x 14 H_2O: 1.76 % P, 5.93 % V, 66.9 % W, 0.08 % Na. Normalized elemental ratio (V + W = 12): Na/P/V/W ratio: 0.38/1.42/2.91/9.09

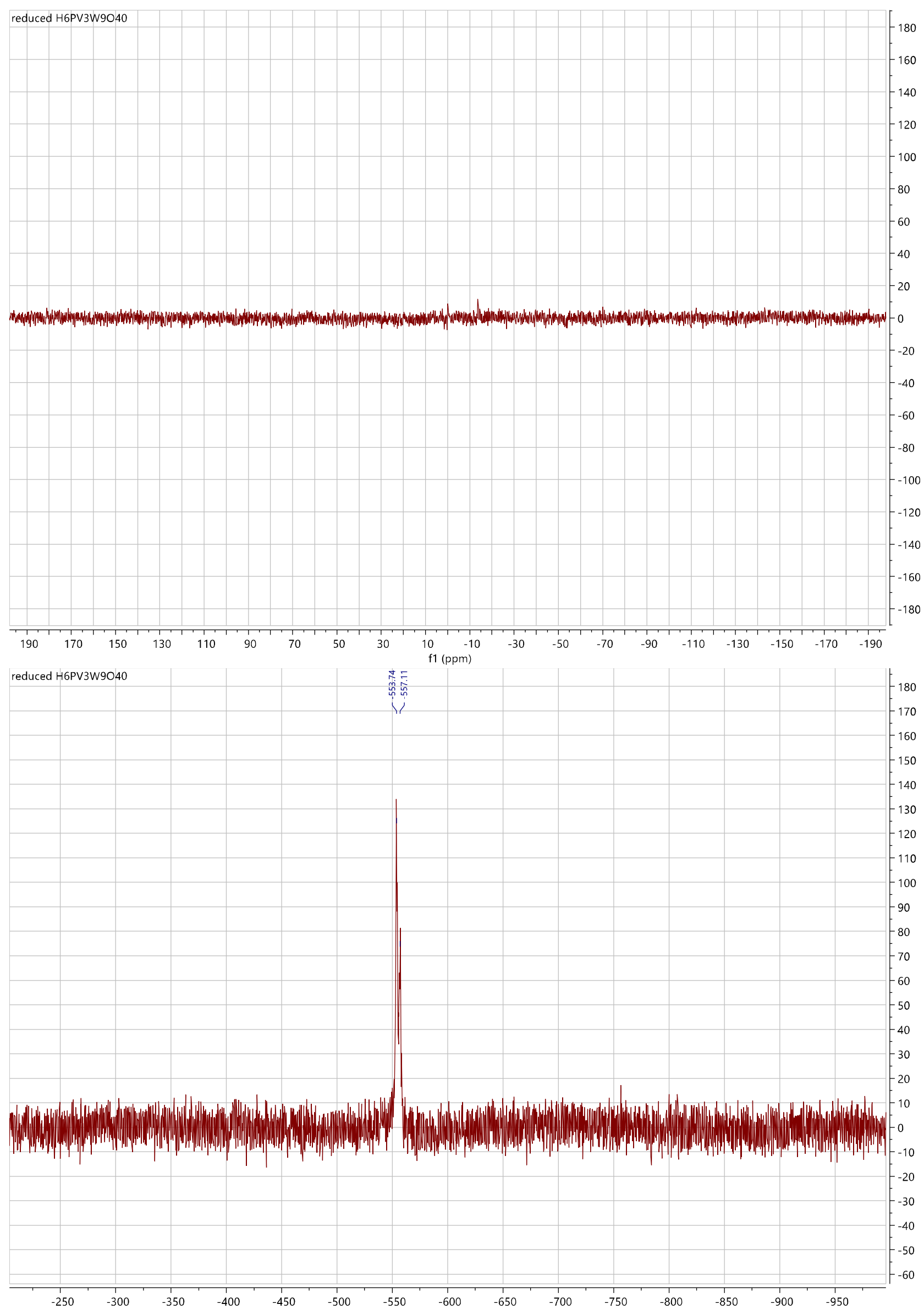

Figure S12: ^{31}P- (top figure) & ^{51}V- NMR (bottom figure) of the reduced $H_6PV_3W_9O_{40}$ catalyst.

IR Spectra of all Catalysts

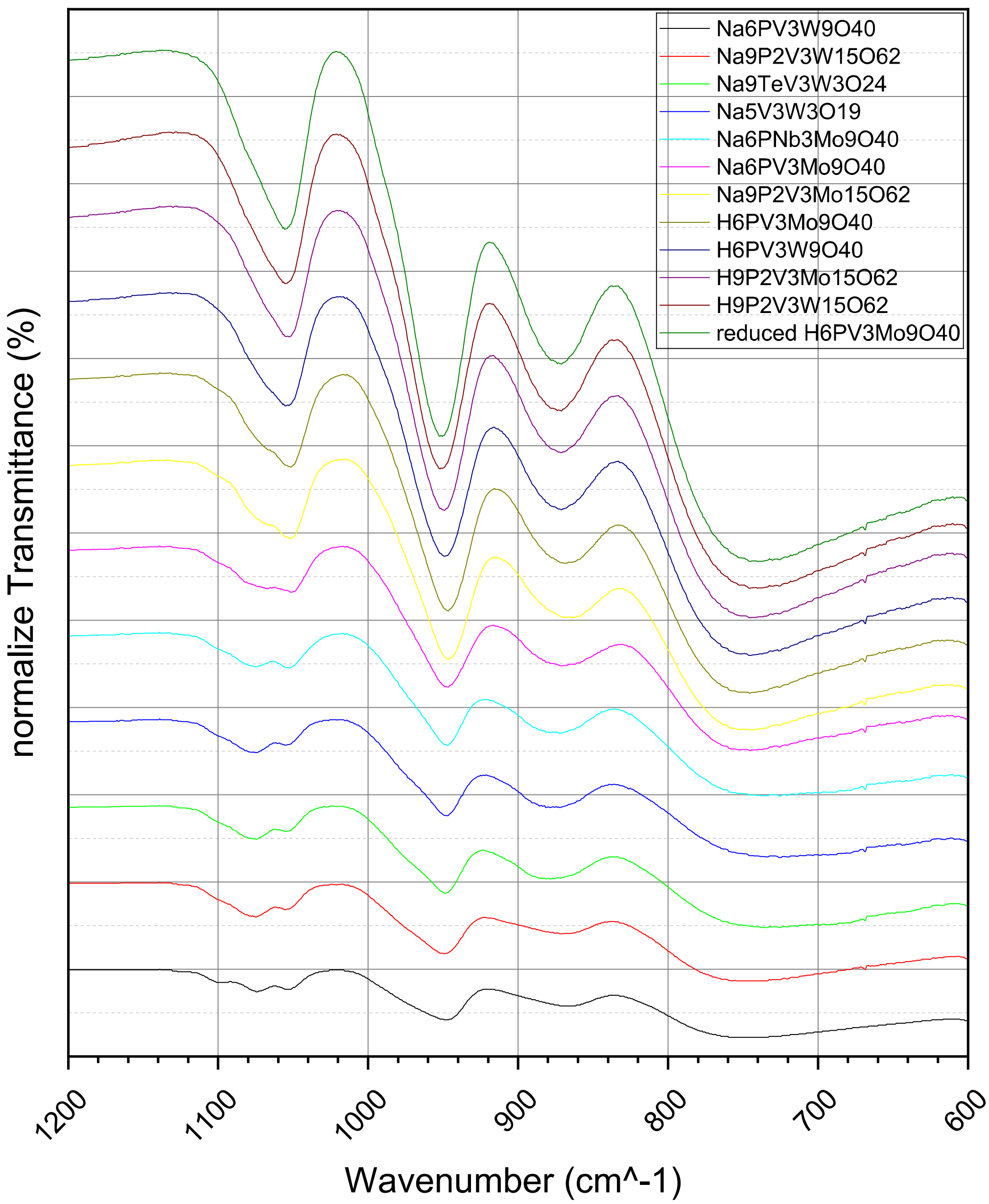

Figure S13: IR spectra of all catalysts for the catalyst screening. Measured between 400 to 4000 cm^{-1}, with 10-15 scans, Apodization Happel-Genzel and a resolution of 2 cm^{-1}.

Flow chart 10-fold plant

The apparatus consists of ten 20 ml autoclaves, which are operated in batch mode. The plant is equipped with a heating plate with ten positions controlled by a Horst HT MC 11 temperature controller, an *IKA RT 10 power* stirring plate and a shared gas line. This configuration facilitates the simultaneous filling of all reactors with the reaction gas and the subsequent application of heat. This enables the uniform attainment of the aforementioned conditions across all ten reactors.

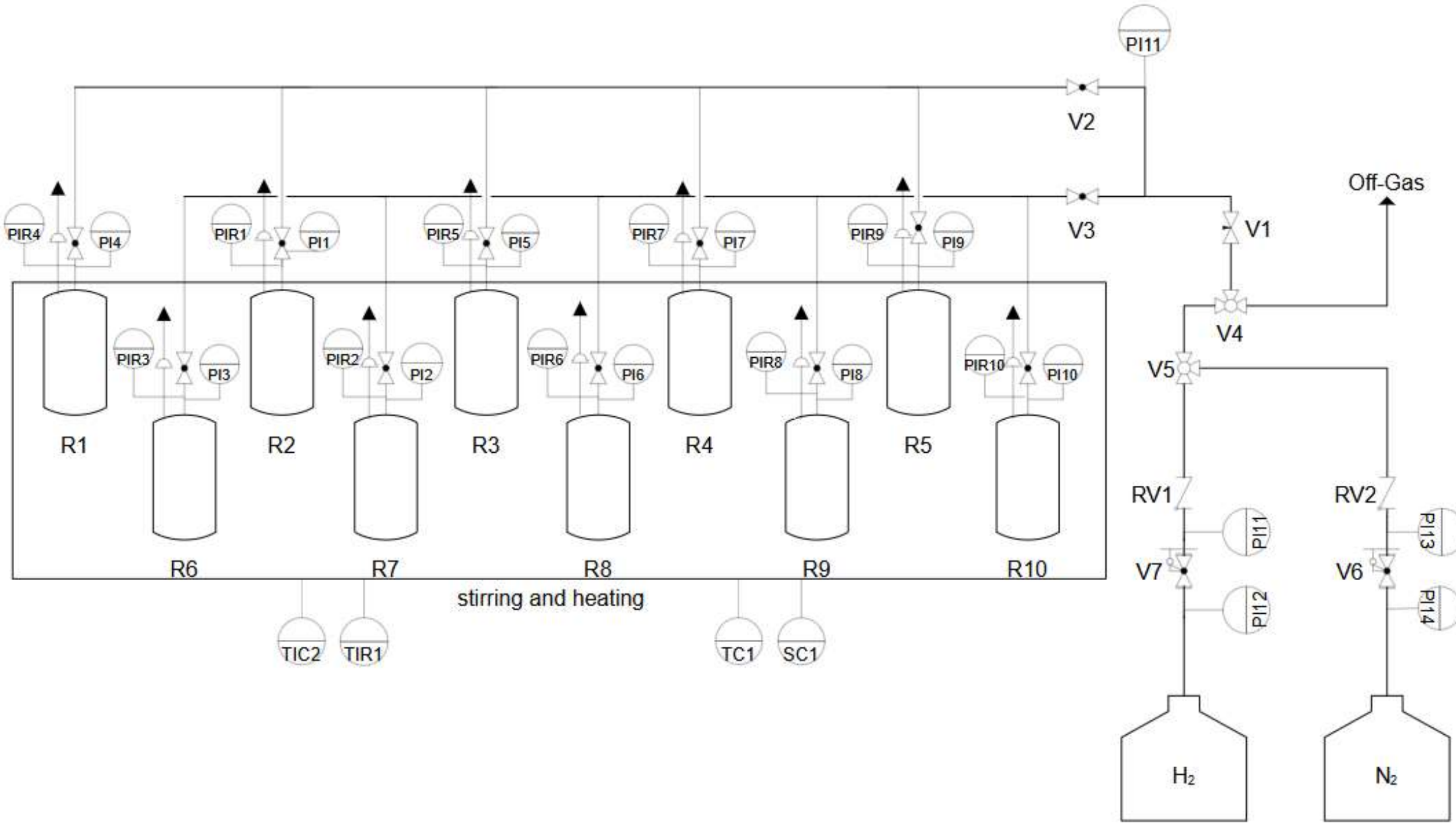

Figure S14: Flow chart of the 10-fold plant.

Sample preparation for downstream processing

After cooling down the reactors by utilizing compressed air, reaction gas samples were filled into gas sample bags for analysis. Subsequently, the reactors were opened and the reaction solutions were filled into 10 ml centrifugation tubes. These were centrifugated for 4 minutes under 10 000 rpm (10.621 g). After that the solution was decantated. The solid was proceed with as explained in the main script.

Used Chemicals

For Catalyst Screening-Experiments, 1 mmol of D-(+)-Glucose (Reag. Ph Eur; anhydrous for biochemistry; CAS 50-99-7; Article-No 1.08337.100) by Merck KGaA was used as the substrate. As commercial catalysts phosphotungstic acid hydrate (reagent grade, CAS: 12501-23-4; PCode 100349030) by Sigma Aldrich and Vanadium (IV)sulfate oxide hydrate ($VOSO_4$; 99,9% metal basis; LOT: S24C023) were used.

For the testing of the intermediates several substrates were used. For C6-substrates 1 mmol, but for C3-substrates 2 mmol and for C2-substrates 3 mmol were used. One of the substrates was D-(-)-Fructose (≥99 %. HPLC; CAS 57-48-7; PCode: 102749622; Source: 0000279985; Product Number: F01271KG) by Sigma Aldrich. Other substrates were Dihydroxyacetone for synthesis (CAS 96-26-4; Product Number: 8.20482.0100) by Sigma Aldrich, DL-Glyceraldehyde (≥90 %. GC; CAS 56-82-6; PCode: 102639337; Source: BCCH8512; Product Number: G5001-25G) by Merck KGaA, pyruvaldehyde (Methylglyoxal solution ~ 40% in H_2O; CAS 78-98-8; PCode: 102540013; Source: BCCH4313) by Sigma - Aldrich and DL-Lactic acid (~ 90 %; CAS 50-21-5; PCode: 102548526; Source: BCCH8222; Product Number: 69785-250ML) by Sigma Aldrich.

The reduction was done with Hydrazin monohydrate (98%; CAS: 7803-57-8, Article Number A14005; LOT: 10236543) by Alfa Aesar.

Details of analytical procedures

Solid sample analysis

The solid material was isolated by centrifugation of the reaction solutions at 10 000 rpm for 4 min using a SIGMA 2-6E type 2-16P centrifuge. The solutions were then decanted. The solid was subjected to a cleaning process in an ultrasonic bath with 10 ml demineralized water for a duration of 10 minutes. After washing the suspension, it was centrifugated under the same conditions again and decanted once more. The liquid was then discarded and the solid was dried in an oven at 90 °C for 48 hours for further analysis. Subsequently, carbon, hydrogen, nitrogen and sulfur (CHNS) analysis was performed. For this purpose, a Unicube device from Elementar was utilized for the analysis of the carbon analysis of the solids. However, due to the low reaction volume, only a limited amount of solid was produced, which occasionally precluded the execution of repeated measurements.

Table S1: average of humin solid samples of catalyst-screening measured with CHNS.

sample	N (%)	C (%)	H (%)	S (%)
**K-NaVW**	0.08	46.86	3.87	0.01
**WD-NaVW**	0.00	64.09	4.88	0.02
**L-NaVW**	0.09	29.88	2.71	0.01
**AE-NaVW**	0.03	30.59	2.71	0.01
**K-NaNbMo**	0.05	50.38	4.13	0.03
**K-NaVMo**	0.10	59.13	4.61	0.00
**K-HVW**	0.05	59.38	4.38	0.03
**K-HVMo**	0.04	55.53	4.13	0.01

Gas samples

The gas phase samples were analysed utilizing the *VARIAN 450-GC* equipped with a *SHIN-CARBON-ST-COLUMN*. The detectors of the apparatus are a thermal conductivity detector (TCD) in conjunction with a flame ionization detector (FID).

HPLC Samples

The liquid samples obtained with the reaction were analysed using a *SHIMADZU NEXERA 40* series HPLC. Separation was performed using a polymer-phase organic acid column (300 mm × 8 mm; CHROMATOGRAPHIE-SERVICE GMBH). The mobile phase was considered of a 4 mmol L^{-1} aqueous sulfuric acid solution. Detection was performed using a refractive index detector (RID) and a photodiode array (PDA).

NMR Samples

This function is performed by the service department of the University of Hamburg. The *Bruker Advance III HD 400 MHz (AVIII400)* device is utilized for this purpose.

Deuterium oxide 99.9% (CA: 7789-20-0; EC: 232-148-9; Product Number: 00606-100ml; LOT B: 28441) by Deutero GmbH was used for NMR samples.

Raman spectroscopy

The Raman measurements were performed using a RAMAN RXN SYSTEMS instrument from KAISER optical systems INC. The probe was connected to a rod. The Laser (SN0075969) had a wavelength of 785 nm. The used software for the measurement was iC Raman-Software™ by Mettler Toledo and for the post-analysis SpectraGryph 1.2 - spectroscopy software from spectroscopy ninja.

Catalyst Screening

All diagrams in the main manuscript are shown in the colour code: yellow for the substrate, green for the pathway to lactic acid, red for the pathway via 5-HMF, purple for the pathway to glycolic acid, and blue for the subsequent pathways of lactic acid. Values are the average of three experiments. The error bars represent the standard deviation. All reactions were performed with the same conditions of 200°C for one hour, a glucose concentration of 0.1 mol L^{-1} and a catalyst concentration of 0.01 mol L^{-1} (based on the vanadium content).

First run

The weight for the glucose-water stock solution was 6.318 g glucose in 350 ml deionized water. This solution was used for the first and second run.

Table S2: weight of catalyst, reactors and filled in solutions and pH measurements for the first run of catalyst screening. Reaction conditions: n(Substrate) = 1 mmol glucose; n(Catalyst) = 0.1 mmol vanadium; V = 10 mL, pN_2 = 20 bar; T = 200 °C; t = 1 h; $V_{stirrer}$ = level 6; Solvent: H_2O.

Catalyst	$m_{catalyst}$ (g)	pH_{before}	$m_{Reactor}$ (g)	$m_{solution}$ (g)	pH_{after}	$m_{solution.after}$ (g)
K-NaVW	0.0860	6.946	341.335	10.252	2.992	10.252
WD-NaVW	0.1374	3.130	340.753	10.288	2.669	10.211
L-NaVW	0.0427	6.566	340.355	10.212	2.892	10.133
AE-NaVW	0.0481	6.251	340.552	10.185	2.771	10.124
K-NaNbMo	0.0670	3.862	340.751	10.196	2.599	10.114
K-NaVMo	0.0762	4.971	336.839	10.124	2.874	10.124
K-HVW	0.0787	2.055	373.475	10.226	2.190	10.169
K-HVMo	0.0547	2.096	336.232	10.223	2.200	10.115
Blank	-	6.247	334.006	10.205	2.810	10.047

Table S3: calculated yields of all detected products for the first run of the catalyst screening. Reaction conditions: n(Substrate) = 1 mmol glucose; n(Catalyst) = 0.1 mmol vanadium; V = 10 mL, pN_2 = 20 bar; T = 200 °C; t = 1 h; $V_{stirrer}$ = level 6; Solvent: H_2O.

Experiment	K-NaVW	WD-NaVW	L-NaVW	AE-NaVW	K-NaNbMo	K-NaVMo	K-HVW	K-HVMo	Blank
$Y_{Glucose}$ (%)	4.05	2.70	4.05	4.05	4.05	5.41	2.70	5.41	5.41
$Y_{Fructose}$ (%)	0.00	0.00	0.00	0.00	4.05	2.70	0.00	0.00	0.00
$Y_{Mannose}$ (%)	2.70	1.35	2.70	2.70	2.70	2.70	0.00	2.70	2.70
Y_{DHA} (%)	0.00	0.00	0.00	0.00	0.00	0.00	0.00	0.00	0.00
$Y_{GlycerAl}$ (%)	2.03	1.35	3.38	2.70	4.05	0.00	1.35	3.38	3.38
$Y_{Pyruvaldehyde}$ (%)	19.59	19.59	18.92	19.59	18.92	18.92	20.27	18.92	18.92
Y_{LA} (%)	0.00	0.00	0.00	0.00	0.00	0.00	23.65	0.00	0.00
Y_{AcetAl} (%)	14.86	6.76	15.32	15.32	13.51	10.81	0.00	0.00	0.00
$Y_{Acetic\ Acid}$ (%)	9.01	6.76	9.46	9.46	8.56	8.11	4.95	4.50	4.50
$Y_{Glycolaldehyde}$ (%)	0.00	0.00	0.00	0.00	0.45	0.00	0.00	0.00	0.00
Y_{GA} (%)	21.17	27.03	0.00	0.00	17.12	17.57	0.00	20.66	20.66
$Y_{5\text{-}HMF}$ (%)	0.00	8.11	2.70	1.35	1.35	2.70	5.41	2.70	2.70
Y_{FA} (%)	6.31	4.95	9.46	9.91	10.36	8.33	5.63	8.56	8.56
Y_{LevA} (%)	0.00	0.00	0.00	0.00	0.00	0.00	13.51	11.26	11.26
$Y_{Glyoxal}$ (%)	0.00	0.00	0.00	0.00	0.00	0.00	0.00	0.00	0.00
$Y_{Succinic\ Acid}$ (%)	0.00	0.00	26.13	25.23	0.00	0.90	1.80	0.00	0.00
Y_{CO_2} (%)	3.55	3.06	3.41	3.31	2.92	3.92	2.75	2.26	0.78
$Y_{Conversion}$ (%)	95.95	97.30	95.95	95.95	95.95	94.59	97.30	94.59	56.76
$Y_{Carbon\ Balance}$ (%)	83.28	81.66	95.53	93.62	88.06	82.07	82.03	80.35	84.33

Table S4: calculated selectivitys of all detected products for the first run of the catalyst screening. Reaction conditions: n(Substrate) = 1 mmol glucose; n(Catalyst) = 0.1 mmol vanadium; V = 10 mL, pN_2 = 20 bar; T = 200 °C; t = 1 h; $V_{stirrer}$ = level 6; Solvent: H_2O.

Experiment	K-NaVW	WD-NaVW	L-NaVW	AE-NaVW	K-NaNbMo	K-NaVMo	K-HVW	K-HVMo	Blank
$S_{Glucose}$ (%)	4.23	2.78	4.23	4.23	4.23	5.71	2.78	5.71	76.19
$S_{Fructose}$ (%)	0.00	0.00	0.00	0.00	4.23	2.86	0.00	0.00	0.00
$S_{Mannose}$ (%)	2.82	1.39	2.82	2.82	2.82	2.86	0.00	2.86	16.67
S_{DHA} (%)	0.00	0.00	0.00	0.00	0.00	0.00	0.00	0.00	0.00
$S_{GlycerAl}$ (%)	2.11	1.39	3.52	2.82	4.23	0.00	1.39	3.57	0.00
$S_{Pyruvaldehyde}$ (%)	20.42	20.14	19.72	0.00	19.72	20.00	20.83	20.00	0.00
S_{LA} (%)	0.00	0.00	0.00	0.00	0.00	0.00	24.31	0.00	0.00
S_{AcetAl} (%)	15.49	6.94	15.96	15.96	14.08	11.43	0.00	0.00	0.00
$S_{Acetic\ Acid}$ (%)	9.39	6.94	9.86	9.86	8.92	8.57	5.09	4.76	0.00
$S_{Glycolaldehyde}$ (%)	0.00	0.00	0.00	0.00	0.47	0.00	0.00	0.00	0.79
S_{GA} (%)	22.07	27.78	0.00	0.00	17.84	18.57	0.00	21.84	0.00
$S_{5\text{-}HMF}$ (%)	0.00	8.33	2.82	1.41	1.41	2.86	5.56	2.86	42.86
S_{FA} (%)	6.57	5.09	9.86	10.33	10.80	8.81	5.79	9.05	1.19
S_{LevA} (%)	0.00	0.00	0.00	0.00	0.00	0.00	13.89	11.90	0.00
$S_{Glyoxal}$ (%)	0.00	0.00	0.00	0.00	0.00	0.00	0.00	0.00	0.00
$S_{Succinic\ Acid}$ (%)	0.00	0.00	27.23	26.29	0.00	0.95	1.85	0.00	9.52
S_{CO_2} (%)	3.70	3.15	3.55	3.45	3.04	4.14	2.82	2.39	1.37

Second run

Table S5: weight of catalyst, reactors and filled in solutions and pH measurements for the second run of catalyst screening. Reaction conditions: n(Substrate) = 1 mmol glucose; n(Catalyst) = 0.1 mmol vanadium; V = 10 mL, pN_2 = 20 bar; T = 200 °C; t = 1 h; $V_{stirrer}$ = level 6; Solvent: H_2O.

Catalyst	$m_{catalyst}$ (g)	pH_{before}	$m_{Reactor}$ (g)	$m_{solution}$ (g)	pH_{after}	$m_{solution.after}$ (g)
K-NaVMo	0.0773	4.805	341.339	10.247	2.831	10.105
K-NaNbMo	0.0661	3.854	340.761	10.177	2.558	10.046
K-HVW	0.0800	2.048	340.373	10.241	2.142	10.090
K-HVMo	0.0538	2.078	340.562	10.228	2.205	10.136
K-NaVW	0.0860	6.785	340.760	10.236	2.981	10.188
WD-NaVW	0.1371	3.052	336.856	10.229	2.660	10.189
L-NaVW	0.0434	6.610	373.487	9.585	2.921	10.105
AE-NaVW	0.0483	6.129	336.242	10.220	2.899	10.077
Blank	-	6.247	334.018	10.155	2.922	9.994

Table S6: calculated yields of all detected products for the second run of the catalyst screening. Reaction conditions: n(Substrate) = 1 mmol glucose; n(Catalyst) = 0.1 mmol vanadium; V = 10 mL, pN_2 = 20 bar; T = 200 °C; t = 1 h; $V_{stirrer}$ = level 6; Solvent: H_2O.

Experiment	K-NaVMo	K-NaNbMo	K-HVW	K-HVMo	K-NaVW	WD-NaVW	L-NaVW	AE-NaVW	Blank
$Y_{Glucose}$ (%)	5.33	2.67	2.67	4.00	4.00	2.67	4.00	4.00	53.33
$Y_{Fructose}$ (%)	4.00	4.00	0.00	0.00	0.00	0.00	0.00	0.00	5.33
$Y_{Mannose}$ (%)	2.67	2.67	0.00	2.67	2.67	1.33	2.67	2.67	0.00
Y_{DHA} (%)	0.00	0.00	0.00	0.00	0.00	0.00	0.00	0.00	0.00
$Y_{GlycerAl}$ (%)	0.00	3.33	2.00	3.33	2.00	0.67	3.33	2.67	0.00
$Y_{Pyruvaldehyde}$ (%)	18.67	18.67	19.33	18.67	19.33	18.67	19.33	19.33	0.00
Y_{LA} (%)	0.00	0.00	24.67	0.00	0.00	0.00	0.00	0.00	0.00
Y_{AcetAl} (%)	13.78	12.89	0.00	0.00	11.11	6.67	14.67	15.11	0.00
$Y_{Acetic\ Acid}$ (%)	8.89	8.89	4.89	4.89	7.56	6.67	9.78	9.33	0.00
$Y_{Glycolaldehyde}$ (%)	0.44	0.44	0.89	0.44	0.00	0.00	0.00	0.00	0.00
Y_{GA} (%)	18.22	17.33	0.00	20.51	20.89	23.47	0.00	0.00	0.00
$Y_{5\text{-}HMF}$ (%)	2.67	2.67	2.67	4.00	6.67	9.33	2.67	2.67	25.33
Y_{FA} (%)	9.78	10.89	6.44	9.33	4.44	4.44	9.11	10.00	0.44
Y_{LevA} (%)	0.00	0.00	14.44	11.11	0.00	0.00	0.00	0.00	0.00
$Y_{Glyoxal}$ (%)	0.00	0.00	0.00	0.00	0.00	0.00	0.00	0.00	0.00
$Y_{Succinic\ Acid}$ (%)	0.00	0.00	0.00	0.00	0.00	0.00	25.78	24.00	4.44
Y_{CO_2} (%)	4.33	3.67	2.79	2.43	3.84	3.08	3.59	3.39	0.51
$Y_{Conversion}$ (%)	94.67	97.33	97.33	96.00	96.00	97.33	96.00	96.00	46.67
$Y_{Carbon\ Balance}$ (%)	88.78	88.11	80.79	81.38	82.50	76.99	94.92	93.17	89.40

Table S7: calculated selectivity's of all detected products for the second run of the catalyst screening. Reaction conditions: n(Substrate) = 1 mmol glucose; n(Catalyst) = 0.1 mmol vanadium; V = 10 mL, pN_2 = 20 bar; T = 200 °C; t = 1 h; $V_{stirrer}$ = level 6; Solvent: H_2O.

Experiment	K-NaVMo	K-NaNbMo	K-HVW	K-HVMo	K-NaVW	WD-NaVW	L-NaVW	AE-NaVW	Blank
$S_{Glucose}$ (%)	5.63	2.74	2.74	4.17	4.17	2.74	4.17	4.17	114.29
$S_{Fructose}$ (%)	4.23	4.11	0.00	0.00	0.00	0.00	0.00	0.00	11.43
$S_{Mannose}$ (%)	2.82	2.74	0.00	2.78	2.78	1.37	2.78	2.78	0.00
S_{DHA} (%)	0.00	0.00	0.00	0.00	0.00	0.00	0.00	0.00	0.00
$S_{GlycerAl}$ (%)	0.00	3.42	2.05	3.47	2.08	0.68	3.47	2.78	0.00
$S_{Pyruvaldehyde}$ (%)	19.72	19.18	19.86	0.00	20.14	19.18	20.14	20.14	0.00
S_{LA} (%)	0.00	0.00	25.34	0.00	0.00	0.00	0.00	0.00	0.00
S_{AcetAl} (%)	14.55	13.24	0.00	0.00	11.57	6.85	15.28	15.74	0.00
$S_{Acetic\ Acid}$ (%)	9.39	9.13	5.02	5.09	7.87	6.85	10.19	9.72	0.00
$S_{Glycolaldehyde}$ (%)	0.47	0.46	0.91	0.46	0.00	0.00	0.00	0.00	0.00
S_{GA} (%)	19.25	17.81	0.00	21.36	21.76	24.11	0.00	0.00	0.00
$S_{5\text{-}HMF}$ (%)	2.82	2.74	2.74	4.17	6.94	9.59	2.78	2.78	54.29
S_{FA} (%)	10.33	11.19	6.62	9.72	4.63	4.57	9.49	10.42	0.95
S_{LevA} (%)	0.00	0.00	14.84	11.57	0.00	0.00	0.00	0.00	0.00
$S_{Glyoxal}$ (%)	0.00	0.00	0.00	0.00	0.00	0.00	0.00	0.00	0.00
$S_{Succinic\ Acid}$ (%)	0.00	0.00	0.00	0.00	0.00	0.00	26.85	25.00	9.52
S_{CO_2} (%)	4.58	3.77	2.86	2.53	4.00	3.16	3.74	3.53	1.09

Third run

The weight for the glucose-water stock solution was 1.9813 g glucose in 110 ml deionized water.

Table S8: weight of catalyst, reactors and filled in solutions and pH measurements for the third run of catalyst screening. Reaction conditions: n(Substrate) = 1 mmol glucose; n(Catalyst) = 0.1 mmol vanadium; V = 10 mL, pN_2 = 20 bar; T = 200 °C; t = 1 h; $V_{stirrer}$ = level 6; Solvent: H_2O.

Reactor	$m_{catalyst}$ (g)	pH_{before}	$m_{Reactor}$ (g)	$m_{solution}$ (g)	pH_{after}	$m_{solution.after}$ (g)
Blank	-	2.958	341.317	10.177	2.958	10.076
WD-NaVW	0.1360	3.162	340.720	10.240	2.662	10.203
K-NaVW	0.0855	6.731	340.344	10.137	3.003	9.999
AE-NaVW	0.0489	6.189	340.541	10.097	20834	9.966
L-NaVW	0.0426	6.513	340.740	10.128	2.897	10.007
K-NaNbMo	0.0645	3.882	336.835	10.147	2.565	9.952
K-HVMo	0.0536	2.096	373.519	10.133	2.191	9.928
K-NaVMo	0.0756	4.833	336.184	10.173	2.865	10.091
K-HVW	0.0789	2.014	333.999	10.166	2.130	10.040

Table S9: calculated yields of all detected products for the third run of the catalyst screening. Reaction conditions: n(Substrate) = 1 mmol glucose; n(Catalyst) = 0.1 mmol vanadium; V = 10 mL, pN_2 = 20 bar; T = 200 °C; t = 1 h; $V_{stirrer}$ = level 6; Solvent: H_2O.

Experiment	Blank	WD-NaVW	K-NaVW	AE-NaVW	L-NaVW	K-NaNbMo	K-HVMo	K-NaVMo	K-HVW
$Y_{Glucose}$ (%)	48.00	2.67	4.00	4.00	4.00	4.00	5.33	4.00	2.67
$Y_{Fructose}$ (%)	5.33	0.00	0.00	0.00	0.00	6.67	0.00	2.67	0.00
$Y_{Mannose}$ (%)	0.00	0.00	4.00	4.00	2.67	0.00	2.67	1.33	1.33
Y_{DHA} (%)	0.00	0.00	0.00	0.00	0.00	0.00	0.00	0.00	0.00
$Y_{GlycerAl}$ (%)	0.00	2.67	1.33	2.67	2.67	4.67	4.00	0.00	2.00
$Y_{Pyruvaldehyde}$ (%)	0.00	18.67	18.67	18.67	18.67	18.00	19.33	18.00	19.33
Y_{LA} (%)	0.00	0.00	0.00	0.00	0.00	0.00	0.00	0.00	26.67
Y_{AcetAl} (%)	0.00	8.89	5.78	13.33	14.67	13.78	0.00	10.22	0.00
$Y_{Acetic\ Acid}$ (%)	0.00	7.11	10.22	9.78	9.78	10.22	4.44	8.89	4.44
$Y_{Glycolaldehyde}$ (%)	0.89	0.00	0.00	0.00	0.00	0.00	0.44	0.00	0.00
Y_{GA} (%)	0.00	24.26	20.00	0.00	0.00	17.33	21.43	15.11	0.00
$Y_{5\text{-}HMF}$ (%)	30.67	10.67	5.33	2.67	2.67	2.67	5.33	2.67	4.00
Y_{FA} (%)	0.00	4.67	6.89	9.78	9.33	10.44	9.11	7.11	6.00
Y_{LevA} (%)	0.00	2.22	0.00	0.00	0.00	0.00	10.00	0.00	14.44
$Y_{Glyoxal}$ (%)	0.00	0.00	0.00	0.00	0.00	0.00	0.00	0.00	0.00
$Y_{Succinic\ Acid}$ (%)	5.33	0.00	0.00	24.00	24.00	0.00	0.00	0.00	0.00
Y_{CO_2} (%)	0.63	0.00	3.25	3.19	3.09	3.20	2.51	3.59	3.47
$Y_{Conversion}$ (%)	52.00	97.33	96.00	96.00	96.00	96.00	94.67	96.00	97.33
$Y_{Carbon\ Balance}$ (%)	90.86	81.82	79.47	92.08	91.53	90.98	84.61	73.59	84.36

Table S10: calculated selectivity's of all detected products for the third run of the catalyst screening. Reaction conditions: n(Substrate) = 1 mmol glucose; n(Catalyst) = 0.1 mmol vanadium; V = 10 mL, pN_2 = 20 bar; T = 200 °C; t = 1 h; $V_{stirrer}$ = level 6; Solvent: H_2O.

Experiment	Blank	WD-NaVW	K-NaVW	AE-NaVW	L-NaVW	K-NaNbMo	K-HVMo	K-NaVMo	K-HVW
$S_{Glucose}$ (%)	92.31	2.74	4.17	4.17	4.17	4.17	5.63	4.17	2.74
$S_{Fructose}$ (%)	10.26	0.00	0.00	0.00	0.00	6.94	0.00	2.78	0.00
$S_{Mannose}$ (%)	0.00	0.00	4.17	4.17	2.78	0.00	2.82	1.39	1.37
S_{DHA} (%)	0.00	0.00	0.00	0.00	0.00	0.00	0.00	0.00	0.00

$S_{GlycerAl}$ (%)	0.00	2.74	1.39	2.78	2.78	4.86	4.23	0.00	2.05
$S_{Pyruvaldehyde}$ (%)	0.00	19.18	19.44	0.00	19.44	18.75	20.42	18.75	19.86
S_{LA} (%)	0.00	0.00	0.00	0.00	0.00	0.00	0.00	0.00	27.40
S_{AcetAl} (%)	0.00	9.13	6.02	13.89	15.28	14.35	0.00	10.65	0.00
$S_{Acetic\ Acid}$ (%)	0.00	7.31	10.65	10.19	10.19	10.65	4.69	9.26	4.57
$S_{Glycolaldehyde}$ (%)	1.71	0.00	0.00	0.00	0.00	0.00	0.47	0.00	0.00
S_{GA} (%)	0.00	24.93	20.83	0.00	0.00	18.06	22.64	15.74	0.00
$S_{5\text{-}HMF}$ (%)	58.97	10.96	5.56	2.78	2.78	2.78	5.63	2.78	4.11
S_{FA} (%)	0.00	4.79	7.18	10.19	9.72	10.88	9.62	7.41	6.16
S_{LevA} (%)	0.00	2.28	0.00	0.00	0.00	0.00	10.56	0.00	14.84
$S_{Glyoxal}$ (%)	0.00	0.00	0.00	0.00	0.00	0.00	0.00	0.00	0.00
$S_{Succinic\ Acid}$ (%)	10.26	0.00	0.00	25.00	25.00	0.00	0.00	0.00	0.00
S_{CO_2} (%)	1.22	0.00	3.38	3.32	3.22	3.34	2.65	3.74	3.57

Replication of WD-NaVW

The weight for the glucose-water stock solution was 0.451 g glucose in 25 ml deionized water.

Table S11: weight of catalyst, reactors and filled in solutions and pH measurements for the replication of WD-NaVW of catalyst screening. Reaction conditions: n(Substrate) = 1 mmol glucose; n(Catalyst) = 0.1 mmol vanadium; V = 10 mL, pN_2 = 20 bar; T = 200 °C; t = 1 h; $V_{stirrer}$ = level 6; Solvent: H_2O.

Reactor	$m_{catalyst}$ (g)	pH_{before}	$m_{Reactor}$ (g)	$m_{solution}$ (g)	pH_{after}	$m_{solution.after}$ (g)
WD-NaVW	0.1359	3.155	340.573	10.237	2.648	10.127
Blank	-	5.727	336.834	10.180	2.873	10.079

Table S12: calculated yields of all detected products for the replication of WD-NaVW of catalyst screening. Reaction conditions: n(Substrate) = 1 mmol glucose; n(Catalyst) = 0.1 mmol vanadium; V = 10 mL, pN_2 = 20 bar; T = 200 °C; t = 1 h; $V_{stirrer}$ = level 6; Solvent: H_2O.

Experiment	WD-NaVW	Blank
$Y_{Glucose}$ (%)	2.67	36.00
$Y_{Fructose}$ (%)	0.00	0.00
$Y_{Mannose}$ (%)	1.33	12.00
Y_{DHA} (%)	0.00	0.00
$Y_{GlycerAl}$ (%)	2.00	0.00
$Y_{Pyruvaldehyde}$ (%)	19.33	20.00
Y_{LA} (%)	0.00	0.00
Y_{AcetAl} (%)	10.22	0.00
$Y_{Acetic\ Acid}$ (%)	8.00	0.00
$Y_{Glycolaldehyde}$ (%)	0.00	0.00
Y_{GA} (%)	22.87	0.00
$Y_{5\text{-}HMF}$ (%)	10.67	28.00
Y_{FA} (%)	6.00	0.67
Y_{LevA} (%)	2.22	0.00
$Y_{Glyoxal}$ (%)	0.00	0.00
$Y_{Succinic\ Acid}$ (%)	0.00	8.00
Y_{CO_2} (%)	3.01	0.98
$Y_{Conversion}$ (%)	97.33	64.00
$Y_{Carbon\ Balance}$ (%)	88.32	105.64

Table S131: calculated selectivity's of all detected products for the replication of WD-NaVW of catalyst screening. Reaction conditions: n(Substrate) = 1 mmol glucose; n(Catalyst) = 0.1 mmol vanadium; V = 10 mL, pN_2 = 20 bar; T = 200 °C; t = 1 h; $V_{stirrer}$ = level 6; Solvent: H_2O.

Experiment	WD-NaVW	Blank
$S_{Glucose}$ (%)	2.74	56.25
$S_{Fructose}$ (%)	0.00	0.00
$S_{Mannose}$ (%)	1.37	18.75
S_{DHA} (%)	0.00	0.00
$S_{GlycerAl}$ (%)	2.05	0.00
$S_{Pyruvaldehyde}$ (%)	0.00	31.25
S_{LA} (%)	0.00	0.00
S_{AcetAl} (%)	10.50	0.00
$S_{Acetic\ Acid}$ (%)	8.22	0.00
$S_{Glycolaldehyde}$ (%)	0.00	0.00
S_{GA} (%)	23.50	0.00
$S_{5\text{-}HMF}$ (%)	10.96	43.75
S_{FA} (%)	6.16	1.04
S_{LevA} (%)	2.28	0.00
$S_{Glyoxal}$ (%)	0.00	0.00
$S_{Succinic\ Acid}$ (%)	0.00	12.50
S_{CO_2} (%)	3.09	1.53

Commercial catalyst $VOSO_4$

The weight for the glucose-water stock solution was 3.963 g glucose in 220 ml deionized water. The solution was used for two runs.

Table S14: weight of catalyst, reactors and filled in solutions and pH measurements for the commercial catalyst of catalyst screening. Reaction conditions: n(Substrate) = 1 mmol glucose; n(Catalyst) = 0.1 mmol vanadium; V = 10 mL, pN_2 = 20 bar; T = 200 °C; t = 1 h; $V_{stirrer}$ = level 6; Solvent: H_2O.

Catalyst	$m_{catalyst}$ (g)	pH_{before}	$m_{Reactor}$ (g)	$m_{solution}$ (g)	pH_{after}	$m_{solution.after}$ (g)
$VOSO_4$	0.353	3.17	341.340	10.190	2.3	9.985
			340.779	10.174	2.22	9.975
			340.357	10.203	2.22	9.966
			240.547	10.212	2.16	10.079
			240.794	10.185	2.17	10.026
			363.860	10.196	2.24	9.88
			373.522	10.181	2.24	10.005
			336.240	10.176	2.21	10.011
			334.045	10.168	2.26	9.945

Table S15: calculated yields of all detected products for the commercial catalyst of catalyst screening. Reaction conditions: n(Substrate) = 1 mmol glucose; n(Catalyst) = 0.1 mmol vanadium; V = 10 mL, pN_2 = 20 bar; T = 200 °C; t = 1 h; $V_{stirrer}$ = level 6; Solvent: H_2O

Experiment	$VOSO_4$								
$Y_{Glucose}$ (%)	1.82	1.82	1.82	1.82	1.82	1.82	1.82	1.82	2.73
$Y_{Fructose}$ (%)	0.00	0.00	0.00	0.00	0.00	0.00	0.00	0.00	0.00
$Y_{Mannose}$ (%)	0.91	0.91	0.91	0.91	0.91	0.91	0.91	0.91	0.91
Y_{DHA} (%)	0.00	0.00	0.00	0.00	0.00	0.00	0.00	0.00	0.00
$Y_{GlycerAl}$ (%)	0.00	0.00	0.00	0.00	0.00	0.00	0.00	0.00	0.00
$Y_{Pyruvaldehyde}$ (%)	11.82	11.82	11.82	11.82	11.82	11.36	11.82	11.82	11.82
Y_{LA} (%)	30.00	30.45	30.91	30.45	30.45	31.36	30.91	30.91	30.91

Y_{AcetAl} (%)	9.39	9.70	9.39	9.70	9.39	8.48	9.39	8.79	9.09
$Y_{Acetic\ Acid}$ (%)	4.24	4.24	4.55	4.24	4.24	4.24	4.55	4.24	4.24
$Y_{Glycolaldehyde}$ (%)	1.21	1.21	1.21	1.21	1.21	1.21	1.21	1.21	1.52
Y_{GA} (%)	0.00	0.00	0.00	0.00	0.00	0.00	0.00	0.00	0.00
$Y_{5\text{-}HMF}$ (%)	0.00	0.00	0.00	0.00	0.00	0.00	0.00	0.00	0.00
Y_{FA} (%)	3.64	2.73	2.73	2.73	3.64	2.73	2.73	2.73	2.73
Y_{LevA} (%)	4.39	3.94	3.94	3.94	3.79	3.94	3.64	3.64	3.79
$Y_{Glyoxal}$ (%)	0.00	0.00	0.00	0.00	0.00	0.00	0.00	0.00	0.00
$Y_{Succinic\ Acid}$ (%)	0.00	0.00	0.00	0.00	0.00	0.00	0.00	0.00	0.00
Y_{CO_2} (%)	1.85	1.76	1.88	2.09	2.05	2.42	2.43	2.21	2.12
$Y_{Conversion}$ (%)	98.18	98.18	98.18	98.18	98.18	98.18	98.18	98.18	97.27
$Y_{Carbon\ Balance}$ (%)	69.27	68.58	69.16	68.91	69.32	68.48	69.40	68.27	69.84

Table S16: calculated selectivity's of all detected products for the commercial catalyst of catalyst screening. Reaction conditions: n(Substrate) = 1 mmol glucose; n(Catalyst) = 0.1 mmol vanadium; V = 10 mL, pN_2 = 20 bar; T = 200 °C; t = 1 h; $V_{stirrer}$ = level 6; Solvent: H_2O

Experiment	$VOSO_4$								
$S_{Glucose}$ (%)	1.85	1.85	1.85	1.85	1.85	1.85	1.85	1.85	2.80
$S_{Fructose}$ (%)	0.00	0.00	0.00	0.00	0.00	0.00	0.00	0.00	0.00
$S_{Mannose}$ (%)	0.93	0.93	0.93	0.93	0.93	0.93	0.93	0.93	0.93
S_{DHA} (%)	0.00	0.00	0.00	0.00	0.00	0.00	0.00	0.00	0.00
$S_{GlycerAl}$ (%)	0.00	0.00	0.00	0.00	0.00	0.00	0.00	0.00	0.00
$S_{Pyruvaldehyde}$ (%)	12.04	12.04	12.04	12.04	12.04	11.57	12.04	12.04	12.15
S_{LA} (%)	1.23	1.23	1.23	1.23	1.23	1.23	1.23	1.23	1.56
S_{AcetAl} (%)	0.00	0.00	0.00	0.00	0.00	0.00	0.00	0.00	0.00
$S_{Acetic\ Acid}$ (%)	0.00	0.00	0.00	0.00	0.00	0.00	0.00	0.00	0.00
$S_{Glycolaldehyde}$ (%)	30.56	31.02	31.48	31.02	31.02	31.94	31.48	31.48	31.78
S_{GA} (%)	0.00	0.00	0.00	0.00	0.00	0.00	0.00	0.00	0.00
$S_{5\text{-}HMF}$ (%)	4.48	4.01	4.01	4.01	3.86	4.01	3.70	3.70	3.89
S_{FA} (%)	4.32	4.32	4.63	4.32	4.32	4.32	4.63	4.32	4.36
S_{LevA} (%)	9.57	9.88	9.57	9.88	9.57	8.64	9.57	8.95	9.35
$S_{Glyoxal}$ (%)	0.00	0.00	0.00	0.00	0.00	0.00	0.00	0.00	0.00
$S_{Succinic\ Acid}$ (%)	3.70	2.78	2.78	2.78	3.70	2.78	2.78	2.78	2.80
S_{CO_2} (%)	0.00	0.00	0.00	0.00	0.00	0.00	0.00	0.00	0.00

Table S17: weight of catalyst, reactors and filled in solutions and pH measurements for the commercial catalyst of catalyst screening. Reaction conditions: n(Substrate) = 1 mmol glucose; n(Catalyst) = 0.1 mmol vanadium; V = 10 mL, pN_2 = 20 bar; T = 200 °C; t = 1 h; $V_{stirrer}$ = level 6; Solvent: H_2O

Catalyst	$m_{catalyst}$ (g)	pH_{before}	$m_{Reactor}$ (g)	$m_{solution}$ (g)	pH_{after}	$m_{solution.after}$ (g)
$VOSO_4$	0,353	3.17	341.336	10.177	2.21	9.975
			340.759	10.179	2.07	10.069
			340.354	10.176	2.2	10.142
			340.554	10.183	2.17	10.073
			340.758	10.202	2.22	10.06
			336.848	10.186	2.12	10.063
			373.510	10.189	2.18	10.093
			336.239	10.198	2.19	10.168
			334.012	10.178	2.24	10.094

Table S18: calculated yields of all detected products for the commercial catalyst of catalyst screening. Reaction conditions: n(Substrate) = 1 mmol glucose; n(Catalyst) = 0.1 mmol vanadium; V = 10 mL, pN_2 = 20 bar; T = 200 °C; t = 1 h; $V_{stirrer}$ = level 6; Solvent: H_2O

Experiment	$VOSO_4$								
$Y_{Glucose}$ (%)	1.82	1.82	1.82	1.82	1.82	1.82	1.82	1.82	1.82
$Y_{Fructose}$ (%)	0.00	0.00	0.00	0.00	0.00	0.00	0.00	0.00	0.00
$Y_{Mannose}$ (%)	0.91	0.91	0.91	0.91	0.91	0.91	0.91	0.91	0.91
Y_{DHA} (%)	0.00	0.00	0.00	0.00	0.00	0.00	0.00	0.00	0.00
$Y_{GlycerAl}$ (%)	0.00	0.00	0.00	0.00	0.00	0.00	0.00	0.00	0.00
$Y_{Pyruvaldehyde}$ (%)	11.82	11.82	11.36	11.82	12.27	11.82	11.82	11.82	11.82
Y_{LA} (%)	31.36	31.36	30.91	30.91	30.91	30.45	30.91	30.91	30.91
Y_{AcetAl} (%)	9.70	9.09	9.09	9.09	9.39	9.39	9.39	9.09	8.79
$Y_{Acetic\ Acid}$ (%)	4.24	4.24	4.24	4.24	4.24	4.24	4.24	4.24	4.24
$Y_{Glycolaldehyde}$ (%)	1.21	1.21	1.21	1.21	1.21	1.21	1.21	1.21	1.21
Y_{GA} (%)	0.00	0.00	0.00	0.00	0.00	0.00	0.00	0.00	0.00
$Y_{5\text{-}HMF}$ (%)	0.00	0.00	0.00	0.00	0.00	0.00	0.00	0.00	0.00
Y_{FA} (%)	2.73	2.73	2.73	2.73	3.64	2.73	2.73	2.73	3.64
Y_{LevA} (%)	3.94	3.94	3.79	3.94	3.79	3.64	3.64	3.64	3.79
$Y_{Glyoxal}$ (%)	0.00	0.00	0.00	0.00	0.00	0.00	0.00	0.00	0.00
$Y_{Succinic\ Acid}$ (%)	0.00	0.00	0.00	0.00	0.00	0.00	0.00	0.00	0.00
Y_{CO_2} (%)	2.23	1.98	1.47	2.16	2.14	2.86	2.67	2.40	2.25
$Y_{Conversion}$ (%)	98.18	98.18	98.18	98.18	98.18	98.18	98.18	98.18	98.18
$Y_{Carbon\ Balance}$ (%)	69.96	69.10	67.53	68.82	70.32	69.07	69.34	68.77	69.37

Table S19: calculated selectivitys of all detected products for commercial catalyst of catalyst screening. Reaction conditions: n(Substrate) = 1 mmol glucose; n(Catalyst) = 0.1 mmol vanadium; V = 10 mL, pN_2 = 20 bar; T = 200 °C; t = 1 h; $V_{stirrer}$ = level 6; Solvent: H_2O.

Experiment	$VOSO_4$								
$S_{Glucose}$ (%)	1.85	1.85	1.85	1.85	1.85	1.85	1.85	1.85	1.85
$S_{Fructose}$ (%)	0.00	0.00	0.00	0.00	0.00	0.00	0.00	0.00	0.00
$S_{Mannose}$ (%)	0.93	0.93	0.93	0.93	0.93	0.93	0.93	0.93	0.93
S_{DHA} (%)	0.00	0.00	0.00	0.00	0.00	0.00	0.00	0.00	0.00
$S_{GlycerAl}$ (%)	0.00	0.00	0.00	0.00	0.00	0.00	0.00	0.00	0.00
$S_{Pyruvaldehyde}$ (%)	12.04	12.04	11.57	12.04	12.50	12.04	12.04	12.04	12.04
S_{LA} (%)	1.23	1.23	1.23	1.23	1.23	1.23	1.23	1.23	1.23
S_{AcetAl} (%)	0.00	0.00	0.00	0.00	0.00	0.00	0.00	0.00	0.00
$S_{Acetic\ Acid}$ (%)	0.00	0.00	0.00	0.00	0.00	0.00	0.00	0.00	0.00
$S_{Glycolaldehyde}$ (%)	31.94	31.94	31.48	31.48	31.48	31.02	31.48	31.48	31.48
S_{GA} (%)	0.00	0.00	0.00	0.00	0.00	0.00	0.00	0.00	0.00
$S_{5\text{-}HMF}$ (%)	4.01	4.01	3.86	4.01	3.86	3.70	3.70	3.70	3.86
S_{FA} (%)	4.32	4.32	4.32	4.32	4.32	4.32	4.32	4.32	4.32
S_{LevA} (%)	9.88	9.26	9.26	9.26	9.57	9.57	9.57	9.26	8.95
$S_{Glyoxal}$ (%)	0.00	0.00	0.00	0.00	0.00	0.00	0.00	0.00	0.00
$S_{Succinic\ Acid}$ (%)	2.78	2.78	2.78	2.78	3.70	2.78	2.78	2.78	3.70
S_{CO_2} (%)	0.00	0.00	0.00	0.00	0.00	0.00	0.00	0.00	0.00

Unsubstituted Catalyst $H_3PW_{12}O_{40}$

The weight for the glucose-water stock solution was 1.9813 g glucose in 110 ml deionized water.

Table S20: weight of catalyst, reactors and filled in solutions and pH measurements for unsubstituted catalyst of catalyst screening. Reaction conditions: n(Substrate) = 1 mmol glucose; n(Catalyst) = 0.1 mmol vanadium; V = 10 mL, pN_2 = 20 bar; T = 200 °C; t = 1 h; $V_{stirrer}$ = level 6; Solvent: H_2O

Catalyst	$m_{catalyst}$ (g)	pH_{before}	$m_{Reactor}$ (g)	$m_{solution}$ (g)	pH_{after}	$m_{solution.after}$ (g)
$H_3PW_{12}O_{40}$	0.865	1.313	341.263	9.68	1.391	10.00
$H_3PW_{12}O_{40}$			340.698	10.81	1.379	10.16
$H_3PW_{12}O_{40}$			340.250	10.25	1.395	10.13
Blank	-	5.653	340.520	9.99	2.675	9.90

Table S21: calculated yields of all detected products for the unsubstituted catalyst of catalyst screening. Reaction conditions: n(Substrate) = 1 mmol glucose; n(Catalyst) = 0.1 mmol vanadium; V = 10 mL, pN_2 = 20 bar; T = 200 °C; t = 1 h; $V_{stirrer}$ = level 6; Solvent: H_2O

Experiment	$H_3PW_{12}O_{40}$	$H_3PW_{12}O_{40}$	$H_3PW_{12}O_{40}$	Blank
$Y_{Glucose}$ (%)	9.17	6.42	6.42	52.29
$Y_{Fructose}$ (%)	0.00	0.00	0.00	0.00
$Y_{Mannose}$ (%)	4.59	3.67	2.75	0.00
Y_{DHA} (%)	1.83	1.38	1.38	1.83
$Y_{GlycerAl}$ (%)	0.00	0.00	0.00	0.92
$Y_{Pyruvaldehyde}$ (%)	10.55	10.09	10.09	0.00
Y_{LA} (%)	0.00	0.00	0.00	0.00
Y_{AcetAl} (%)	0.00	0.00	0.00	4.89
$Y_{Acetic Acid}$ (%)	5.50	4.59	4.59	3.36
$Y_{Glycolaldehyde}$ (%)	0.31	0.61	0.31	1.22
Y_{GA} (%)	7.03	7.34	7.03	0.00
Y_{5-HMF} (%)	0.00	0.00	0.00	0.00
Y_{FA} (%)	8.26	7.34	6.42	29.36
Y_{LevA} (%)	9.33	9.63	7.80	1.68
$Y_{Glyoxal}$ (%)	34.40	32.87	33.64	0.76
$Y_{Succinic Acid}$ (%)	0.00	0.00	0.00	6.12
Y_{CO_2} (%)	0.44	1.24	1.29	0.44
$Y_{Conversion}$ (%)	90.83	93.58	93.58	47.71
$Y_{Carbon Balance}$ (%)	91.42	85.19	81.71	102.89

Table S22: calculated selectivity's of all detected products for the unsubstituted catalyst of catalyst screening. Reaction conditions: n(Substrate) = 1 mmol glucose; n(Catalyst) = 0.1 mmol vanadium; V = 10 mL, pN_2 = 20 bar; T = 200 °C; t = 1 h; $V_{stirrer}$ = level 6; Solvent: H_2O

Experiment	$H_3PW_{12}O_{40}$	$H_3PW_{12}O_{40}$	$H_3PW_{12}O_{40}$	Blank
$S_{Glucose}$ (%)	10.10	6.86	6.86	109.62
$S_{Fructose}$ (%)	0.00	0.00	0.00	0.00
$S_{Mannose}$ (%)	5.05	3.92	2.94	0.00
S_{DHA} (%)	0.00	0.00	0.00	0.00
$S_{GlycerAl}$ (%)	0.00	0.00	0.00	1.92
$S_{Pyruvaldehyde}$ (%)	11.62	10.78	10.78	0.00
S_{LA} (%)	0.34	0.65	0.33	2.56
S_{AcetAl} (%)	7.74	7.84	7.52	0.00
$S_{Acetic\ Acid}$ (%)	0.00	0.00	0.00	12.82
$S_{Glycolaldehyde}$ (%)	0.00	0.00	0.00	0.00
S_{GA} (%)	2.02	1.47	1.47	3.85
$S_{5\text{-}HMF}$ (%)	10.27	10.29	8.33	3.53
S_{FA} (%)	6.06	4.90	4.90	7.05
S_{LevA} (%)	0.00	0.00	0.00	10.26
$S_{Glyoxal}$ (%)	37.88	35.13	35.95	1.60
$S_{Succinic\ Acid}$ (%)	9.09	7.84	6.86	61.54
S_{CO_2} (%)	0.00	0.00	0.00	0.00

Titration

To analyse catalyst acidity via titration, 20 ml of 0.005 M aqueous POM solutions were prepared. These solutions were titrated with 0.2 M Titripur® sodium hydroxide solution (LOT: HC43967740; Product Number: 1.09140.1000 by Merck KGaA) using a TitroLine 7800 titration automat from SI Analytics equipped with a pH A 162 electrode and a TetraCon 925 conductivity electrode.

The measurements were performed using individual dynamic mode with an initial waiting time of 10 s. Dosing speed was reduced to 25 %. The maximum titration volume was set to 20 ml and the stirrer was used on setting 3.

Figure S15 shows the four titration and conductivity curves for the protonated catalysts.

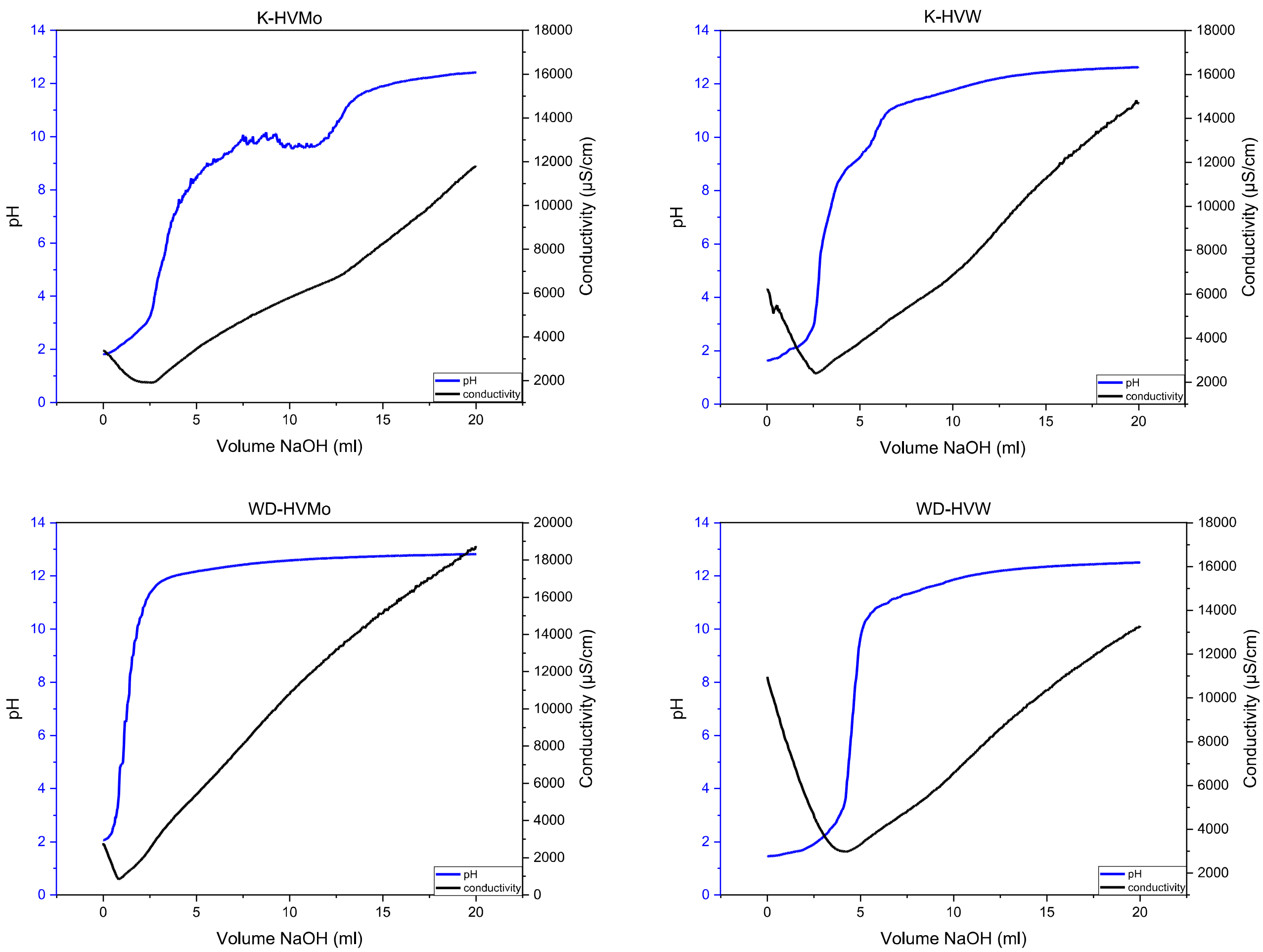

Figure S15: Titration curves and conductivity for all used protonated catalysts.

Electrochemistry

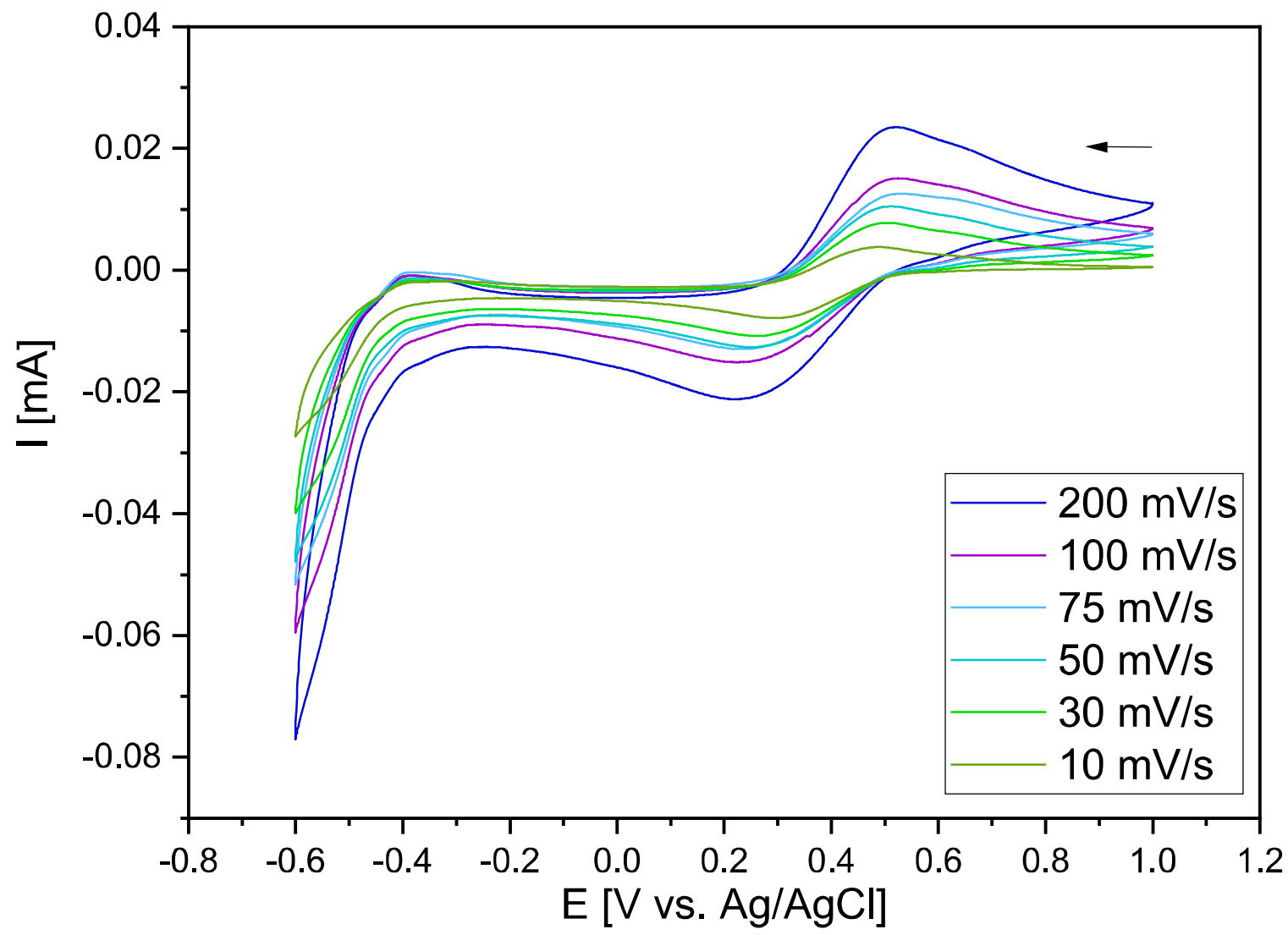

Figure S16: Cyclic voltammograms of $Na_6PV_3W_9O_{40}$ (1 mmol/L) in H_2O (HCl as supporting electrolyte) at pH 1; different scan rates (arrow indicates scanning direction).

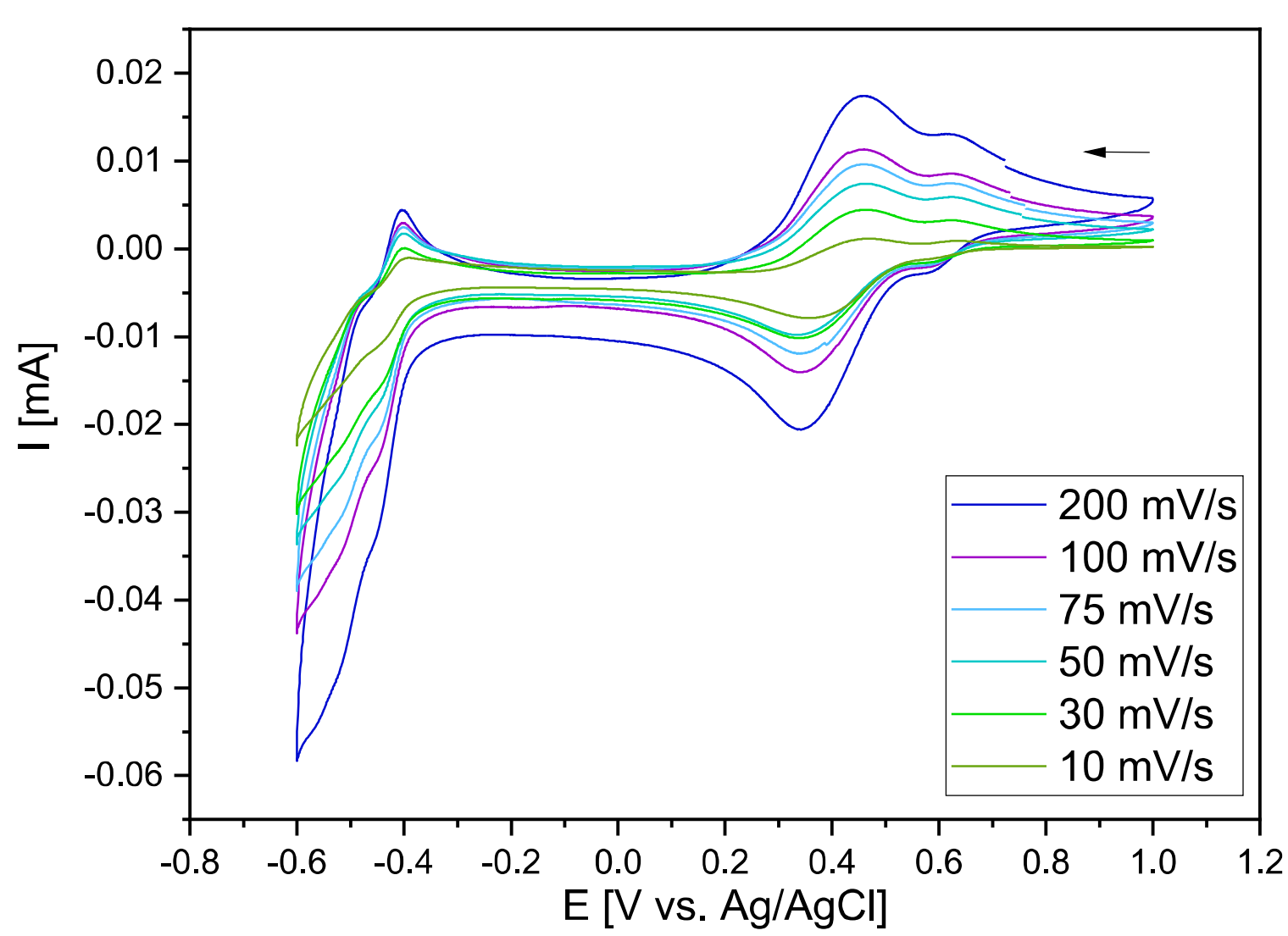

Figure S17: Cyclic voltammograms of $H_6PV_3W_9O_{40}$ (1 mmol/L) in H_2O (HCl as supporting electrolyte) at pH 1; different scan rates (arrow indicates scanning direction).

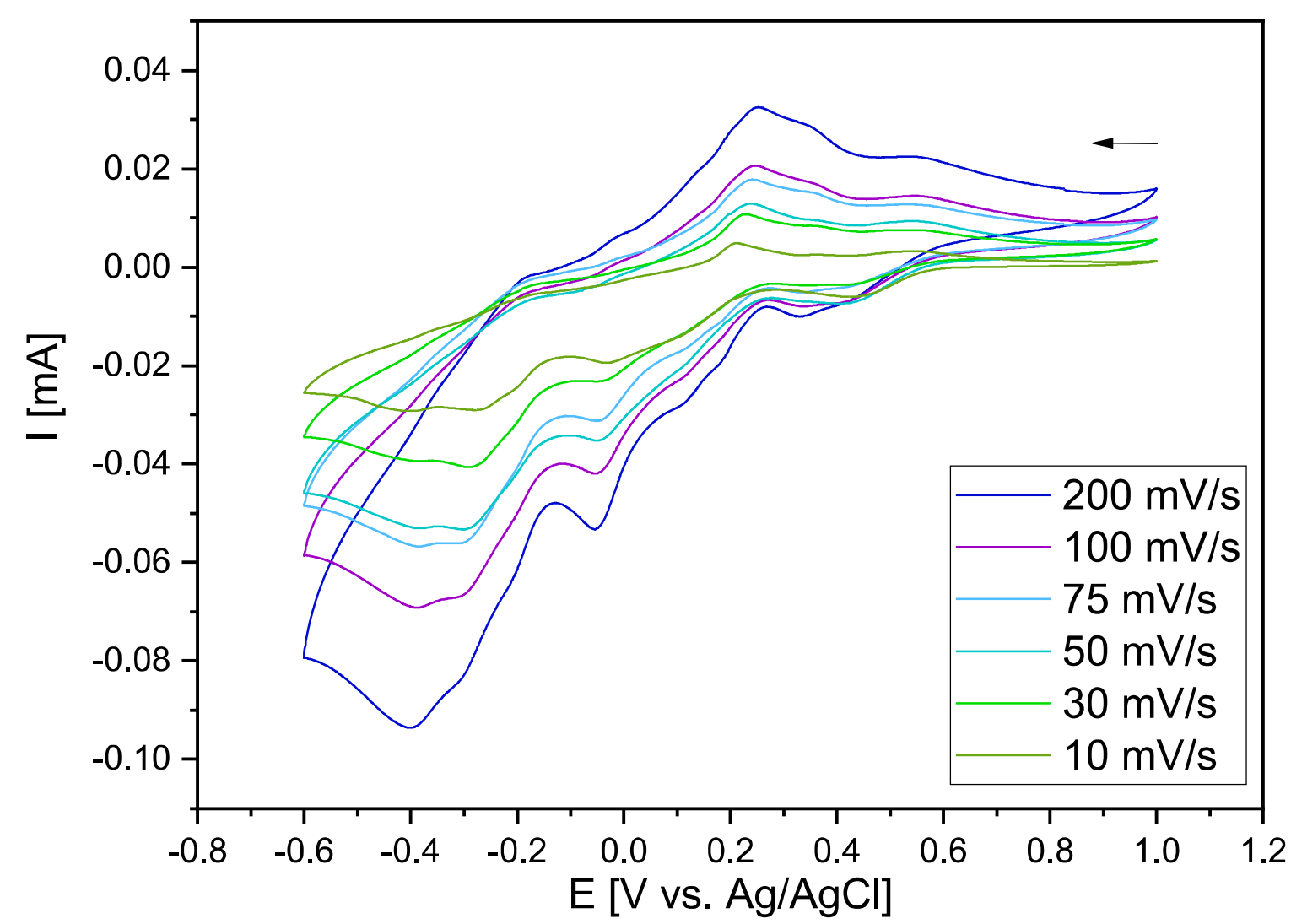

Figure S18: Cyclic voltammograms of $Na_6PV_3Mo_9O_{40}$ (1 mmol/L) in H_2O (HCl as supporting electrolyte) at pH 1; different scan rates (arrow indicates scanning direction).

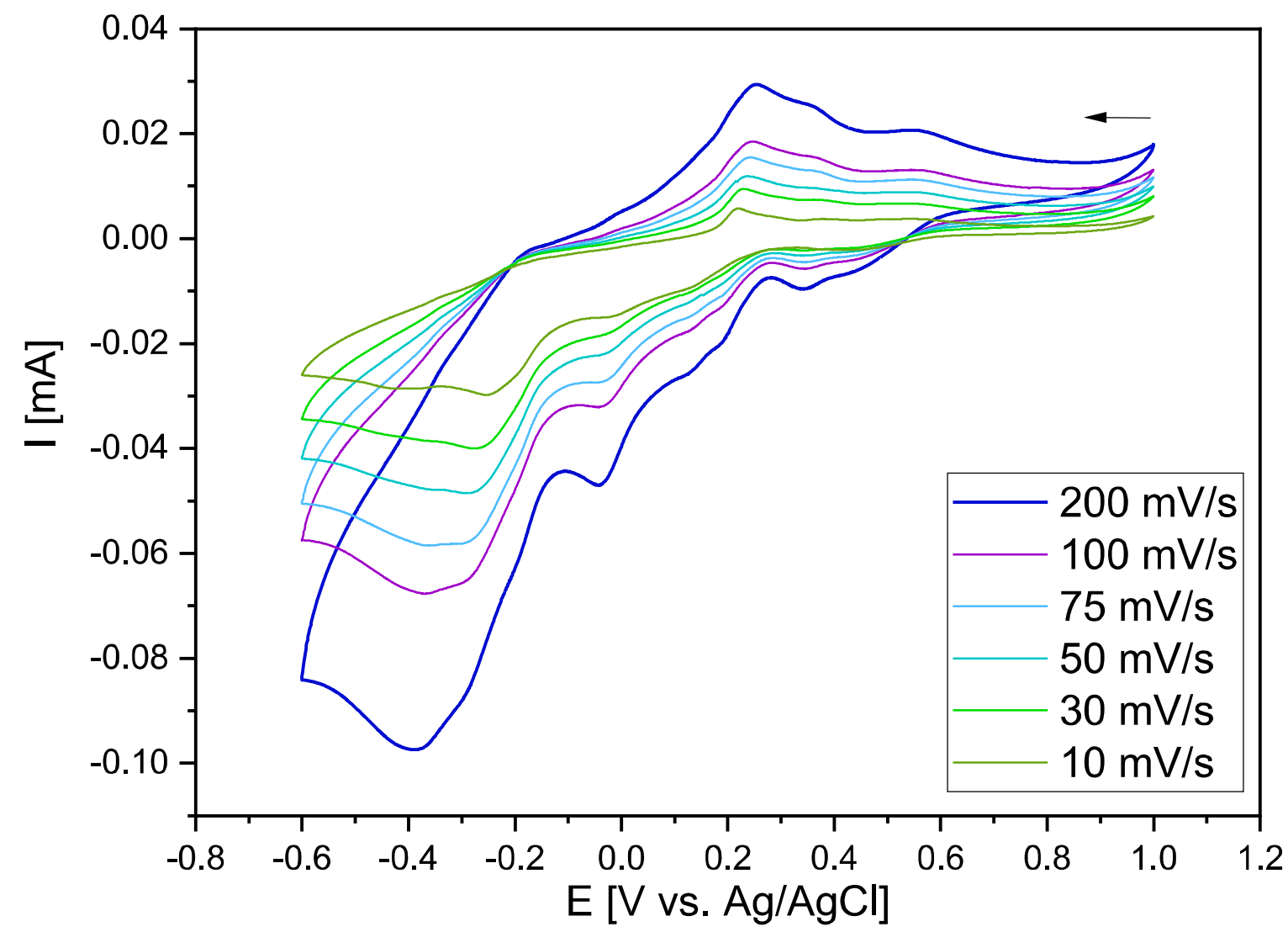

Figure S19: Cyclic voltammograms of $H_6PV_4Mo_8O_{40}$ (1 mmol/L) in H_2O (HCl as supporting electrolyte) at pH 1; different scan rates (arrow indicates scanning direction).

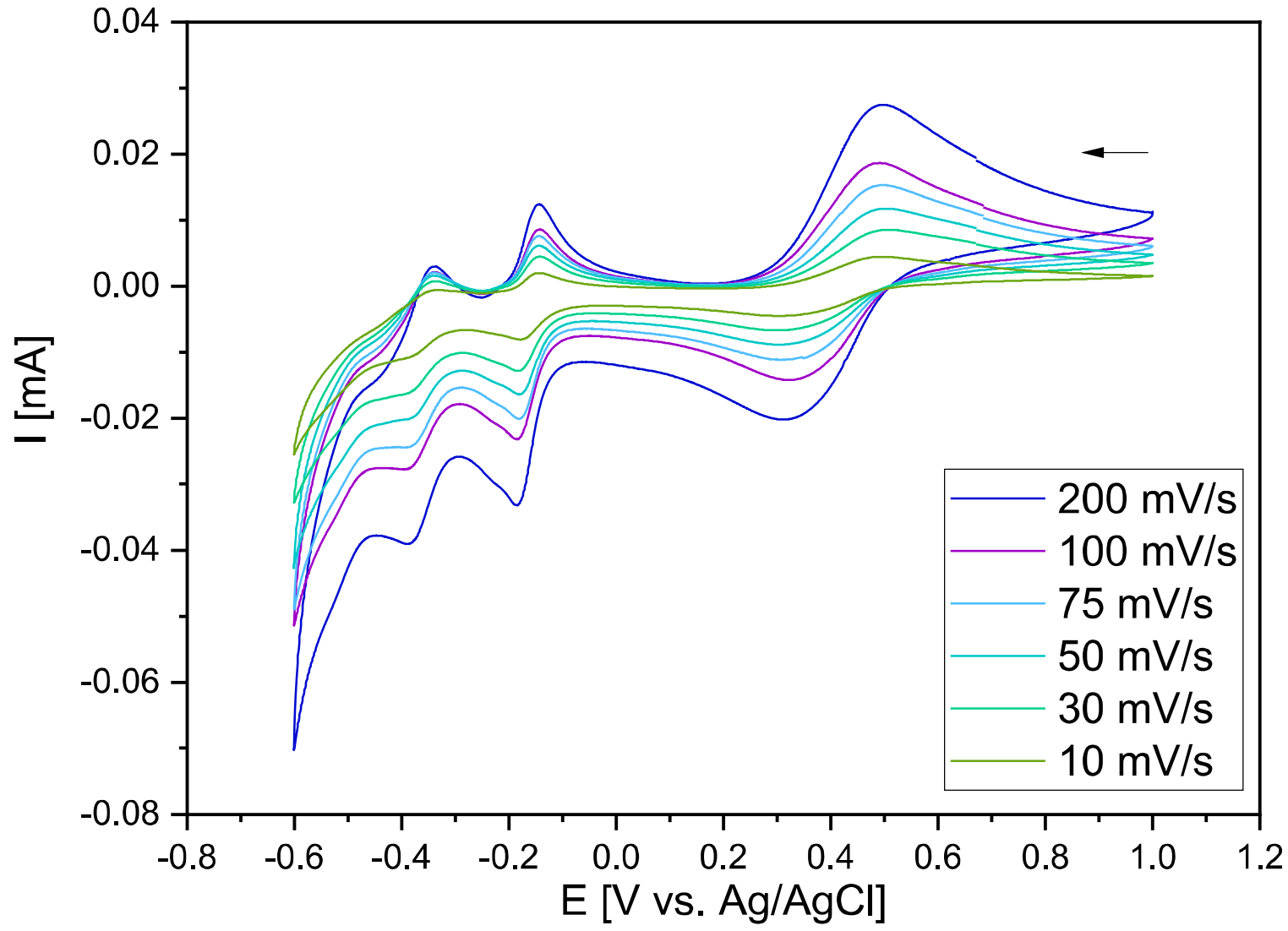

Figure S20: Cyclic voltammograms of $Na_8HP_2V_3W_{15}O_{62}$ (1 mmol/L) in H_2O (HCl as supporting electrolyte) at pH 1; different scan rates (arrow indicates scanning direction).

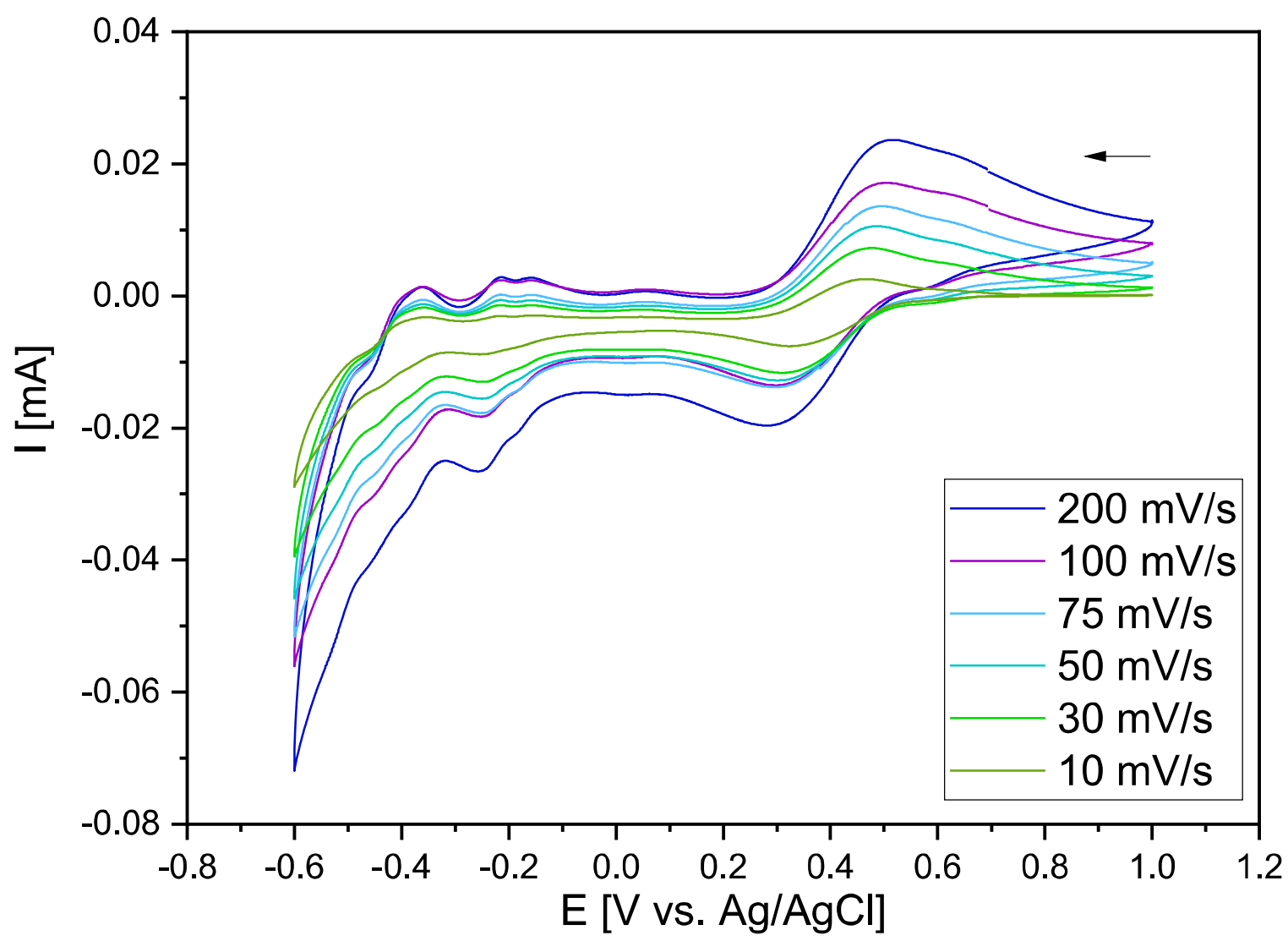

Figure S21: Cyclic voltammograms of $H_9P_2V_3W_{15}O_{62}$ (1 mmol/L) in H_2O (HCl as supporting electrolyte) at pH 1; different scan rates (arrow indicates scanning direction).

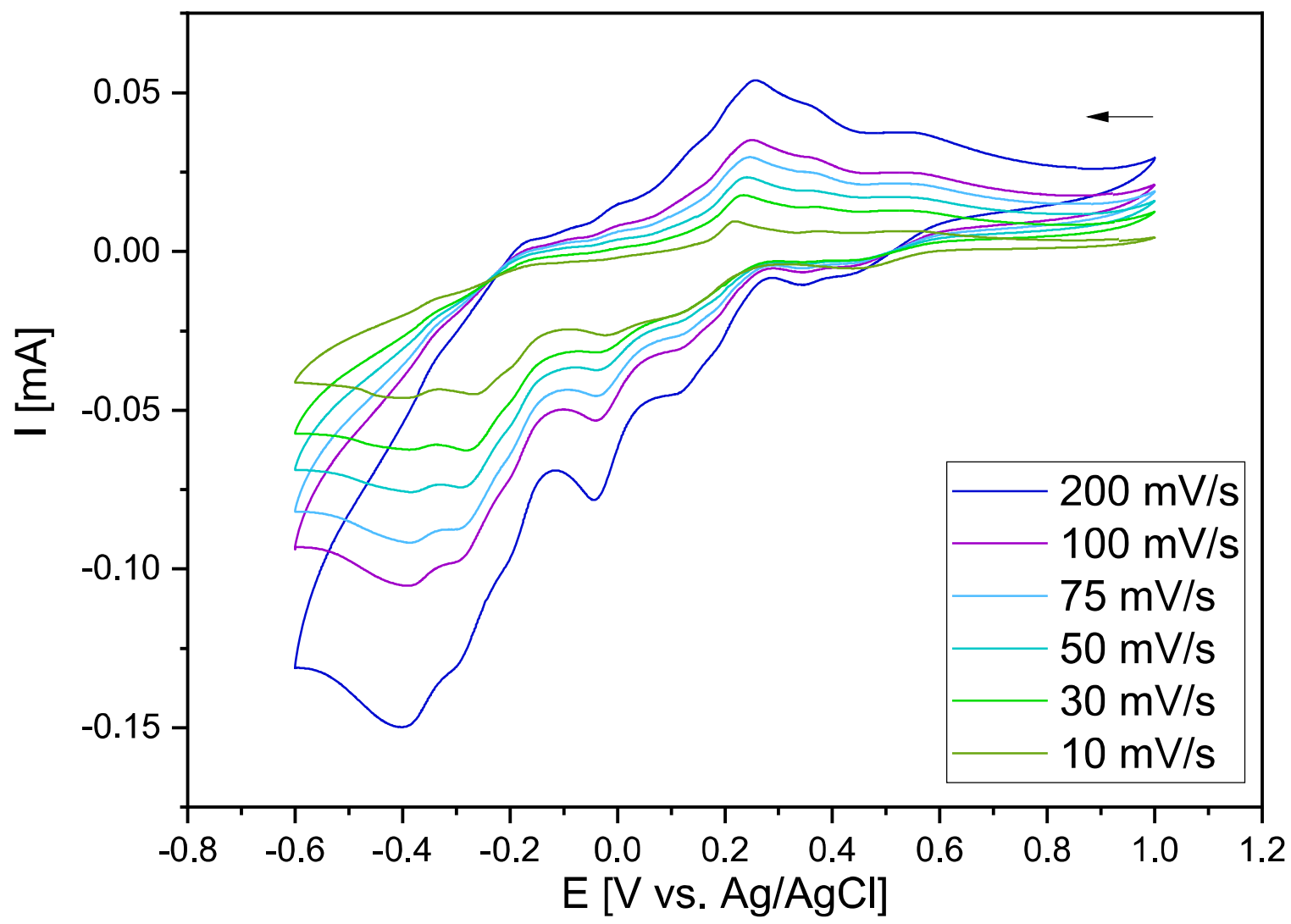

Figure S22: Cyclic voltammograms of $Na_7H_2P_2V_3Mo_{15}O_{62}$ (1 mmol/L) in H_2O (HCl as supporting electrolyte) at pH 1; different scan rates (arrow indicates scanning direction).

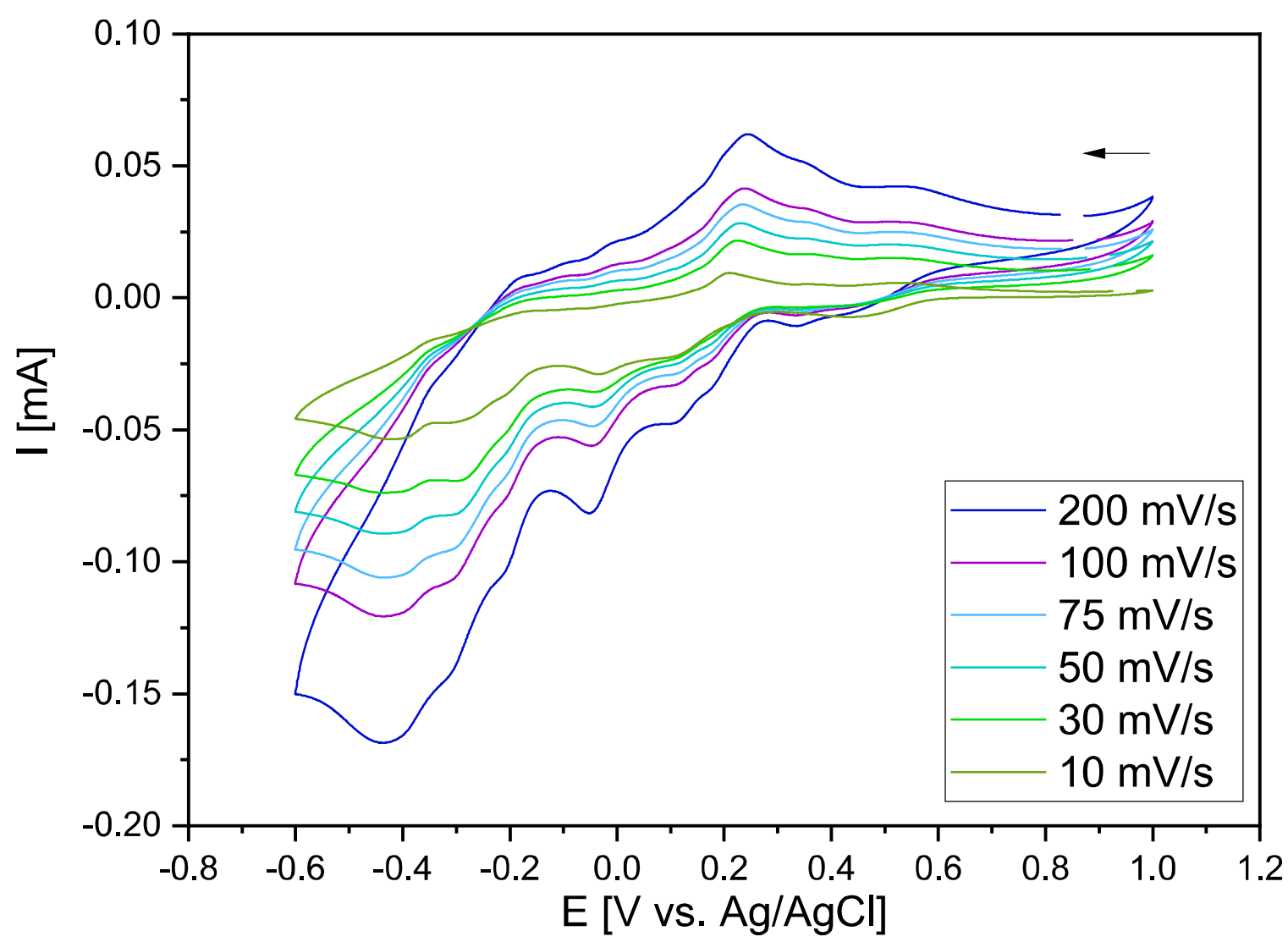

Figure S23: Cyclic voltammograms of $H_9P_2V_3Mo_{15}O_{62}$ (1 mmol/L) in H_2O (HCl as supporting electrolyte) at pH 1; different scan rates (arrow indicates scanning direction).

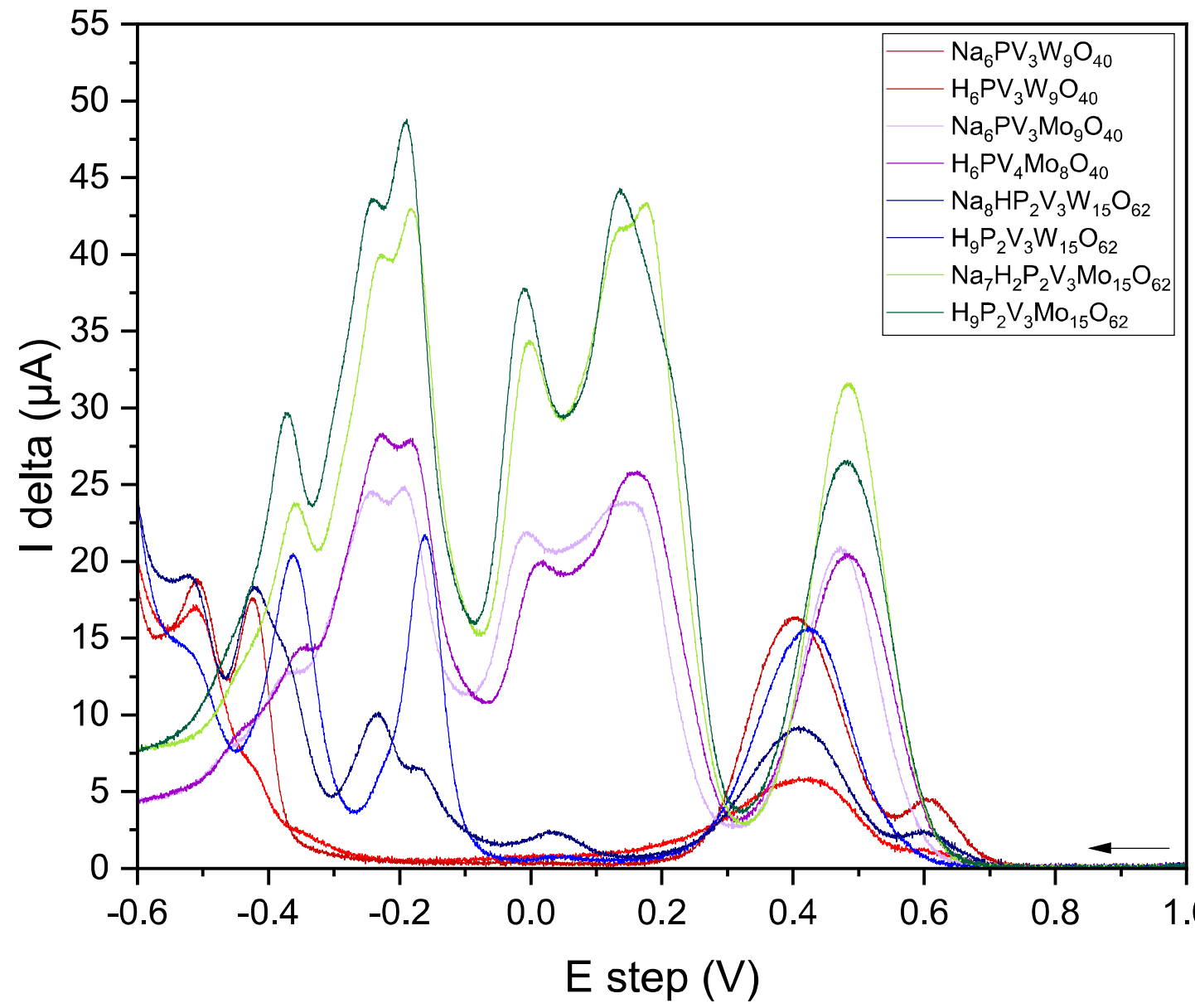

Figure S24: Comparison of square-wave voltammograms of POMs (1 mmol/L) in H_2O, using HCl as supporting electrolyte (pH=1); scan rate 5 mV/s (arrow indicates scanning direction).

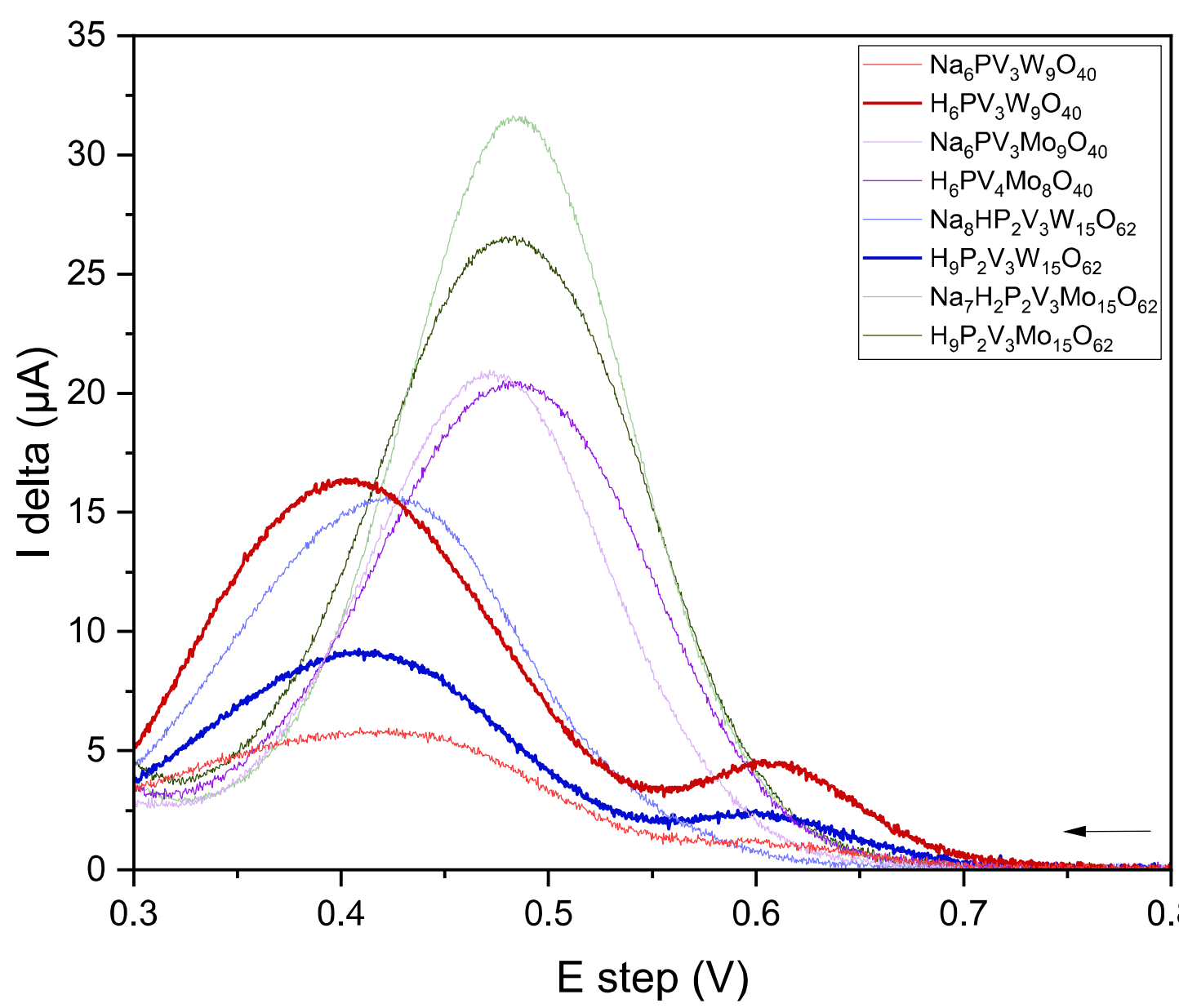

Figure S25: Enlarged area (0.3 –0.8 V) of the Comparison of square-wave voltammograms of POMs (1 mmol/L) in H_2O, using HCl as supporting electrolyte (pH=1); scan rate 5 mV/s (arrow indicates scanning direction).

Pathway Investigations

All diagrams in the main manuscript are shown in the colour code: yellow for the substrate, green for the pathway to lactic acid, red for the pathway via 5-HMF, purple for the pathway to glycolic acid, and blue for the subsequent pathways of lactic acid. Values are the average of three experiments. The error bars represent the standard deviation. All reactions were performed with the same conditions of 200°C for one hour, a fructose concentration 0.1 mol L^{-1}, a dihydroxyacetone/glyceraldehyde/lactic acid concentration 0.2 mol L^{-1}, pyruvaldehyde concertation 0.15 mol L^{-1} and a catalyst concentration of 0.01 mol L^{-1} (based on the vanadium content).

Fructose

The stock solution for the catalyst was 0.27782 g catalyst and 0.8102 g fructose in 45 ml deionized water. The pH of the solution was 1.986.

Table S23: weight of catalyst, reactors and filled in solutions and pH measurements for the fructose in the pathway investigation. Reaction conditions: n(Substrate) = 1 mmol fructose; n(Catalyst) = 0.1 mmol vanadium; V = 10 mL, pN_2 = 20 bar; T = 200 °C; t = 1 h; $V_{stirrer}$ = level 6; Solvent: H_2O.

Catalyst	$m_{catalyst}$ (g)	pH_{before}	$m_{Reactor}$ (g)	$m_{solution}$ (g)	pH_{after}	$m_{solution.after}$ (g)
K-HVW	-	-	340.309	10.260	2.165	11.129
Blank	-	5.888	340.345	10.182	2.634	9.976
K-HVW	-	-	340.737	10.261	2.139	10.241
K-HVW	-	-	336.189	10.260	2.165	10.318

Table S24: calculated yields of all detected products for the fructose in the pathway investigation. Reaction conditions: n(Substrate) = 1 mmol fructose; n(Catalyst) = 0.1 mmol vanadium; V = 10 mL, pN_2 = 20 bar; T = 200 °C; t = 1 h; $V_{stirrer}$ = level 6; Solvent: H_2O.

Experiment	K-HVW	Blank	K-HVW	K-HVW
$Y_{Glucose}$ (%)	2.30	3.45	2.30	2.30
$Y_{Fructose}$ (%)	0.00	0.00	0.00	0.00
$Y_{Mannose}$ (%)	1.15	2.30	1.15	1.15
Y_{DHA} (%)	0.00	0.00	0.00	0.00
$Y_{GlycerAl}$ (%)	2.30	0.00	2.30	2.30
$Y_{Pyruvaldehyde}$ (%)	16.67	0.00	17.24	16.67
Y_{LA} (%)	24.14	0.00	22.99	24.14
Y_{AcetAl} (%)	0.00	6.90	0.00	0.00
$Y_{Acetic\ Acid}$ (%)	4.21	4.60	4.21	4.21
$Y_{Glycolaldehyde}$ (%)	0.00	0.00	0.00	0.00
Y_{GA} (%)	0.00	0.00	0.00	0.00
$Y_{5\text{-}HMF}$ (%)	0.00	41.38	2.30	2.30
Y_{FA} (%)	4.98	1.53	4.79	4.79
Y_{LevA} (%)	13.41	3.83	13.41	12.45
$Y_{Glyoxal}$ (%)	0.00	0.00	0.00	0.00
$Y_{Succinic\ Acid}$ (%)	0.00	0.00	0.00	0.00
Y_{CO_2} (%)	1.92	1.52	1.74	2.05
$Y_{Conversion}$ (%)	1.92	1.52	1.74	100.00
$Y_{Carbon\ Balance}$ (%)	1.92	1.52	1.74	72.36

Table S25: calculated selectivity's of all detected products for the fructose in the pathway investigation. Reaction conditions: n(Substrate) = 1 mmol fructose; n(Catalyst) = 0.1 mmol vanadium; V = 10 mL, pN_2 = 20 bar; T = 200 °C; t = 1 h; $V_{stirrer}$ = level 6; Solvent: H_2O.

Experiment	K-HVW	Blank	K-HVW	K-HVW
$S_{Glucose}$ (%)	2.30	3.45	2.30	2.30
$S_{Fructose}$ (%)	0.00	0.00	0.00	0.00
$S_{Mannose}$ (%)	1.15	2.30	1.15	1.15
S_{DHA} (%)	0.00	0.00	0.00	0.00
$S_{GlycerAl}$ (%)	2.30	0.00	2.30	2.30
$S_{Pyruvaldehyde}$ (%)	16.67	0.00	17.24	16.67
S_{LA} (%)	24.14	0.00	22.99	24.14
S_{AcetAl} (%)	0.00	6.90	0.00	0.00
$S_{Acetic\ Acid}$ (%)	4.21	4.60	4.21	4.21
$S_{Glycolaldehyde}$ (%)	0.00	0.00	0.00	0.00
S_{GA} (%)	0.00	0.00	0.00	0.00
$S_{5\text{-}HMF}$ (%)	0.00	41.38	2.30	2.30
S_{FA} (%)	4.98	1.53	4.79	4.79
S_{LevA} (%)	13.41	3.83	13.41	12.45
$S_{Glyoxal}$ (%)	0.00	0.00	0.00	0.00
$S_{Succinic\ Acid}$ (%)	0.00	0.00	0.00	0.00
S_{CO_2} (%)	1.92	1.52	1.74	2.05

Dihydroxyacetone

The stock solution for the catalyst was 0.2774 g catalyst and 0.8105 g dihydroxyacetone in 45 ml deionized water. The pH of the solution was 1.979.

Table S26: weight of catalyst, reactors and filled in solutions and pH measurements for the dihydroxyacetone in the pathway investigation. Reaction conditions: n(Substrate) = 2 mmol dihydroxyacteone; n(Catalyst) = 0.1 mmol vanadium; V = 10 mL, pN_2 = 20 bar; T = 200 °C; t = 1 h; $V_{stirrer}$ = level 6; Solvent: H_2O.

Catalyst	$m_{catalyst}$ (g)	pH_{before}	$m_{Reactor}$ (g)	$m_{solution}$ (g)	pH_{after}	$m_{solution.after}$ (g)
K-HVW	-	-	340.724	10.263	2.058	10.086
K-HVW	-	-	340.551	10.263	2.035	10.063
Blank	-	4.526	340.800	10.170	2.524	10.045
K-HVW	-	-	336.843	10.263	2.044	10.097

Table S27: calculated yields of all detected products for the dihydroxyacetone in the pathway investigation. Reaction conditions: n(Substrate) = 2 mmol dihydroxyacteone; n(Catalyst) = 0.1 mmol vanadium; V = 10 mL, pN_2 = 20 bar; T = 200 °C; t = 1 h; $V_{stirrer}$ = level 6; Solvent: H_2O.

Experiment	K-HVW	K-HVW	Blank	K-HVW
$Y_{Glucose}$ (%)	2.14	2.14	0.00	2.14
$Y_{Fructose}$ (%)	0.00	0.00	0.00	0.00
$Y_{Mannose}$ (%)	0.00	0.00	1.07	1.07
Y_{DHA} (%)	0.00	0.00	0.00	0.00
$Y_{GlycerAl}$ (%)	0.00	0.00	0.00	0.00
$Y_{Pyruvaldehyde}$ (%)	0.00	0.00	14.97	0.00
Y_{LA} (%)	68.45	68.98	0.00	68.45
Y_{AcetAl} (%)	5.35	5.70	5.70	4.99
$Y_{Acetic\ Acid}$ (%)	5.35	5.35	8.20	5.35
$Y_{Glycolaldehyde}$ (%)	0.00	0.00	0.00	0.00
Y_{GA} (%)	0.00	0.00	9.75	0.00
$Y_{5\text{-}HMF}$ (%)	0.00	0.00	0.00	0.00
Y_{FA} (%)	1.96	1.96	1.78	1.96
Y_{LevA} (%)	0.89	1.78	2.67	0.89
$Y_{Glyoxal}$ (%)	0.00	0.00	0.00	0.00
$Y_{Succinic\ Acid}$ (%)	0.00	0.00	0.00	0.00
Y_{CO_2} (%)	1.78	0.00	3.44	1.93
$Y_{Conversion}$ (%)	100.00	100.00	100.00	100.00
$Y_{Carbon\ Balance}$ (%)	85.92	85.92	47.59	86.78

Table S28: calculated selectivity's of all detected products for the dihydroxyacetone in the pathway investigation. Reaction conditions: n(Substrate) = 2 mmol dihydroxyacteone; n(Catalyst) = 0.1 mmol vanadium; V = 10 mL, pN_2 = 20 bar; T = 200 °C; t = 1 h; $V_{stirrer}$ = level 6; Solvent: H_2O.

Experiment	K-HVW	K-HVW	Blank	K-HVW
$S_{Glucose}$ (%)	2.14	2.14	0.00	2.14
$S_{Fructose}$ (%)	0.00	0.00	0.00	0.00
$S_{Mannose}$ (%)	0.00	0.00	1.07	1.07
S_{DHA} (%)	0.00	0.00	0.00	0.00
$S_{GlycerAl}$ (%)	0.00	0.00	0.00	0.00
$S_{Pyruvaldehyde}$ (%)	0.00	0.00	14.97	0.00
S_{LA} (%)	68.45	68.98	0.00	68.45
S_{AcetAl} (%)	5.35	5.70	5.70	4.99
$S_{Acetic\ Acid}$ (%)	5.35	5.35	8.20	5.35
$S_{Glycolaldehyde}$ (%)	0.00	0.00	0.00	0.00
S_{GA} (%)	0.00	0.00	9.75	0.00
$S_{5\text{-}HMF}$ (%)	0.00	0.00	0.00	0.00
S_{FA} (%)	1.96	1.96	1.78	1.96
S_{LevA} (%)	0.89	1.78	2.67	0.89
$S_{Glyoxal}$ (%)	0.00	0.00	0.00	0.00
$S_{Succinic\ Acid}$ (%)	0.00	0.00	0.00	0.00
S_{CO_2} (%)	1.78	0.00	3.44	1.93

Glyceraldehyde

The stock solution for the catalyst was 0.2778 g catalyst and 0.8106 g glyceraldehyde in 45 ml deionized water. The pH of the solution was 1.964.

Table S29: weight of catalyst, reactors and filled in solutions and pH measurements for the glyceraldehyde in the pathway investigation. Reaction conditions: n(Substrate) = 2 mmol glyceraldehyde; n(Catalyst) = 0.1 mmol vanadium; V = 10 mL, pN_2 = 20 bar; T = 200 °C; t = 1 h; $V_{stirrer}$ = level 6; Solvent: H_2O.

Catalyst	$m_{catalyst}$ (g)	pH_{before}	$m_{Reactor}$ (g)	$m_{solution}$ (g)	pH_{after}	$m_{solution.after}$ (g)
K-HVW	-	-	341.341	10.266	2.045	10.089
K-HVW	-	-	340.361	10.266	2.054	10.084
Blank	-	7.640	336.215	10.177	2.510	10.065
K-HVW	-	-	334.018	10.265	2.063	10.086

Table S30: calculated yields of all detected products for the glyceraldehyde in the pathway investigation. Reaction conditions: n(Substrate) = 2 mmol glyceraldehyde; n(Catalyst) = 0.1 mmol vanadium; V = 10 mL, pN_2 = 20 bar; T = 200 °C; t = 1 h; $V_{stirrer}$ = level 6; Solvent: H_2O.

Experiment	K-HVW	K-HVW	Blank	K-HVW
$Y_{Glucose}$ (%)	2.30	2.30	0.00	2.30
$Y_{Fructose}$ (%)	0.00	0.00	0.00	0.00
$Y_{Mannose}$ (%)	0.00	0.00	2.30	1.15
Y_{DHA} (%)	0.00	0.00	1.72	0.00
$Y_{GlycerAl}$ (%)	0.00	0.00	0.00	0.00
$Y_{Pyruvaldehyde}$ (%)	0.00	0.00	15.52	0.00
Y_{LA} (%)	68.39	68.39	0.00	67.82
Y_{AcetAl} (%)	6.13	6.13	8.05	5.75
$Y_{Acetic\ Acid}$ (%)	5.75	5.75	9.20	5.75
$Y_{Glycolaldehyde}$ (%)	0.00	0.00	0.00	0.00
Y_{GA} (%)	0.00	0.00	10.71	0.00
Y_{5-HMF} (%)	0.00	0.00	3.45	0.00
Y_{FA} (%)	2.49	2.49	2.30	2.49
Y_{LevA} (%)	1.92	1.92	2.87	1.92
$Y_{Glyoxal}$ (%)	0.00	0.00	0.00	0.00
$Y_{Succinic\ Acid}$ (%)	0.00	0.00	0.77	0.00
Y_{CO_2} (%)	2.03	2.12	3.97	2.22
$Y_{Conversion}$ (%)	100.00	100.00	100.00	100.00
$Y_{Carbon\ Balance}$ (%)	89.00	89.09	60.85	89.39

Table S31: calculated selectivity's of all detected products for the glyceraldehyde in the pathway investigation. Reaction conditions: n(Substrate) = 2 mmol glyceraldehyde; n(Catalyst) = 0.1 mmol vanadium; V = 10 mL, pN_2 = 20 bar; T = 200 °C; t = 1 h; $V_{stirrer}$ = level 6; Solvent: H_2O.

Experiment	K-HVW	K-HVW	Blank	K-HVW
$S_{Glucose}$ (%)	2.30	2.30	0.00	2.30
$S_{Fructose}$ (%)	0.00	0.00	0.00	0.00
$S_{Mannose}$ (%)	0.00	0.00	2.30	1.15
S_{DHA} (%)	0.00	0.00	1.72	0.00
$S_{GlycerAl}$ (%)	0.00	0.00	0.00	0.00
$S_{Pyruvaldehyde}$ (%)	0.00	0.00	15.52	0.00
S_{LA} (%)	68.39	68.39	0.00	67.82
S_{AcetAl} (%)	6.13	6.13	8.05	5.75
$S_{Acetic\ Acid}$ (%)	5.75	5.75	9.20	5.75
$S_{Glycolaldehyde}$ (%)	0.00	0.00	0.00	0.00
S_{GA} (%)	0.00	0.00	10.71	0.00
$S_{5\text{-}HMF}$ (%)	0.00	0.00	3.45	0.00
S_{FA} (%)	2.49	2.49	2.30	2.49
S_{LevA} (%)	1.92	1.92	2.87	1.92
$S_{Glyoxal}$ (%)	0.00	0.00	0.00	0.00
$S_{Succinic\ Acid}$ (%)	0.00	0.00	0.77	0.00
S_{CO_2} (%)	2.03	2.12	3.97	2.22

Pyruvaldehyde

The stock solution for the catalyst was 0.2774 g catalyst and 1.6314 g pyruvaldehyde in 45 ml deionized water. The pH of the solution was 2.085.

Table S32: weight of catalyst, reactors and filled in solutions and pH measurements for the pyruvaldehyde in the pathway investigation. Reaction conditions: n(Substrate) =1.5 mmol pyruvaldehyde; n(Catalyst) = 0.1 mmol vanadium; V = 10 mL, pN_2 = 20 bar; T = 200 °C; t = 1 h; $V_{stirrer}$ = level 6; Solvent: H_2O.

Catalyst	$m_{catalyst}$ (g)	pH_{before}	$m_{Reactor}$ (g)	$m_{solution}$ (g)	pH_{after}	$m_{solution.after}$ (g)
K-HVW	-	-	341.319	10.442	2.040	10.432
Blank	-	2.989	340.346	10.360	2.615	10.510
K-HVW	-	-	340.539	10.442	2.047	10.370
K-HVW	-	-	340.757	10.442	2.045	10.345

Table S33: calculated yields of all detected products for the pyruvaldehyde in the pathway investigation. Reaction conditions: n(Substrate) = 1.5 mmol pyruvaldehyde; n(Catalyst) = 0 .1 mmol vanadium; V = 10 mL, pN_2 = 20 bar; T = 200 °C; t = 1 h; $V_{stirrer}$ = level 6; Solvent: H_2O.

Experiment	K-HVW	Blank	K-HVW	K-HVW
$Y_{Glucose}$ (%)	1.93	0.00	1.93	1.93
$Y_{Fructose}$ (%)	0.00	0.00	0.00	0.00
$Y_{Mannose}$ (%)	0.00	0.97	0.00	0.00
Y_{DHA} (%)	0.00	1.45	0.00	1.45
$Y_{GlycerAl}$ (%)	0.00	0.00	0.00	0.00
$Y_{Pyruvaldehyde}$ (%)	0.00	13.05	0.00	0.00
Y_{LA} (%)	59.92	0.00	60.41	60.89
Y_{AcetAl} (%)	4.83	8.38	4.83	4.83
$Y_{Acetic\ Acid}$ (%)	12.56	13.21	12.56	12.56
$Y_{Glycolaldehyde}$ (%)	0.00	0.00	0.00	0.00
Y_{GA} (%)	0.00	7.89	0.00	0.00
$Y_{5\text{-}HMF}$ (%)	0.00	0.00	0.00	0.00
Y_{FA} (%)	1.77	1.61	1.77	2.09
Y_{LevA} (%)	1.61	1.61	1.61	1.61
$Y_{Glyoxal}$ (%)	0.00	0.00	0.00	0.00
$Y_{Succinic\ Acid}$ (%)	0.00	0.00	0.00	0.00
Y_{CO_2} (%)	0.67	2.76	0.65	0.66
$Y_{Conversion}$ (%)	100.00	86.95	100.00	100.00
$Y_{Carbon\ Balance}$ (%)	83.30	50.92	83.77	86.03

Table S34: calculated selectivity's of all detected products for the pyruvaldehyde in the pathway investigation. Reaction conditions: n(Substrate) = 1.5 mmol pyruvaldehyde; n(Catalyst) = 0 .1 mmol vanadium; V = 10 mL, pN_2 = 20 bar; T = 200 °C; t = 1 h; $V_{stirrer}$ = level 6; Solvent: H_2O.

Experiment	K-HVW	Blank	K-HVW	K-HVW
$S_{Glucose}$ (%)	1.93	0.00	1.93	1.93
$S_{Fructose}$ (%)	0.00	0.00	0.00	0.00
$S_{Mannose}$ (%)	0.00	1.11	0.00	0.00
S_{DHA} (%)	0.00	1.67	0.00	1.45
$S_{GlycerAl}$ (%)	0.00	0.00	0.00	0.00
$S_{Pyruvaldehyde}$ (%)	0.00	15.01	0.00	0.00
S_{LA} (%)	59.92	0.00	60.41	60.89
S_{AcetAl} (%)	4.83	9.63	4.83	4.83
$S_{Acetic\ Acid}$ (%)	12.56	15.19	12.56	12.56
$S_{Glycolaldehyde}$ (%)	0.00	0.00	0.00	0.00
S_{GA} (%)	0.00	9.08	0.00	0.00
$S_{5\text{-}HMF}$ (%)	0.00	0.00	0.00	0.00
S_{FA} (%)	1.77	1.85	1.77	2.09
S_{LevA} (%)	1.61	1.85	1.61	1.61
$S_{Glyoxal}$ (%)	0.00	0.00	0.00	0.00
$S_{Succinic\ Acid}$ (%)	0.00	0.00	0.00	0.00
S_{CO_2} (%)	0.67	3.17	0.65	0.66

Lactic Acid

The stock solution for the catalyst was 0.2775 g catalyst and 0.9083 g lactic acid in 45 ml deionized water. The pH of the solution was 1.707.

Table S35: weight of catalyst, reactors and filled in solutions and pH measurements for the lactic acid in the pathway investigation. Reaction conditions: n(Substrate) = 2 mmol lactic acid; n(Catalyst) = 0.1 mmol vanadium; V = 10 mL, pN_2 = 20 bar; T = 200 °C; t = 1 h; $V_{stirrer}$ = level 6; Solvent: H_2O.

Catalyst	$m_{catalyst}$ (g)	pH_{before}	$m_{Reactor}$ (g)	$m_{solution}$ (g)	pH_{after}	$m_{solution.after}$ (g)
K-HVW	-	-	340.721	10.284	2.073	10.076
K-HVW	-	-	336.824	10.285	1.993	10.141
K-HVW	-	-	336.182	10.285	1.997	10.260
Blank	-	2.037	333.997	10.202	2.216	10.078

Table S36: calculated yields of all detected products for the lactic acid in the pathway investigation. Reaction conditions: n(Substrate) = 2 mmol lactic acid; n(Catalyst) = 0.1 mmol vanadium; V = 10 mL, pN_2 = 20 bar; T = 200 °C; t = 1 h; $V_{stirrer}$ = level 6; Solvent: H_2O

Experiment	K-HVW	K-HVW	K-HVW	Blank
$Y_{Glucose}$ (%)	2.06	2.06	2.06	0.00
$Y_{Fructose}$ (%)	0.00	0.00	0.00	0.00
$Y_{Mannose}$ (%)	1.03	1.03	1.03	0.00
Y_{DHA} (%)	0.00	0.00	0.00	0.00
$Y_{GlycerAl}$ (%)	0.00	0.00	0.00	0.00
$Y_{Pyruvaldehyde}$ (%)	0.00	0.00	0.00	0.00
Y_{LA} (%)	99.76	99.25	97.71	101.31
Y_{AcetAl} (%)	0.00	0.00	0.00	0.00
$Y_{Acetic Acid}$ (%)	0.00	0.00	0.00	0.00
$Y_{Glycolaldehyde}$ (%)	0.00	0.00	0.00	0.00
Y_{GA} (%)	0.00	0.00	0.00	0.00
Y_{5-HMF} (%)	0.00	0.00	0.00	0.00
Y_{FA} (%)	0.00	0.00	0.00	0.00
Y_{LevA} (%)	0.00	0.00	0.00	0.00
$Y_{Glyoxal}$ (%)	0.00	0.00	0.00	0.00
$Y_{Succinic Acid}$ (%)	0.00	0.00	0.00	0.00
Y_{CO_2} (%)	0.68	0.61	0.59	0.10
$Y_{Conversion}$ (%)	0.24	0.75	2.29	-1.31
$Y_{Carbon Balance}$ (%)	103.53	102.94	101.38	101.40

Oxidation state of the catalyst

All diagrams in the main manuscript are shown in the colour code: yellow for the substrate, green for the pathway to lactic acid, red for the pathway via 5-HMF, purple for the pathway to glycolic acid, and blue for the subsequent pathways of lactic acid. Values are the average of three experiments. The error bars represent the standard deviation. All reactions were performed with the same conditions of 200°C for one hour, a glucose concentration 0.1 mol L^{-1} and a catalyst concentration of 0.01 mol L^{-1} (based on the vanadium content).

The stock solution was 2.1617 g glucose in 120 ml deionized water. The weight for the catalyst solutions were 0.3200 g oxidized catalyst or 0.2146 g reduced catalyst in 35 ml glucose-water solution. The pH value of the reduced catalyst solution was 2.477. The pH for the oxidized catalyst was 1.854.

Table S37: weight of catalyst, reactors and filled in solutions and pH measurements for the comparison of oxidized vs reduced catalyst. Reaction conditions: n(Substrate) = 1 mmol glucose; n(Catalyst) = 0.1 mmol vanadium; V = 10 mL, pN_2 = 20 bar; T = 200 °C; t = 1 h; $V_{stirrer}$ = level 6; Solvent: H_2O.

Catalyst	$m_{catalyst}$ (g)	pH_{before}	$m_{Reactor}$ (g)	$m_{solution}$ (g)	pH_{after}	$m_{solution.after}$ (g)
red. K-HVW	-	-	341.316	10.213	2.233	10.213
ox. K-HVW	-	-	340.725	-	2.099	10.554
ox. K-HVW	-	-	340.544	10.152	2.103	10.008
Blank	-	5.836	340.782	10.068	2.842	9.949
ox. K-HVW	-	-	336.823	10.199	2.110	9.912
red. K-HVW	-	-	336.125	10.139	2.242	9.913

Table S38: calculated yields of all detected products for the comparison of oxidized vs reduced catalyst. Reaction conditions: n(Substrate) = 1 mmol glucose; n(Catalyst) = 0.1 mmol vanadium; V = 10 mL, pN_2 = 20 bar; T = 200 °C; t = 1 h; $V_{stirrer}$ = level 6; Solvent: H_2O.

Experiment	red. K-HVW	ox. K-HVW	ox. K-HVW	Blank	ox. K-HVW	red. K-HVW
$Y_{Glucose}$ (%)	3.00	3.00	3.00	46.00	3.00	3.00
$Y_{Fructose}$ (%)	0.00	0.00	0.00	0.00	0.00	0.00
$Y_{Mannose}$ (%)	1.00	1.00	1.00	7.00	1.00	1.00
Y_{DHA} (%)	0.00	0.00	0.00	2.00	0.00	0.00
$Y_{GlycerAl}$ (%)	1.50	2.00	2.00	0.00	2.00	2.00
$Y_{Pyruvaldehyde}$ (%)	16.50	17.00	17.00	16.50	17.00	16.00
Y_{LA} (%)	27.00	27.50	27.00	0.00	28.00	30.00
Y_{AcetAl} (%)	0.00	0.00	0.00	0.00	0.00	0.00
$Y_{Acetic\ Acid}$ (%)	5.33	5.00	5.00	4.00	5.33	4.67
$Y_{Glycolaldehyde}$ (%)	0.00	1.00	1.00	1.33	1.00	0.00
Y_{GA} (%)	0.00	0.00	0.00	0.00	0.00	0.00
$Y_{5\text{-}HMF}$ (%)	5.00	3.00	3.00	19.00	3.00	2.00
Y_{FA} (%)	7.50	8.17	8.33	1.67	8.33	6.67
Y_{LevA} (%)	15.00	20.00	20.00	0.83	20.00	15.83
$Y_{Glyoxal}$ (%)	0.00	0.00	0.00	0.00	0.00	0.00
$Y_{Succinic\ Acid}$ (%)	2.00	0.00	0.00	6.00	0.00	2.00
Y_{CO_2} (%)	1.97	2.16	2.01	0.88	2.47	2.30
$Y_{Conversion}$ (%)	97.00	97.00	97.00	54.00	97.00	97.00
$Y_{Carbon\ Balance}$ (%)	85.80	89.83	89.34	105.21	91.14	85.46

Table S39: calculated selectivities of all detected products for the comparison of oxidized vs reduced catalyst. Reaction conditions: n(Substrate) = 1 mmol glucose; n(Catalyst) = 0.1 mmol vanadium; V = 10 mL, pN_2 = 20 bar; T = 200 °C; t = 1 h; $V_{stirrer}$ = level 6; Solvent: H_2O.

Experiment	red. K-HVW	ox. K-HVW	ox. K-HVW	Blank	ox. K-HVW	red. K-HVW
$S_{Glucose}$ (%)	3.09	3.09	3.09	85.19	3.09	3.09
$S_{Fructose}$ (%)	0.00	0.00	0.00	0.00	0.00	0.00
$S_{Mannose}$ (%)	1.03	1.03	1.03	12.96	1.03	1.03
S_{DHA} (%)	0.00	0.00	0.00	3.70	0.00	0.00
$S_{GlycerAl}$ (%)	1.55	2.06	2.06	0.00	2.06	2.06
$S_{Pyruvaldehyde}$ (%)	17.01	17.53	0.00	30.56	17.53	16.49
S_{LA} (%)	27.84	28.35	27.84	0.00	28.87	30.93
S_{AcetAl} (%)	0.00	0.00	0.00	0.00	0.00	0.00
$S_{Acetic\ Acid}$ (%)	5.50	5.15	5.15	7.41	5.50	4.81
$S_{Glycolaldehyde}$ (%)	0.00	1.03	1.03	2.47	1.03	0.00
S_{GA} (%)	0.00	0.00	0.00	0.00	0.00	0.00
$S_{5\text{-}HMF}$ (%)	5.15	3.09	3.09	35.19	3.09	2.06
S_{FA} (%)	7.73	8.42	8.59	3.09	8.59	6.87
S_{LevA} (%)	15.46	20.62	20.62	1.54	20.62	16.32
$S_{Glyoxal}$ (%)	0.00	0.00	0.00	0.00	0.00	0.00
$S_{Succinic\ Acid}$ (%)	2.06	0.00	0.00	11.11	0.00	2.06
S_{CO_2} (%)	2.03	2.23	2.07	1.63	2.55	2.37

DFT calculated pK$_a$ values

DFT was used to calculate approximate pK$_a$ values, based on the ΔG of deprotonation. In the tables below, there are two calculated values for each successive deprotonation, as described in the table captions. The general equation used is:

$$\Delta G = [G(\mathrm{POM^-}) + G(\mathrm{H_3O^+})] - [G(\mathrm{POMH}) + G(\mathrm{H_2O})]$$

Table S40: Calculated pK$_a$ values (DFT), taken as the minimum calculated value for all combinations of proton removal.

Parent POM	pK$_a$ (1)	pK$_a$ (2)	pK$_a$ (3)	pK$_a$ (4)	pK$_a$ (5)
$H_6PV_3Mo_9O_{40}$	-5.67	-2.25	2.67	12.41	n/a
$H_6PV_3W_9O_{40}$	-9.14	-5.67	0.50	10.21	n/a
$H_9P_2V_3Mo_{15}O_{62}$	-11.11	-6.72	2.55	5.94	10.68
$H_9P_2V_3W_{15}O_{62}$	-13.96	-10.08	-0.91	3.52	8.43

Table S41: Calculated pK$_a$ values (DFT), taken as the average calculated value for all combinations of proton removal that have favourable ΔG.

Parent POM	pK$_a$ (1)	pK$_a$ (2)	pK$_a$ (3)	pK$_a$ (4)	pK$_a$ (5)
$H_6PV_3Mo_9O_{40}$	-4.26	-1.62	2.73	12.41	n/a
$H_6PV_3W_9O_{40}$	-5.90	-4.75	0.90	10.21	n/a
$H_9P_2V_3Mo_{15}O_{62}$	-6.49	-5.61	2.71	6.11	10.68
$H_9P_2V_3W_{15}O_{62}$	-7.88	-4.96	-0.89	3.52	8.43

References:

[1] A. H. Pawlig, W. You, M. J. Poller, J. Albert, *Chemistry-A European Journal,* **2025**, *31*, e02844.

[2] A. Wesner, J.-C. Raabe, M. J. Poller, S. Meier, A. Riisager, J. Albert, *Chemistry-A European Journal,* **2024**, *30*, e202402649.
